# CCDC58 drives lung adenocarcinoma progression via the PI3K/AKT signaling pathway

**DOI:** 10.3389/fonc.2025.1619123

**Published:** 2025-09-10

**Authors:** Wenchao Dai, Jun Yang, Wenming Yang, Guibin Zhang, Hang Chen, Bi Ren, Xin Dang, Linfeng Xue, Li Jiang

**Affiliations:** ^1^ Department of Respiratory and Critical Care Medicine, Affiliated Hospital of North Sichuan Medical College, Nanchong, Sichuan, China; ^2^ North Sichuan Medical College, Nanchong, Sichuan, China; ^3^ Department of Pediatric Urology, West China Second University Hospital, Sichuan University, Chengdu, China

**Keywords:** CCDC58, PI3K/AKT signaling pathway, EMT, LUAD, cell proliferation, apoptosis

## Abstract

**Background:**

Previous studies have implicated Coiled-coil domain-containing 58 (CCDC58) in the malignant progression of hepatocellular carcinoma and breast cancer. However, its role in lung adenocarcinoma (LUAD) remains poorly understood.

**Methods:**

Bioinformatics analysis was employed to examine CCDC58 expression patterns in LUAD and their correlation with clinical features. We validated CCDC58 expression levels using quantitative real-time PCR (qPCR), Western Blot (WB), and immunohistochemistry staining (IHC). Furthermore, we assessed the impact of CCDC58 knockdown on LUAD cell behavior using proliferation assays, cell migration assays, wound healing assays, and flow cytometry. We explored the effects of CCDC58 knockdown on apoptotic proteins, epithelial-mesenchymal transition (EMT) markers, and PI3K/AKT signaling pathway components through WB. Finally, we evaluated the role of CCDC58 in tumor growth *in vivo* using a nude mouse xenograft model, with subsequent IHC analysis of tumor tissues.

**Results:**

CCDC58 showed significant upregulation in LUAD cell lines and clinical specimens, leading to poor prognosis. CCDC58 expression was identified significant correlation with tumor microenvironment. *In vitro*, suppressing CCDC58 expression significantly impaired the capacity of growth and migration of LUAD cells. CCDC58 knockdown inhibited EMT, promoted apoptosis, and induced G1- phase cell cycle arrest. Significantly, CCDC58 knockdown inhibited the activity of the PI3K/AKT signaling pathway. *In vivo*, CCDC58 knockdown suppressed tumor growth and enhanced apoptosis.

**Conclusions:**

Above all, this study reveals that CCDC58 plays multiple pro-tumorigenic roles in the progression of LUAD. These results enhance the understanding of LUAD pathogenesis and highlight CCDC58 as a potential therapeutic target and prognostic biomarker.

## 1 Introduction

Globally, lung cancer remains the primary contributor to cancer mortality, accounting for the most cases of cancer-associated deaths (1). Non-small cell lung cancer represents the most common histological classification of the disease, among which lung adenocarcinoma (LUAD) constitutes a major subtype (2, 3). LUAD is prone to metastasis, frequently spreading to lymph nodes, contralateral lung, and distant organs (4). Despite significant progress in targeted therapies and immunotherapy, clinical outcomes of LUAD patients remain unsatisfactory (5, 6). Therefore, discovering novel molecular targets is critical to enhance early diagnosis and develop more effective treatments for LUAD.

The coiled-coil domain is an evolutionarily conserved structural motif formed by two or more α-helices, which exists in approximately 10% of human proteins (7, 8). This domain regulate diverse physiological processes across tissues (9). Dysregulation of Coiled-coil domain-containing (CCDC) family genes (via abnormal expression, mutations, or epigenetic changes) correlates with cancer progression (10–14). For example, CCDC8 knockdown enhances breast cancer cell migration and invasion (11); CCDC65 demonstrates tumor suppressor activity in both gastric and lung cancers (12, 13); and CCDC85B promotes NSCLC via AKT/GSK3β/β-catenin signaling (14). These studies suggest that CCDC family proteins play complex and diverse roles in cancers, warranting further in-depth investigation.

Coiled-coil domain-containing 58 (CCDC58), also designated as Mitochondrial Matrix Import Factor 23 (MIX23), belongs to the CCDC protein family. This 144-amino acid protein is encoded by a gene on chromosome 3q21.1 (15). Emerging evidences underscore the oncogenic roles of CCDC58 across diverse cancer types. In breast cancer, CCDC58 expression can be promoted by circ-TRIO, accelerating tumor progression (16). In hepatocellular carcinoma (HCC), CCDC58 is overexpressed in tumors compared to normal adjacent tissue (NAT). This overexpression correlates with higher tumor grades and promotes malignant phenotypes (17, 18). CCDC58 also contributes to ovarian and endometrial carcinogenesis and is a candidate therapeutic target (19, 20). The PI3K/AKT pathway governs critical oncogenic processes such as proliferation, differentiation, and invasion (21, 22), which is central to LUAD pathogenesis. For instance, METTL3 drives LUAD progression via this signaling (23), and SLITRK6 enhances LUAD malignancy by activating this pathway (24). Although the association of CCDC58 with PI3K/AKT signaling has been revealed (25), its mechanistic role in LUAD remains uncharacterized. This study aims to investigate the biological functions of CCDC58 in LUAD progression and its underlying molecular mechanisms, providing novel insights and identifying a potential therapeutic target for LUAD treatment.

## 2 Materials and methods

### 2.1 Bioinformatics analysis

Clinical and molecular data for LUAD were acquired from The Cancer Genome Atlas (TCGA) database, and accessed through the GDC Data Portal (https://portal.gdc.cancer.gov/). Data preprocessing and analysis were conducted using Perl (v5.30.0) and R (v4.3.3). Differential expression analysis and clinical correlation assessments were conducted using the limma package in R. Survival analysis and Cox regression modeling (univariate and multivariate) were performed using the survival package. Functional enrichment analyses (KEGG and GSEA) were performed with thresholds of *p* < 0.05 and |log2FC| > 1. Tumor microenvironment (TME) profiles were analyzed using the ESTIMATE package, while immune cell infiltration levels were quantified via CIBERSORT to deconvolute specific immune cell subsets. Finally, the analysis of pan-cancer differences was done based on the TIMER database (http://timer.comp-genomics.org).

### 2.2 Tissue specimens and immunohistochemistry staining

The clinicopathological data and tissue specimens (histologically verified tumor and NAT) from 80 patients with LUAD were analyzed in this study. All participants underwent curative surgical resection at the Affiliated Hospital of North Sichuan Medical College between January 2019 and December 2020. Ethical approval for this retrospective cohort study (approval number: 2024ER270 - 1) including a waiver of informed consent granted by the Ethics Committee.

IHC for all formalin formalin-fixed and paraffin-embedded (FFPE) tissue sections was conducted following established protocols (26). Briefly, FFPE tissue sections underwent standard processing, including incubation with primary antibodies followed by biotin-conjugated secondary antibodies, and were finally mounted using neutral balsam. Two independent associate chief pathologists evaluated IHC results. IHC results were quantified using the Immunoreactive Score (IRS) system, which combines staining intensity and percentage of positive cells into a composite score (27). For animal tissues, we analyzed staining patterns by calculating the ratio of integrated optical density (IOD) to positive tissue area.

### 2.3 Cell culture and lentiviral transfection

The human LUAD cell lines (H1299 and A549) were obtained from Pricella (Wuhan, China). The H1299 was cultured in RPMI 1640 medium containing 10% fetal bovine serum (FBS), while the A549 cell line was maintained in Ham’s F - 12K medium with 10% FBS. The normal bronchial epithelial cell line BEAS - 2B was obtained from Biocode (Zhejiang, China), and was grown in high-glucose DMEM containing 10% FBS. Each cell line was maintained in a 37 °C incubator with 5% carbon dioxide (CO_2_) and high moisture levels.

Lentiviral vectors for CCDC58 knockdown (shCCDC58) and negative control vectors (shNC), both containing puromycin resistance markers and the green fluorescent protein (GFP) gene, were acquired from GENE (Shanghai, China). The target sequences were:

shNC: TTCTCCGAACGTGTCACGT;
shCCDC58-1: GCAGTCAGAACTGAATGTTGA;
shCCDC58-2: GAGTCTTTGATGGCAGCTCAT;
shCCDC58-3: GCGGGCTTTCTAGGATGATTT.

Following the manufacturer’s protocol, we performed lentiviral transduction and selected transfected cells using puromycin (4 μg/mL for 1 week, then 2 μg/mL for maintenance). Three stable CCDC58 knockdown groups (shCCDC58-1, shCCDC58-2, and shCCDC58-3) and one negative control group (shNC) were employed in this study.

### 2.4 Quantitative real-time PCR

According to the manufacturer’s guidelines, we conducted total RNA extraction, cDNA synthesis, and qPCR using the following kits (Accurate Biotechnology, Hunan, China): the SteadyPure Universal RNA Extraction Kit for RNA isolation, the Evo M-MLV RT Premix Kit for cDNA synthesis, and the SYBR Green Pro Taq HS Premixed qPCR Kit for amplification. Using the 2^(-ΔΔCT) method, we quantified relative gene expression (28). The oligonucleotide primer sequences designed for amplification of the target gene’s coding DNA sequence (CDS) are listed below:

CCDC58:
Forward: 5’-ATTGATGCCAGCCAAACCTG-3’;
Reverse: 5’-CTACTGCTGAAGTCTGGGCT-3’.
β-ACTIN:
Forward: 5’-CCTTCCTGGGCATGGAGTC-3’;
Reverse: 5’-TGATCTTCATTGTGCTGGGTG-3’.

### 2.5 Western Blot analysis

WB was conducted following established protocols (29). Briefly, primary antibody incubation was performed overnight on the prepared membranes, using the following specific antibodies: CCDC58 (OmnimAbs, 1:1000); β-ACTIN and GAPDH (Affinity, 1:5000 each); BAX, p-AKT, BCL - 2, AKT, N-cadherin, E-cadherin, and Vimentin (all HuaBio; 1:10000, 1:1000, 1:4000, 1:4,000, 1:5000, 1:5000, and 1:20,000, respectively); and PI3K and p-PI3K (Zenbio, 1:1500 each). Subsequently, the membranes were incubated to a horseradish-peroxidase-conjugated goat anti-rabbit IgG secondary antibody (dilution, 1:5000) from FineTest (Wuhan, China). Protein bands were visualized using an ECL substrate kit (Epizyme, Shanghai, China) on a ChemiDoc™ XRS+ system (Bio-Rad, USA) and quantified with ImageJ software.

### 2.6 Cell proliferation and colony formation assays

Cell proliferation was assessed via the CCK - 8 kit (APExBIO, USA) in 96-well plates containing 5,000 cells per well. Cell viability assessments were conducted at 0, 24, 48, and 72 hours. Following the manufacturer’s protocol, 10 μL of CCK - 8 solution was combined with 100 μL of medium in each well. Plates were incubated for 2 hours at 37 °C under 5% CO_2_ with controlled humidity, after which optical density was quantified at 450 nm.

In colony formation experiments, cells (1,000 per well) were seeded in 6-well plates. The cultures were incubated at 37 °C in a 5% CO_2_-enriched humidified environment for 7–14 days. Visible colonies were washed with ice-cold PBS, fixed with 4% paraformaldehyde (PFA) for 30 minutes, and stained for 20 minutes using 0.1% crystal violet. Colonies containing 50 or more cells were counted with ImageJ software.

### 2.7 Cell migration and wound healing assays

Transwell chambers with 8 μm pores (Corning, USA) were used to analyze cell migration. In short, 50,000 cells in a serum-free medium were placed in the upper chambers, with the lower chambers filled with complete medium containing 10% FBS to act as a chemoattractant. After being incubated for 24 hours at 37 °C in a humidified atmosphere containing 5% CO_2_, migrated cells were fixed with 4% PFA and stained with 0.1% crystal violet. Microscopic images were acquired and analyzed using ImageJ to quantify cell migration.

In wound healing experiments, 5 × 10^5^ cells per well were plated into 6-well plates and grown to 90% confluency before initiating the assay. Uniform wounds were then introduced into the monolayer using a sterile 200 μL pipette tip. After replacing the medium with serum-free conditions, we monitored wound closure by acquiring images at 0 and 48 hours post-wounding. Using ImageJ, we quantified cell migration by measuring the wound distance over time.

### 2.8 Apoptosis and cell cycle analysis

The cell apoptosis assay was performed when the cell confluence reached 70%. After harvesting, the cells were washed two times with ice-cold PBS and resuspended in Binding Buffer. Samples were analyzed with a flow cytometer (ACEA Bioscience, USA) after being incubated in the dark for 10 minutes at room temperature with Annexin V-APC/7-AAD staining solution (KeyGEN BioTECH, China).

For cell cycle profiling, harvested cells were washed with PBS, fixed in 70% ethanol, and treated with RNase and propidium iodide (KeyGEN BioTECH, China) for 60 minutes. Samples were analyzed using a flow cytometer (ACEA Bioscience, USA).

### 2.9 Animal experiments

Four-week-old male BALB/c nude mice were obtained from SPF Biotechnology (Beijing, China) and randomly divided into two groups (n = 5). A549 cells transfected with negative control shRNA (shNC) or CCDC58-targeting shRNA (shCCDC58-3) were subcutaneously injected into the right flank at 3 × 10^6^ cells per mouse. The tumor’s growth was tracked every three days using caliper measurements, and its volume was determined by the formula (length × width²)/2. After four weeks, mice were euthanized humanely, and xenografts were excised for downstream analysis. Prior to commencement, the study received full ethical clearance (NSMC Ethical Animal Review [2024] 127) from North Sichuan Medical College Animal Ethics Committee.

### 2.10 Statistical analysis

R statistical software (version 4.3.3) and GraphPad Prism (version 9.5) were used for statistical analyses. All experiments included three independent biological replicates, with quantitative data presented as mean ± standard deviation. Intergroup differences were statistically assessed using Student’s t-test, whereas multigroup comparisons required application of one-way ANOVA. To examine potential correlations between CCDC58 expression levels and clinicopathological characteristics, Pearson’s χ² test was implemented. A *p*-value less than 0.05 was considered statistically significant, with significance levels indicated as: *p < 0.05, **p < 0.01, ***p < 0.001.

## 3 Results

### 3.1 CCDC58 is highly expressed in LUAD

**Figure 1:**
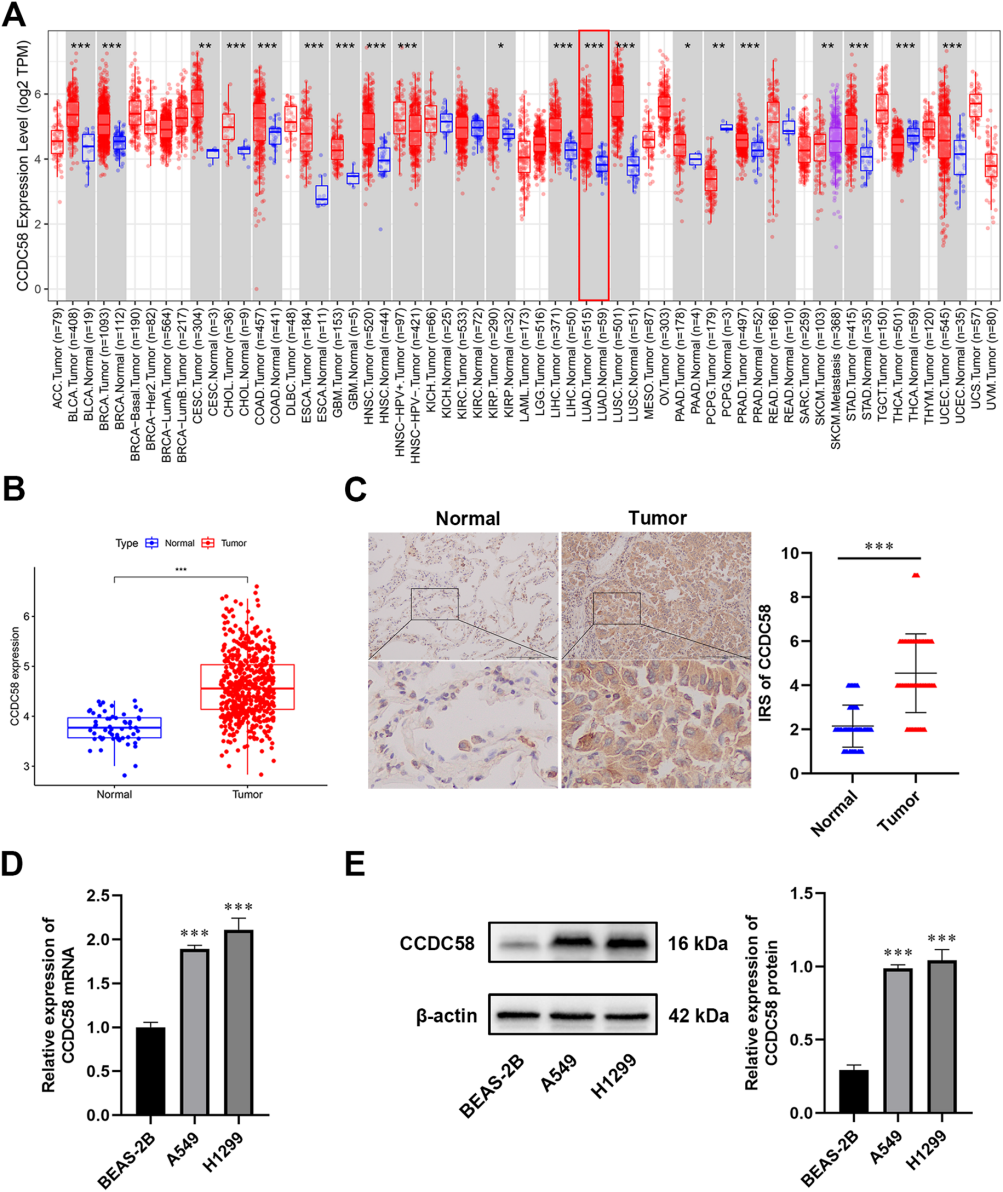
CCDC58 is highly expressed in LUAD tissues and cell lines. **(A)** Pan-cancer analysis of CCDC58; **(B)** Differential expression of CCDC58 in TCGA database; **(C)** IHC of CCDC58 in LUAD and NAT (SP × 200); **(D, E)** mRNA and protein expression of CCDC58 in cell lines. *p < 0.05, **p < 0.01, ***p < 0.001.

Pan-cancer analysis identified CCDC58 overexpression in LUAD (**Figure 1A**), which was validated in TCGA datasets showing significantly higher CCDC58 levels in LUAD versus NAT (**Figure 1B**). IHC of 80 paired LUAD/NAT confirmed this overexpression pattern (**Figure 1C**). CCDC58 expression was significantly upregulated in LUAD cell lines (H1299, A549) compared to normal bronchial epithelial BEAS - 2B cell line, with consistent increases observed at both mRNA and protein levels (**Figures 1D, E**). These consistent findings across bioinformatics, clinical samples, and cells suggest that CCDC58 plays a critical role in LUAD pathogenesis.

### 3.2 CCDC58 correlates with poor prognosis and advanced disease features

**Figure 2:**
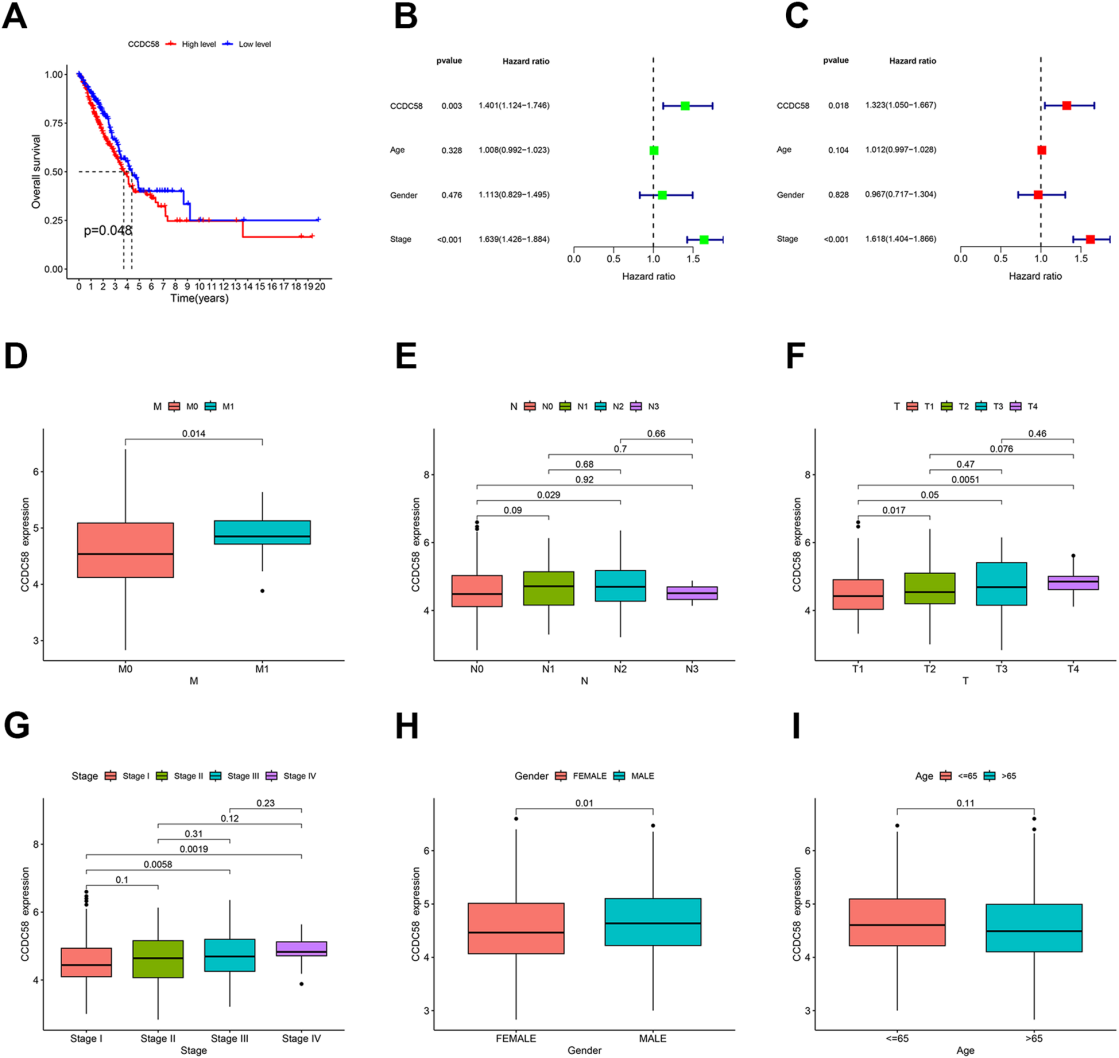
Survival analysis and clinicopathological correlation of CCDC58. **(A)** Survival analysis of CCDC58; (**B, C)** Univariate and multivariate COX regression analyses; **(D–I)** Correlation between CCDC58 expression and clinicopathological characteristics. *p < 0.05, **p < 0.01, ***p < 0.001.

Analysis of the TCGA dataset using bioinformatics revealed that higher CCDC58 expression was linked to poor overall survival (**Figure 2A**, **Supplementary Figure S1**). CCDC58 emerged as an independent predictor of prognosis in both univariate and multivariate Cox regression analyses, demonstrating clinical relevance comparable to tumor stage (**Figures 2B, C**). CCDC58 expression patterns demonstrated significant clinicopathological correlations, with particularly elevated levels observed in metastatic (M1) compared to non-metastatic (M0) cases (**Figure 2D**), in N2 versus N0 cases (**Figure 2E**), and in T2/T4 versus T1 cases (**Figure 2F**). The protein was upregulated in advanced-stage (III/IV) compared to early-stage (I) disease (**Figure 2G**) and exhibited higher expression in male patients (**Figure 2H**). No significant age-dependent differences were observed (**Figure 2I**). In our institutional LUAD cohort, CCDC58 expression levels showed significant associations with T stage and overall stage, but not with N stage, gender, age, or smoking history (**Table 1**). The partial discordance with TCGA data may reflect our cohort’s N0 predominance and limited sample size. In a word, CCDC58 may be a key clinical marker for tumor growth and metastasis, and can independently predict a poor prognosis for patients in LUAD.

**Table 1:** Correlation between CCDC58 expression and clinicopathological characteristics.

Items	Total cases (n=80)	IRS score		*p*-value
		Low express (n=48)	High express (n=32)	
Age				
<65	46	31	15	
≥65	34	17	17	0.1165
Gender				
Male	35	20	15	
Female	45	28	17	0.6455
Smoking history				
No	52	29	23	
Yes	28	19	9	0.2925
T stage				
T1	56	38	18	
T2+T3+T4	24	10	14	0.0284^*^
N stage				
N0	59	39	20	
N1+N2+N3	21	9	12	0.0619
M stage				
M0+M1	80	48	32	/
Clinical stage				
I	51	36	15	
II+III+IV	29	12	17	0.0104^*^

A *p*-value less than 0.05 was considered statistically significant, with significance levels indicated as: *p < 0.05, **p < 0.01, ***p < 0.001.

### 3.3 CCDC58 influences cell cycle and correlates with TME

**Figure 3:**
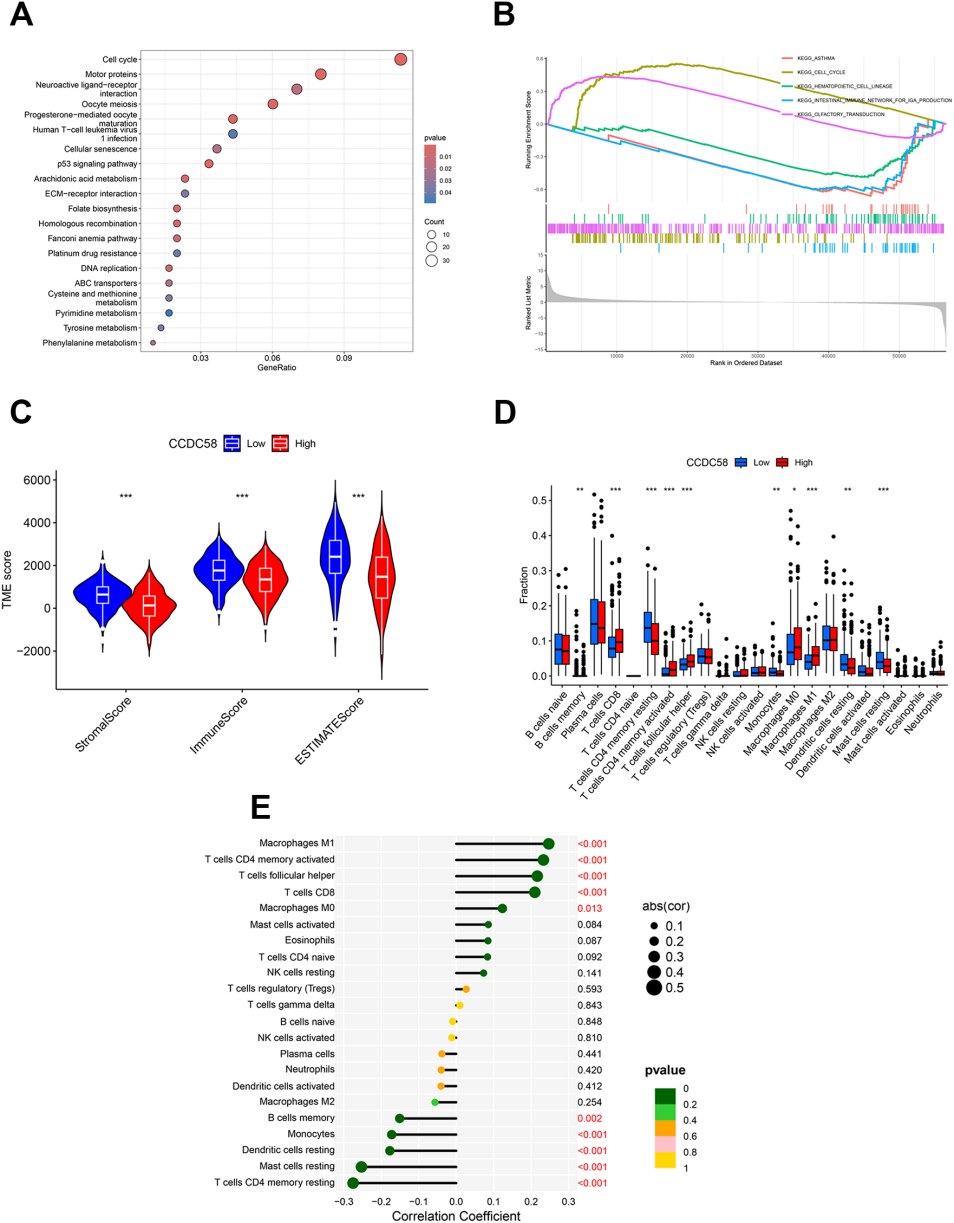
Functional enrichment and TME analysis. **(A, B)** KEGG and GSEA enrichment analyses; (**C–E)** TME analysis. *p < 0.05, **p < 0.01, ***p < 0.001.

KEGG pathway analysis demonstrated significant enrichment of CCDC58-associated genes in cell cycle regulation pathways (**Figure 3A**). Enrichment of cell cycle-related genes was demonstrated in LUAD specimens with elevated CCDC58 expression through GSEA (**Figure 3B**), consistent with prior observations. Furthermore, CCDC58 expression levels significantly influenced TME composition, as evidenced by alterations in Stromal, Immune, and ESTIMATE scores (**Figure 3C**). Immune cell infiltration analysis of 10 cell subtypes showed distinct correlation patterns with CCDC58 expression levels (**Figure 3D**). Specifically, higher CCDC58 expression positively correlated with increased infiltration of activated Macrophages M1, T cells CD4 memory activated, T cells follicular helper, T cells CD8 and Macrophages M0, while showing negative correlations with B cells memory, Monocytes, Dendritic cells resting, Mast cells resting and T cells CD4 memory resting (**Figure 3E**). These findings suggest CCDC58 promotes LUAD progression through dual mechanisms, including influence of cell cycle and immunomodulation of TME.

### 3.4 CCDC58 knockdown suppresses LUAD cell proliferation

**Figure 4:**
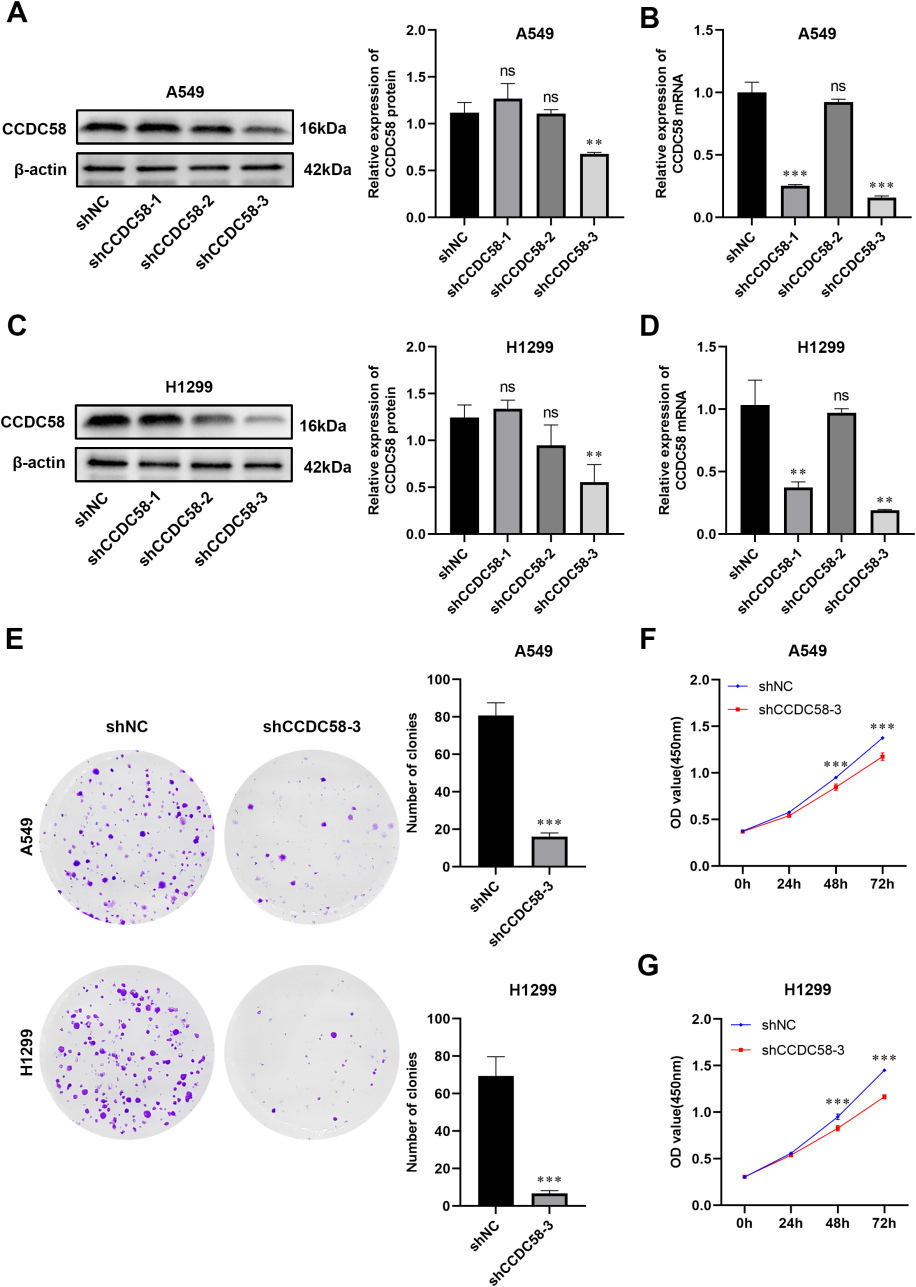
CCDC58 knockdown suppresses cell proliferation. **(A–D)** Knockdown efficiency of CCDC58 assessed by qPCR and WB; **(E–G)** Colony formation and cell proliferation assays. *p < 0.05, **p < 0.01, ***p < 0.001. ns, not significant.

We generated three lentiviral shRNA constructs targeting CCDC58 (shCCDC58-1, -2, -3) in LUAD cell lines. Based on validation by qPCR and WB, shCCDC58–3 showed optimal knockdown efficiency (**Figures 4A–D**) and was selected for functional studies. CCDC58 knockdown produced significant anti-tumor effects, reducing colony formation capacity (**Figure 4E**) and decreasing cell proliferation (**Figures 4F, G**) compared to controls. These results establish CCDC58 as a critical regulator of LUAD cell growth and clonogenicity, highlighting its therapeutic potential.

### 3.5 CCDC58 knockdown impairs cell migration and reverses epithelial-mesenchymal transition markers

**Figure 5:**
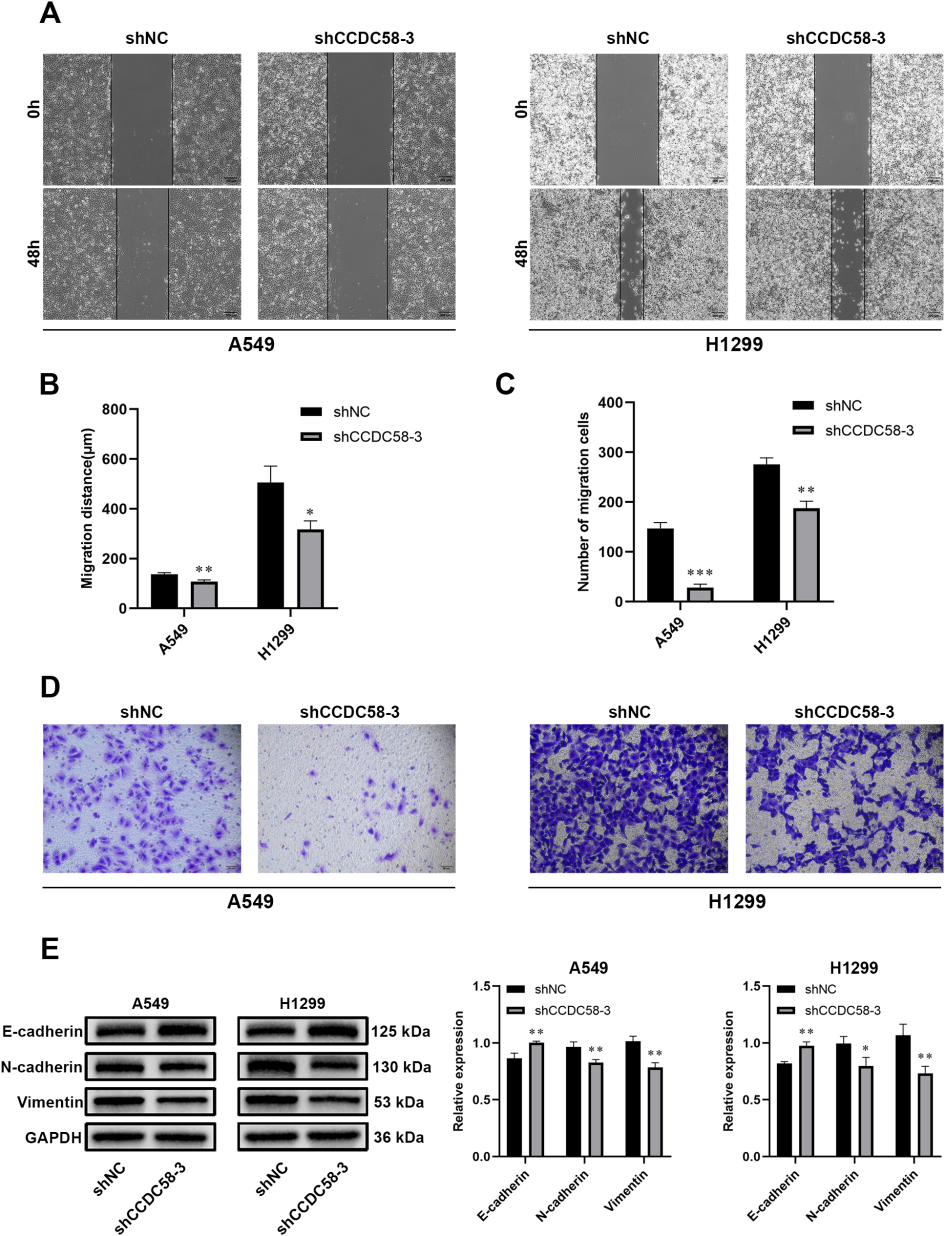
Effect of CCDC58 on cell migration. **(A–D)** Wound healing and cell migration assays for assessing cell migration ability; **(E)** CCDC58 knockdown influences EMT marker protein expression. *p < 0.05, **p < 0.01, ***p < 0.001.

Functional assays demonstrated that CCDC58 knockdown significantly impaired cell motility, reducing wound closure (**Figures 5A, B**) and decreasing migration capability (**Figures 5C, D**) compared to controls. Considering the pivotal regulatory role of EMT in tumor aggressive growth and distant metastasis in malignancies (30), we analyzed EMT marker expression by WB. CCDC58 knockdown influenced EMT, characterized by E-cadherin upregulation concurrent with N-cadherin and vimentin downregulation (**Figure 5E**). These findings demonstrate that CCDC58 promotes LUAD cell motility through EMT regulation, suggesting its potential role in metastatic progression.

### 3.6 CCDC58 knockdown induces cell cycle arrest and apoptosis

**Figure 6:**
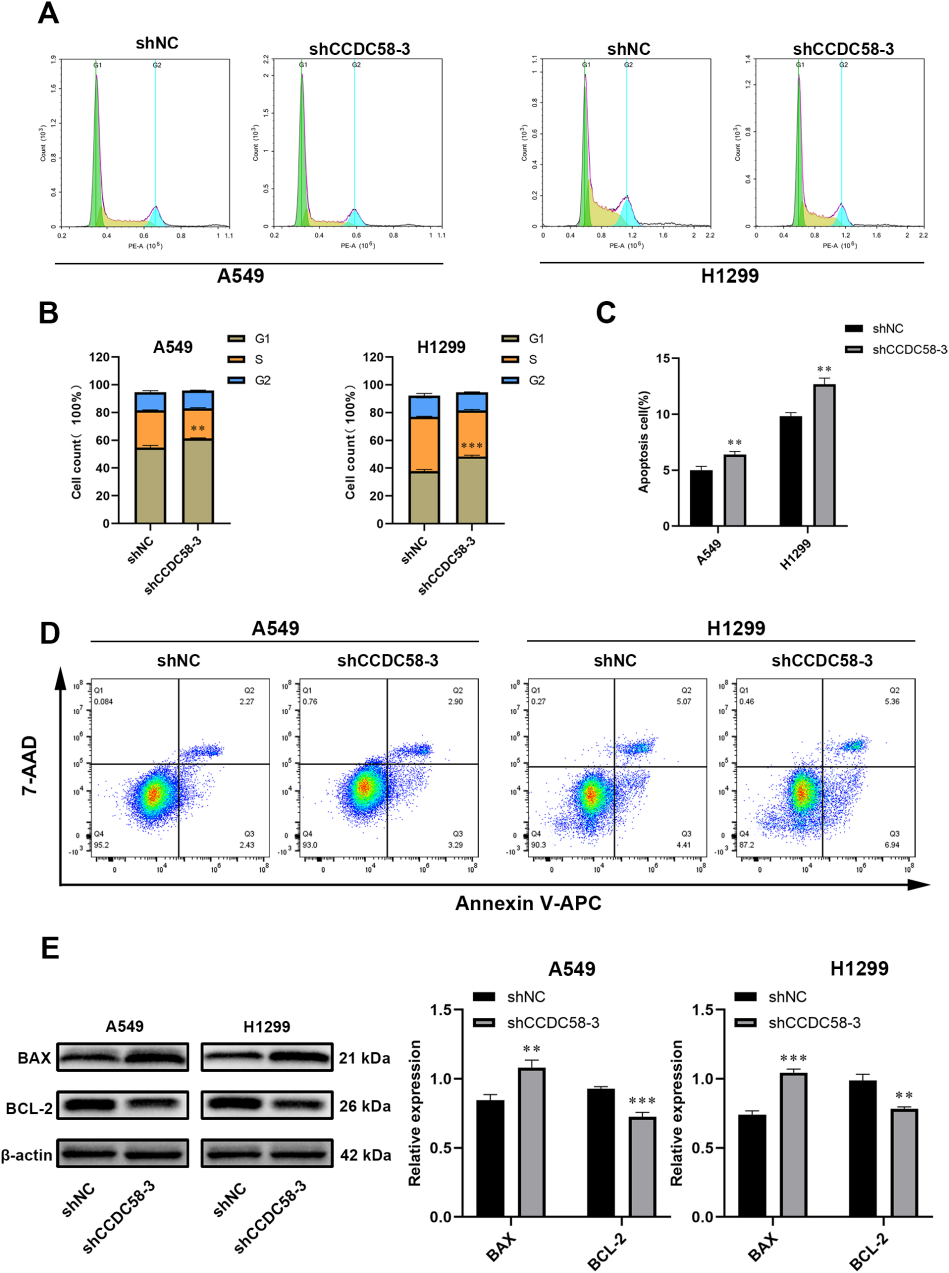
Effect of CCDC58 on cell cycle and apoptosis. **(A, B)** CCDC58 knockdown influences cell cycle; **(C–E)** CCDC58 knockdown induces apoptosis. *p < 0.05, **p < 0.01, ***p < 0.001.

Cell cycle analysis by flow cytometry demonstrated that CCDC58 knockdown induced G1-phase arrest in both H1299 and A549 cell lines (**Figures 6A, B**). Concomitantly, apoptotic rates were significantly increased in these cell lines (**Figures 6C, D**). To understand how CCDC58 influences apoptosis at the molecular level, we used WB to study the expression of proteins associated with apoptosis. In results, knocking down CCDC58 suppressed the expression of anti-apoptotic protein BCL - 2 and amplified the expression of pro-apoptotic protein BAX (**Figure 6E**). These results indicate that CCDC58 not only affects the cell cycle but also plays a significant role in apoptosis.

### 3.7 CCDC58 knockdown inhibits PI3K/AKT signaling pathway activity

**Figure 7:**
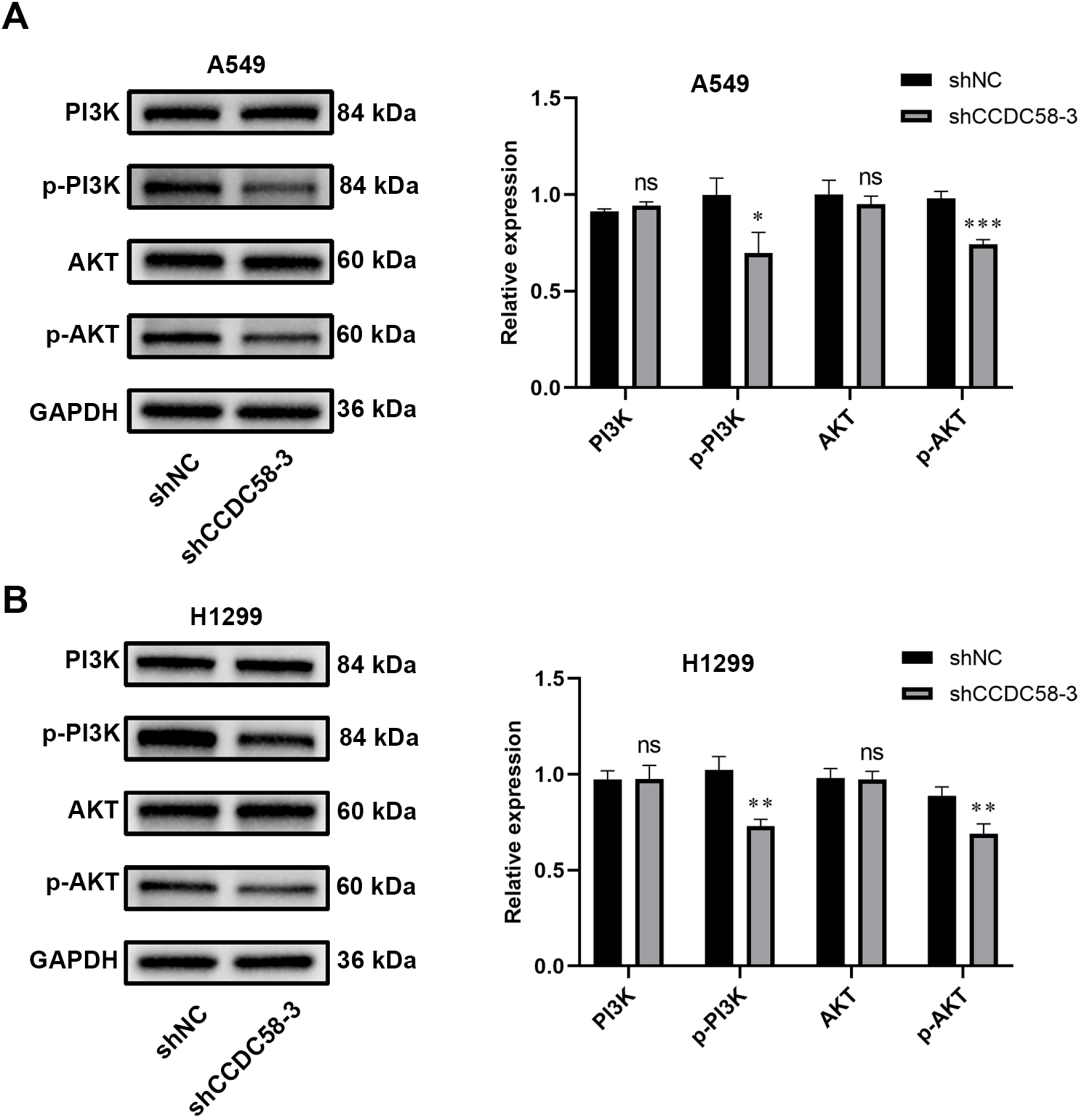
CCDC58 influences the PI3K/AKT signaling pathway. **(A, B)** CCDC58 knockdown reduced p-PI3K and p-AKT protein expression. *p < 0.05, **p < 0.01, ***p < 0.001. ns, not significant.

To investigate how CCDC58 influences LUAD progression, we examined its effect on tumor-related signaling pathways. Based on previous studies linking CCDC58 to the PI3K/AKT pathway (25), we analyzed key pathway components by WB. CCDC58 knockdown significantly reduced p-PI3K and p-AKT (**Figures 7A, B**), suggesting CCDC58 promotes LUAD progression through influencing PI3K/AKT pathway activity.

### 3.8 CCDC58 knockdown inhibits tumor growth

**Figure 8:**
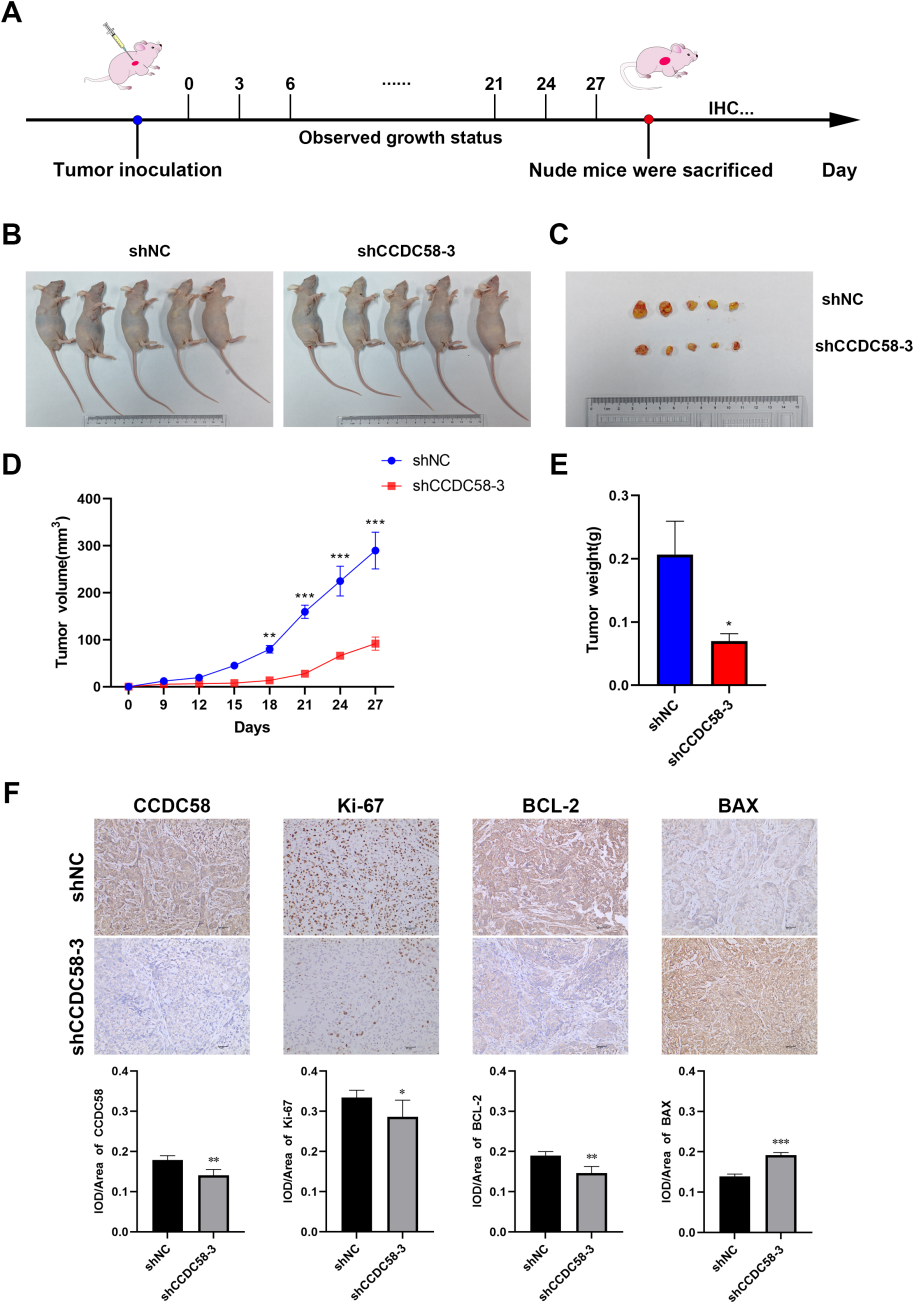
Effect of CCDC58 on tumor growth. **(A)** Scheme of the experimental design; **(B)** Images of the control and CCDC58 knockdown group mice; **(C)** Excised tumors from the control and CCDC58 knockdown groups; **(D)** Tumor growth curves; **(E)** Excised tumor weights; **(F)** IHC images and scores of tumor tissues (SP×200). *p < 0.05, **p < 0.01, ***p < 0.001.

To investigate CCDC58’s role in LUAD tumorigenesis, we developed a xenograft model by subcutaneously injecting stably transfected A549 cells into nude mice which were randomized into control (shNC) and CCDC58-knockdown (shCCDC58-3) groups (**Figures 8A, B**). CCDC58 suppression significantly inhibited tumor growth, reducing tumor volume (**Figures 8C, D**) and decreasing final tumor weight (**Figure 8E**) compared to controls. IHC analysis demonstrated that CCDC58 knockdown reduced proliferation (decreased Ki-67 protein expression) while promoting apoptosis (increased BAX and decreased BCL - 2 protein expression) (**Figure 8F**). These results establish CCDC58’s dual oncogenic function in LUAD by simultaneously promoting proliferation and suppressing apoptosis.

## 4 Discussion

This study provides the first integrated analysis of CCDC58 expression and clinical relevance in LUAD through combined bioinformatics and experimental approaches. We identified consistent CCDC58 upregulation in LUAD cell lines and tissues. Analysis of TCGA data revealed significant associations between CCDC58 expression and TNM/Prognosis classifications, which were further supported by clinicopathological data from our institutional cohort showing correlations with tumor size and Prognosis. However, unlike the TCGA findings, we observed no significant association with patient gender in our cohort. The observed discrepancies in N-stage and gender correlations may reflect differences in sample composition, particularly the predominance of N0-stage cases and smaller sample size in our study. Through univariate and multivariate Cox regression analyses, CCDC58 emerged as a prognostic marker. These results collectively implicate CCDC58 as a potential driver of LUAD progression and a candidate prognostic marker. Notably, our findings align with reported oncogenic roles of CCDC58 in other cancers. Previous studies demonstrated its tumor-promoting effects in breast cancer (16) and its dual role in tumor grade association and proliferation/apoptosis regulation in HCC (17, 18). These consistent findings further strengthen the potential role of CCDC58 as a key regulatory molecule in tumor progression. In these findings, CCDC58 could serve as both a diagnostic marker and therapeutic target for LUAD, potentially informing novel small-molecule inhibitor development.

Although CCDC58 has been implicated in breast and hepatocellular carcinogenesis, its role in LUAD pathogenesis remained unknown until now. In this study, CCDC58 knockdown was found to inhibit cell proliferation and migration capabilities. Given that Ki-67 serves as a biomarker for cell proliferation (31), we performed ICH analysis on xenograft tumors in nude mice, which demonstrated that CCDC58 knockdown reduced Ki-67 protein expression. Through preliminary bioinformatics analysis, we identified that CCDC58-associated genes were significantly enriched in the cell cycle pathway. *In vitro*, CCDC58 knockdown resulted in cell cycle arrest at the G1 phase. Additionally, our experiments demonstrated that reducing CCDC58 expression enhanced apoptosis. Both *in vivo* and *in vitro* experiments showed that CCDC58 knockdown upregulated the expression of the pro-apoptotic protein BAX while downregulating the anti-apoptotic protein BCL - 2. Notably, BAX and BCL - 2 are key regulators of apoptosis (32, 33). Above all, CCDC58 may facilitate LUAD progression by promoting cell proliferation and migration, influencing the cell cycle, and suppressing apoptosis.

Metastasis remains the primary contributor to cancer-related mortality, with EMT established as a critical mechanistic driver (34–36). This biological process is defined by the progressive loss of epithelial markers like E-cadherin accompanied by simultaneous acquisition of mesenchymal markers including N-cadherin and vimentin (37). Our experimental results showed that CCDC58 knockdown produces an increase in E-cadherin expression coupled with reduction in both N-cadherin and vimentin levels. This coordinated reversal of EMT marker expression strongly implicates CCDC58 in promoting LUAD progression through activation of the EMT process, which would enhance tumor cell motility and metastatic dissemination.

The PI3K/AKT signaling regulates essential cancer-related biological functions, including cell proliferative, differentiation, and metastasis activity (38, 39). In LUAD specifically, this pathway has been established as a key driver of tumor progression (23, 24). Based on previous reports of CCDC58’s association with PI3K/AKT signaling (25), we speculated that CCDC58 exerts its oncogenic effects in LUAD through PI3K/AKT pathway. Subsequently, we found that CCDC58 knockdown markedly decreases both p-AKT and p-PI3K. In short, these findings establish CCDC58 as a novel upstream regulator of PI3K/AKT signaling in LUAD pathogenesis. CCDC58 promotes malignant phenotypes in LUAD cells via this signaling pathway. Future researches involving CCDC58 overexpression could validate these findings.

Increasing evidences highlight the prognostic significance of TME composition in lung cancer (40). We examined how CCDC58 expression is related to the TME in LUAD. Individuals with elevated CCDC58 expression exhibited unique TME features, such as notable variations in Stromal Score, Immune Score, and ESTIMATE Score compared to those with low expression. Notably, CCDC58 expression levels correlated with the infiltration densities of ten distinct immune cell types, with five showing positive or negative correlations, respectively. This dichotomous immunomodulatory pattern suggests CCDC58 may promote tumor progression by altering the immune landscape, potentially explaining its association with adverse clinical outcomes.

Although this study has yielded significant findings, several limitations should be acknowledged. First, discrepancies were observed in the clinicopathological characteristics between the TCGA database and our hospital’s patient cohort, which may be attributed to the smaller sample size of our clinical specimens. The relatively small sample size may limit the statistical power of our findings. Second, while we identified that CCDC58 influences the PI3K/AKT signaling pathway, the precise molecular mechanism by which CCDC58 directly or indirectly activates this pathway remains incompletely elucidated. Third, while we investigated CCDC58’s impact on the TME, our xenograft experiments did not examine its specific effects on immune system-mediated TME modulation.

To sum up, our findings indicate that CCDC58 is expressed irregularly in LUAD and is related to adverse outcomes. We have elucidated its impact on the biological behavior of LUAD and explored potential underlying mechanisms, confirming the oncogenic role of CCDC58 in LUAD progression. According to these findings, CCDC58 could potentially be a target for diagnosis and therapy in LUAD.

## Data Availability

The raw data supporting the conclusions of this article will be made available by the authors, without undue reservation.
