# Supplementary material for: CCDC58 drives lung adenocarcinoma progression via the PI3K/AKT signaling pathway
Source: Front Oncol. 2025 Sep 10;15:1619123. doi: 10.3389/fonc.2025.1619123 (PMC12457109; doi:10.3389/fonc.2025.1619123)
Supplement: Supplementary file 1 [file DataSheet1.docx]

**
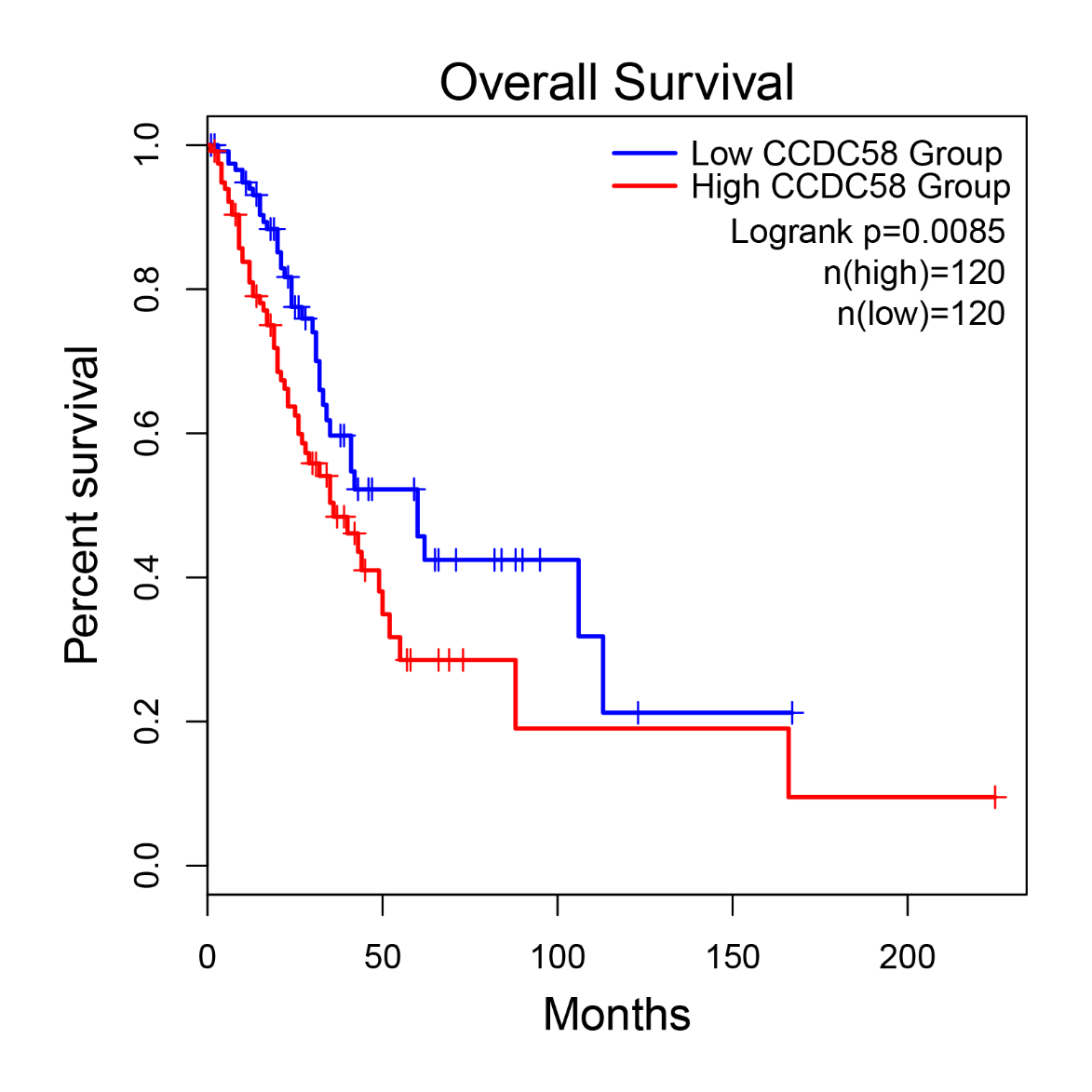
**

**Figure S1** Survival analysis

Survival analysis was performed based on the GEPIA 2 database (http://gepia2.cancer-pku.cn/). **p* < 0.05, ***p* < 0.01, ****p* < 0.001.
