# Supplementary material for: CCDC58 drives lung adenocarcinoma progression via the PI3K/AKT signaling pathway
Source: Front Oncol. 2025 Sep 10;15:1619123. doi: 10.3389/fonc.2025.1619123 (PMC12457109; doi:10.3389/fonc.2025.1619123)

Figure 1E protein expression of CCDC58 in cell lines

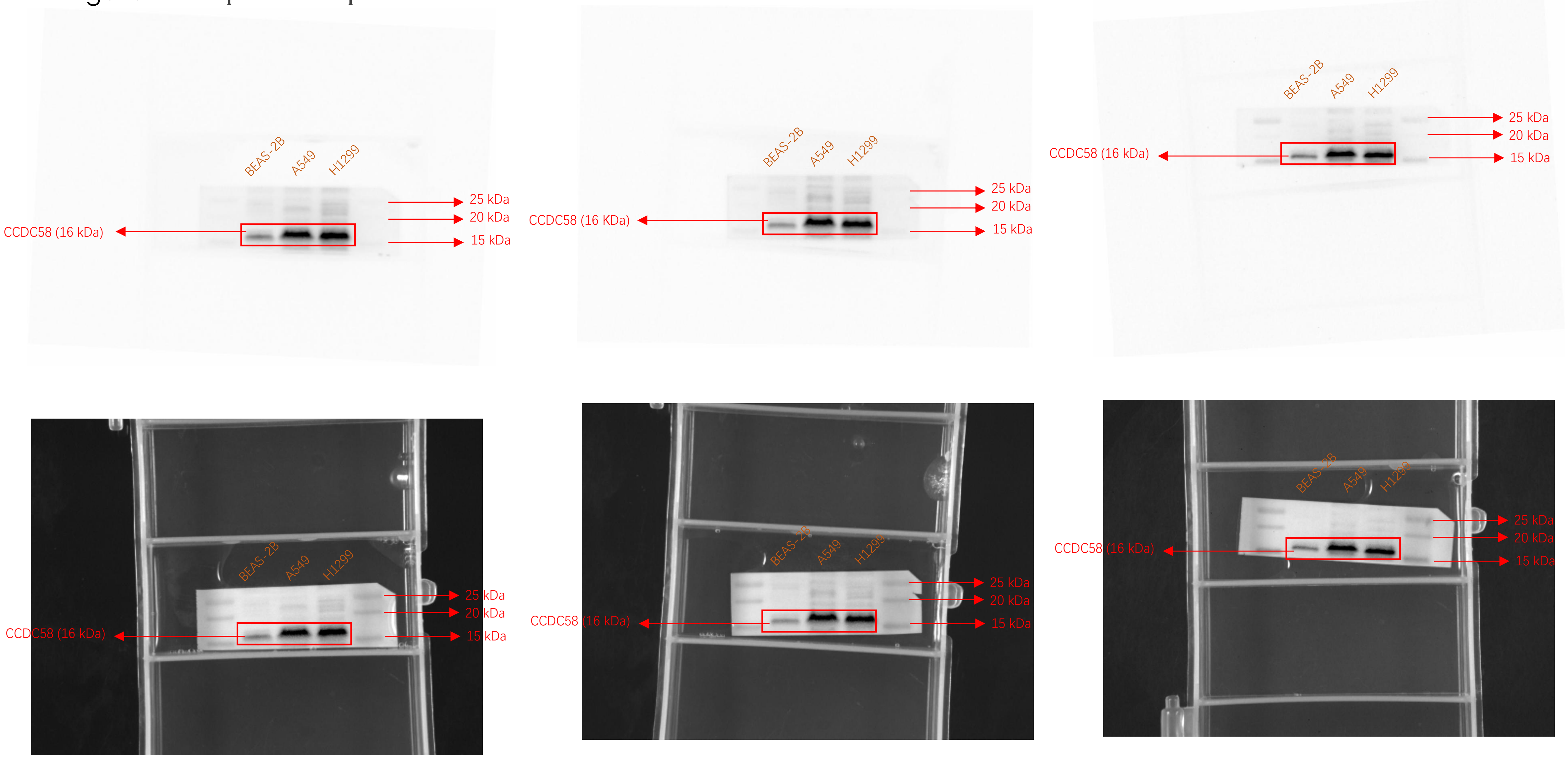

Internal Control of CCDC58

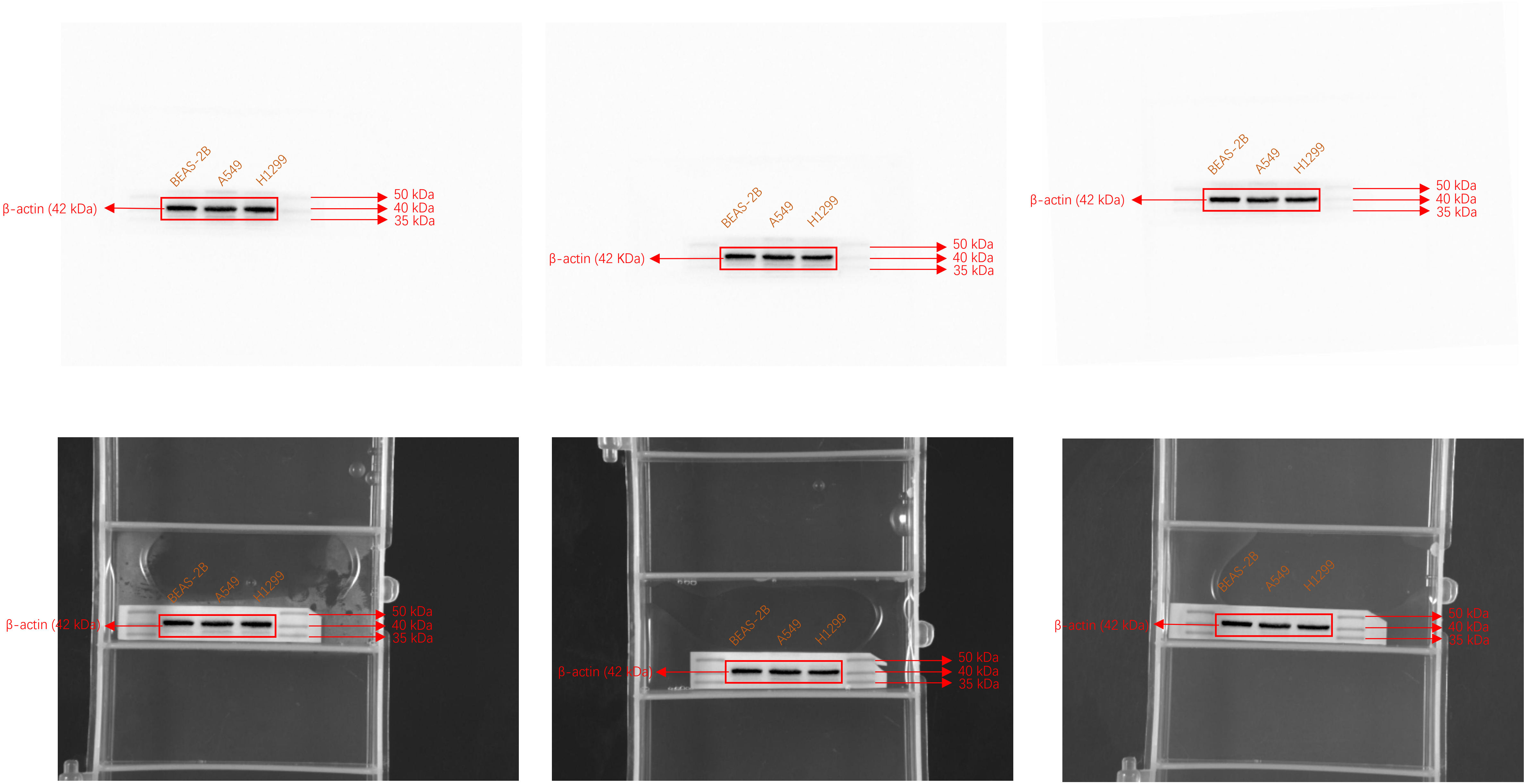

Figure 4A Knockdown efficiency of CCDC58 in A549

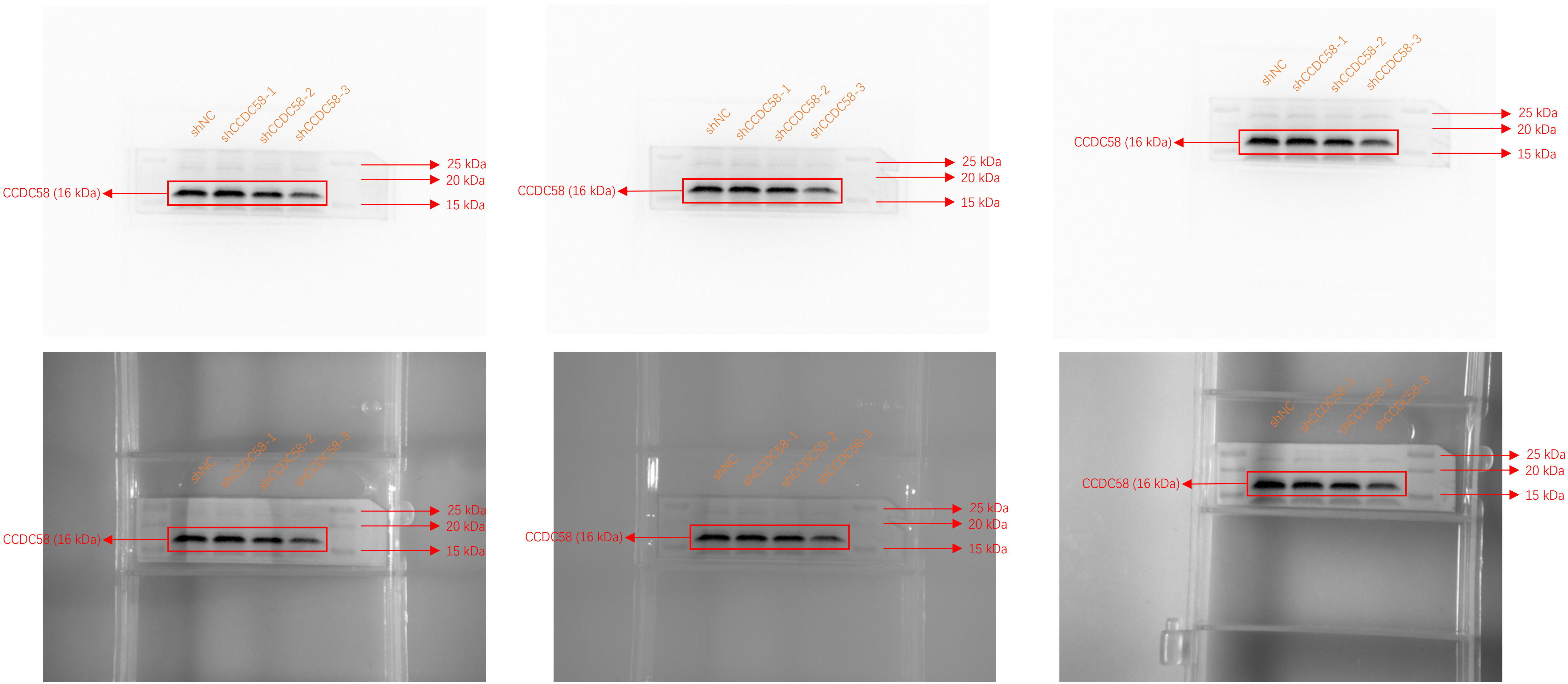

Internal Control of CCDC58

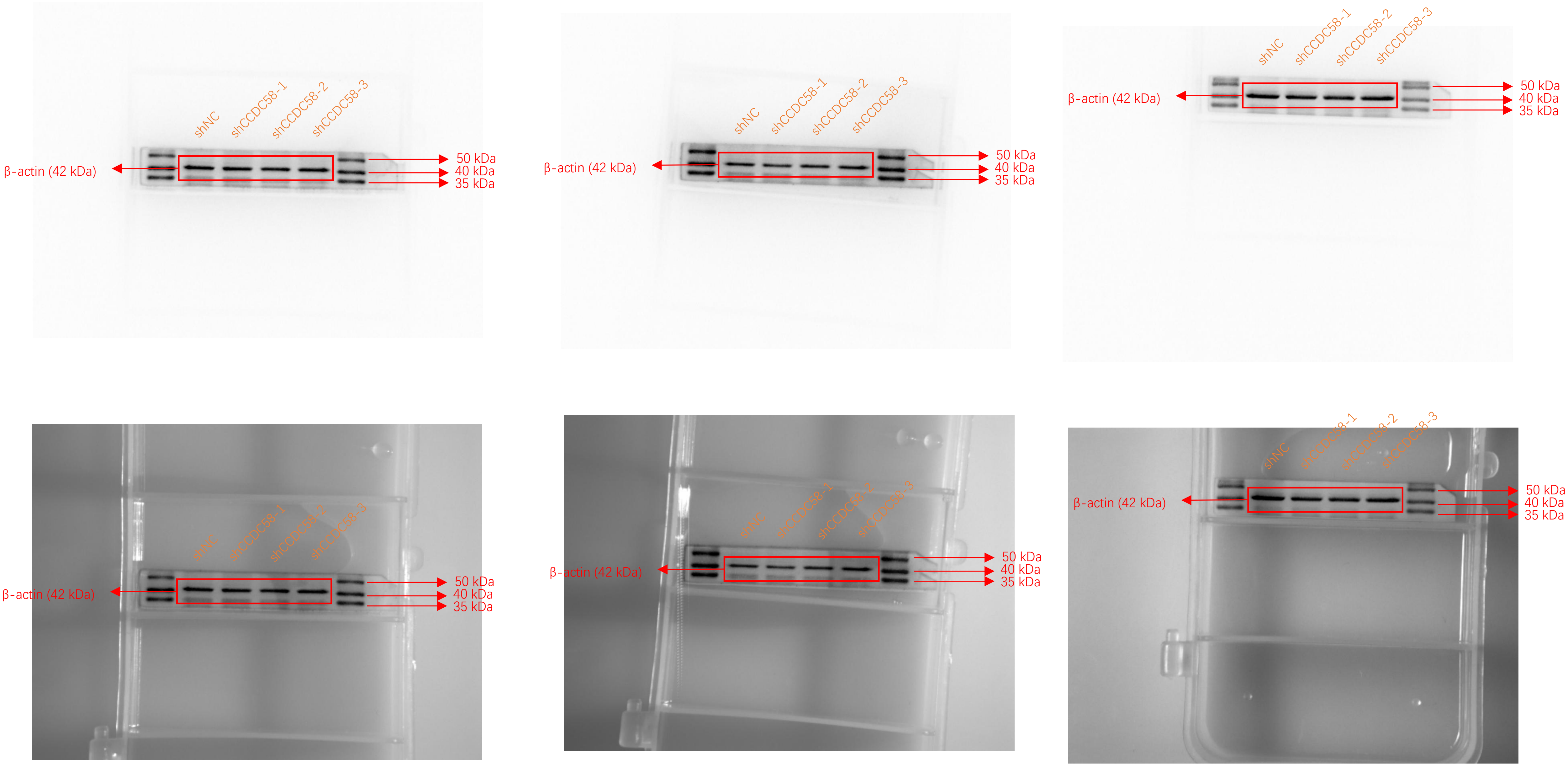

Figure 4C Knockdown efficiency of CCDC58 in H1299

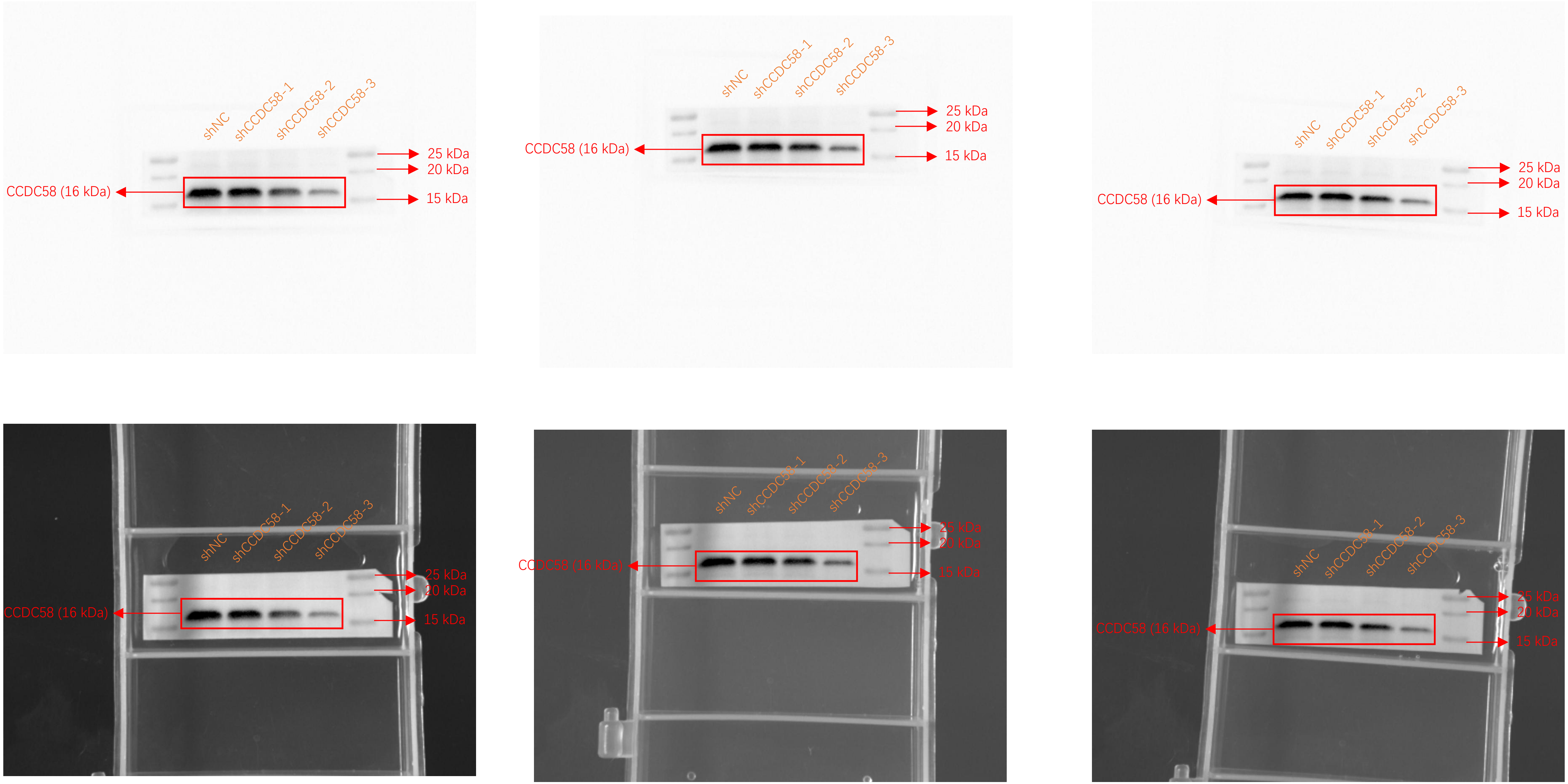

Internal Control of CCDC58

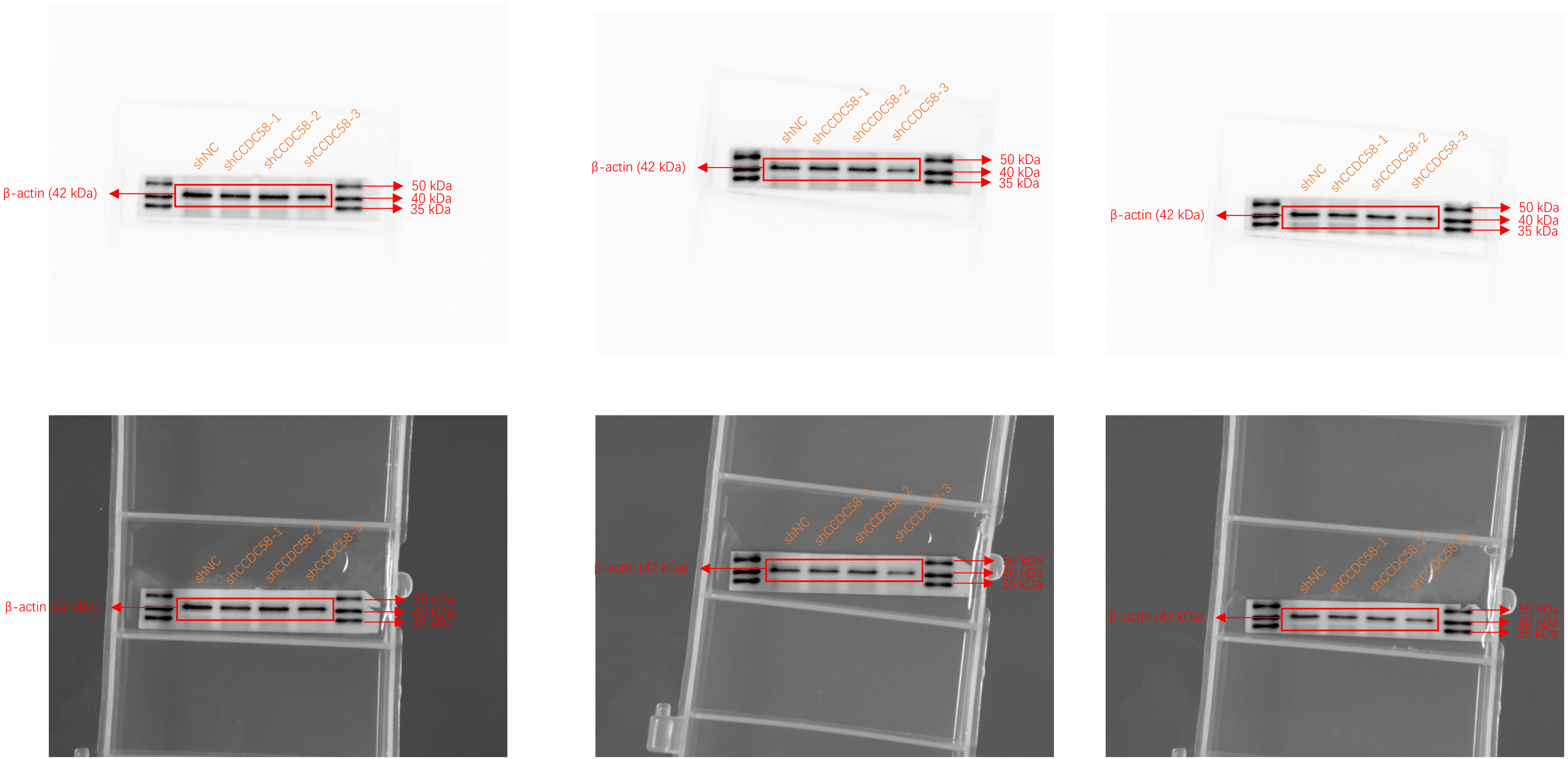

Figure 5E CCDC58 knockdown influences EMT marker protein expression

A549

E-cadherin

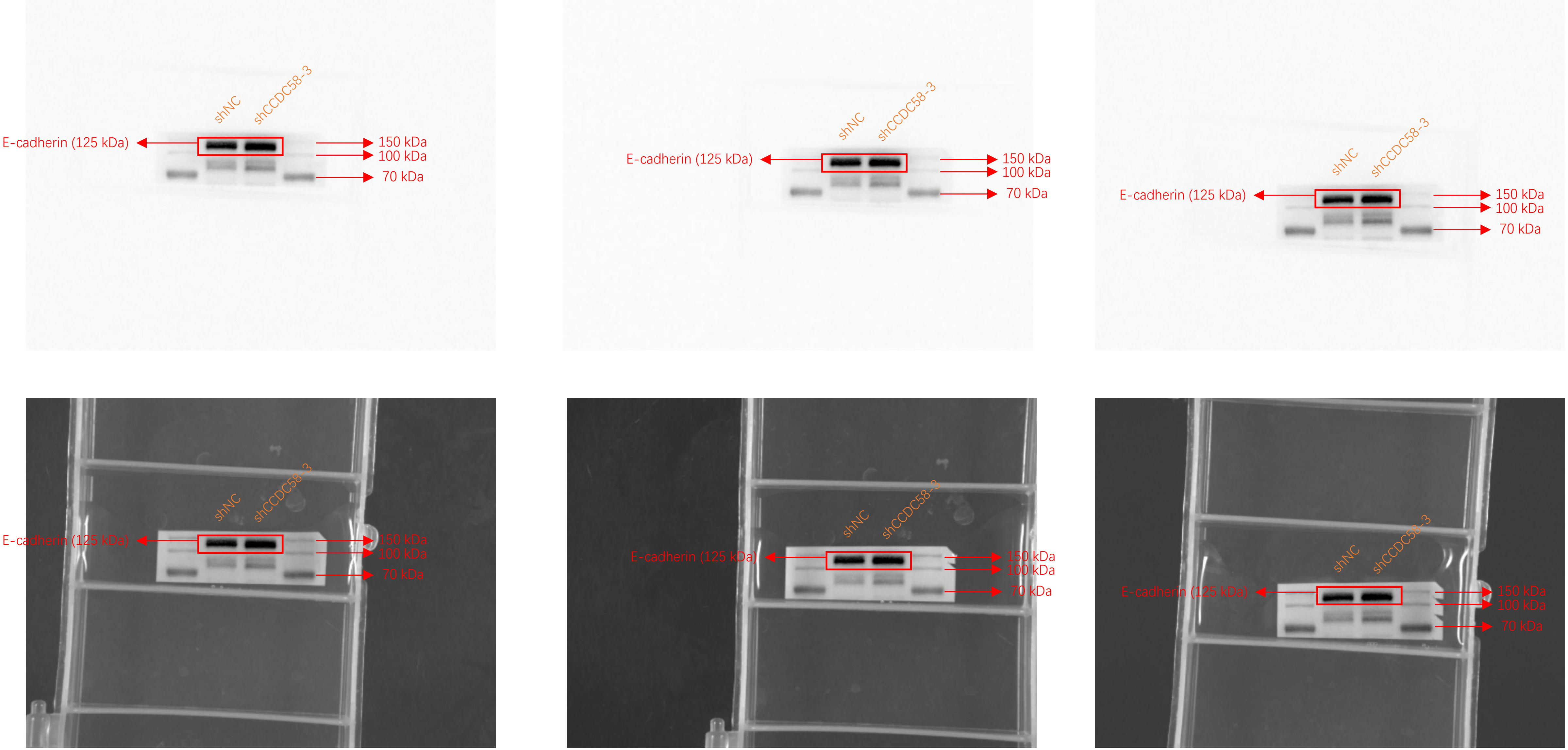

A549

Internal Control of E-cadherin

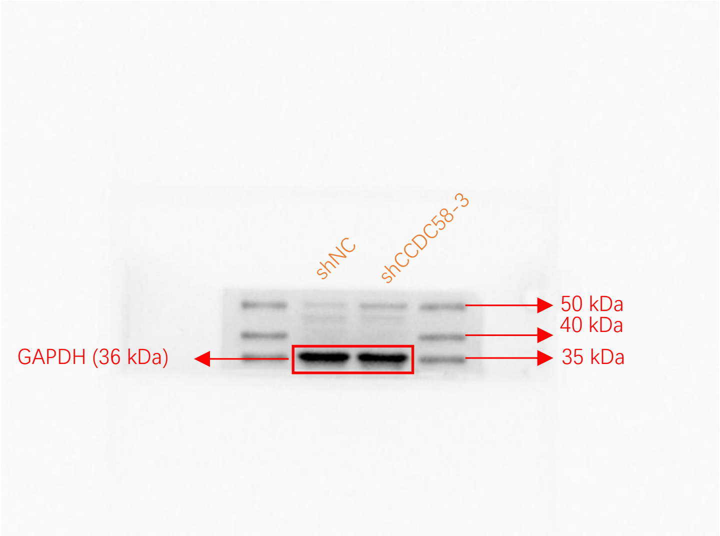

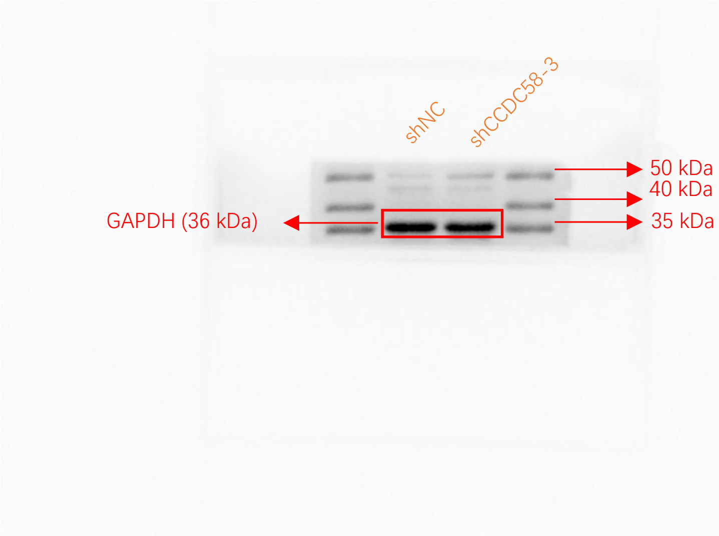

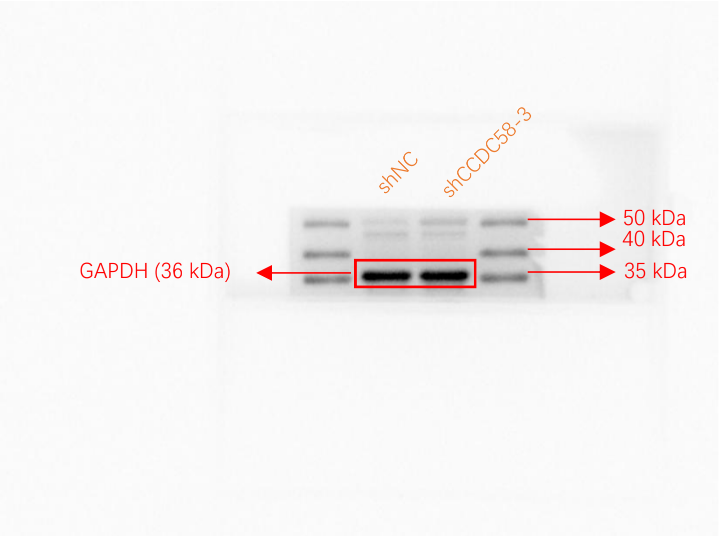

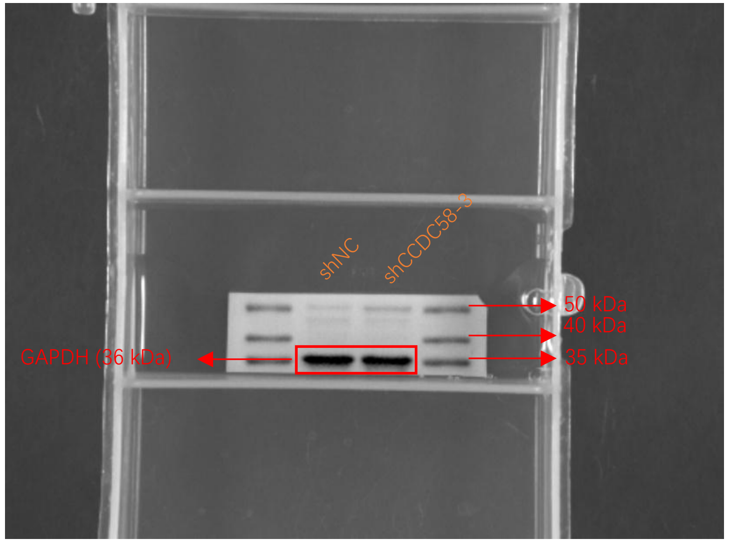

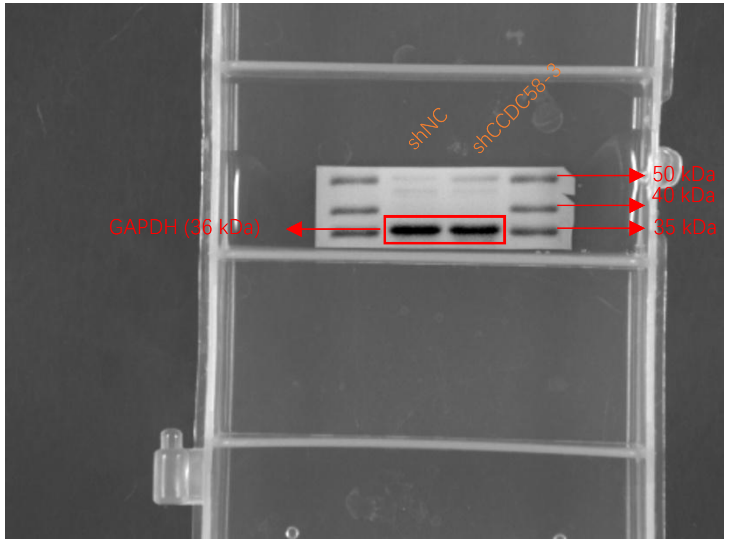

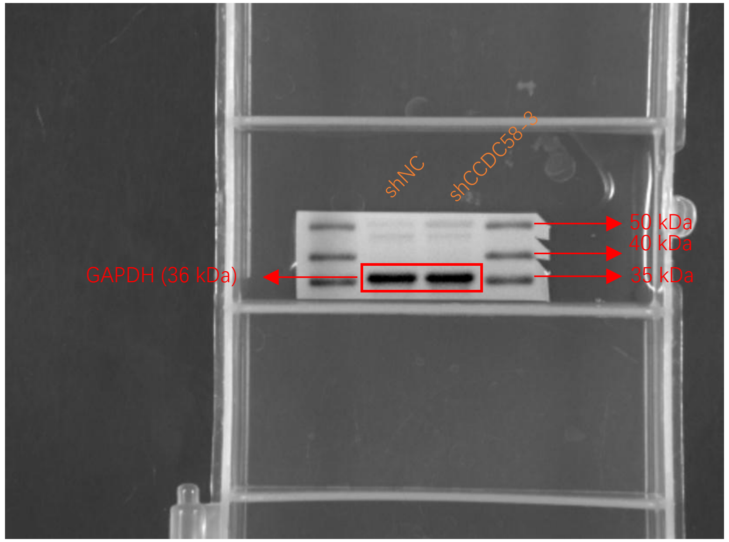

A549

N-cadherin

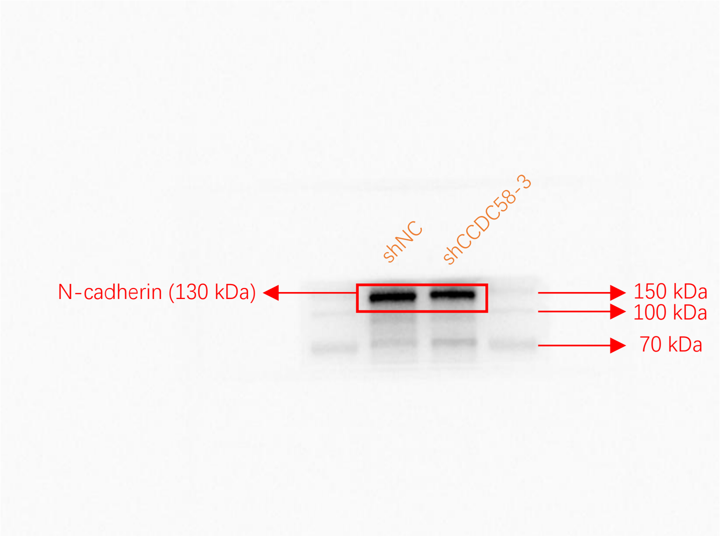

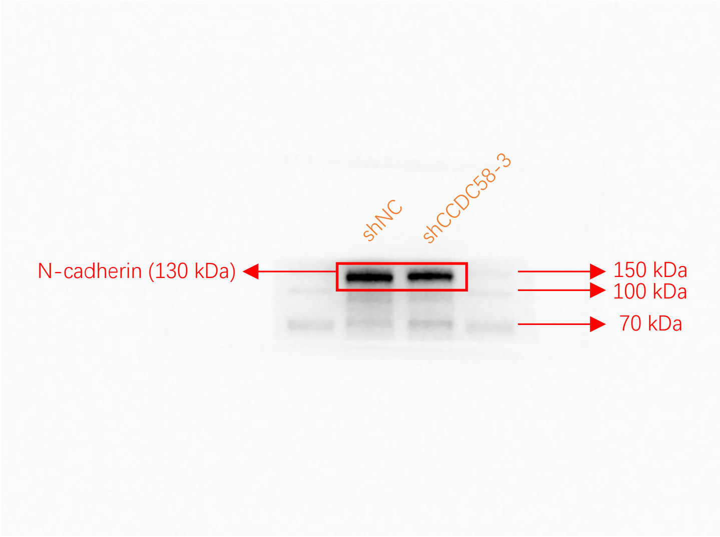

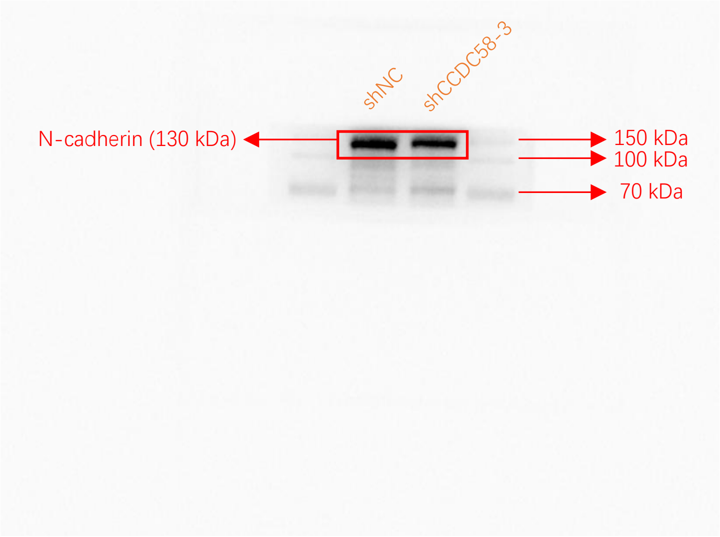

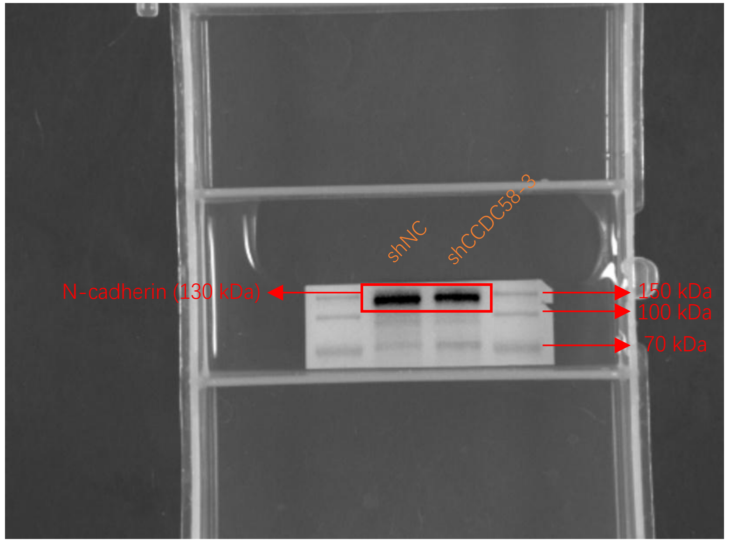

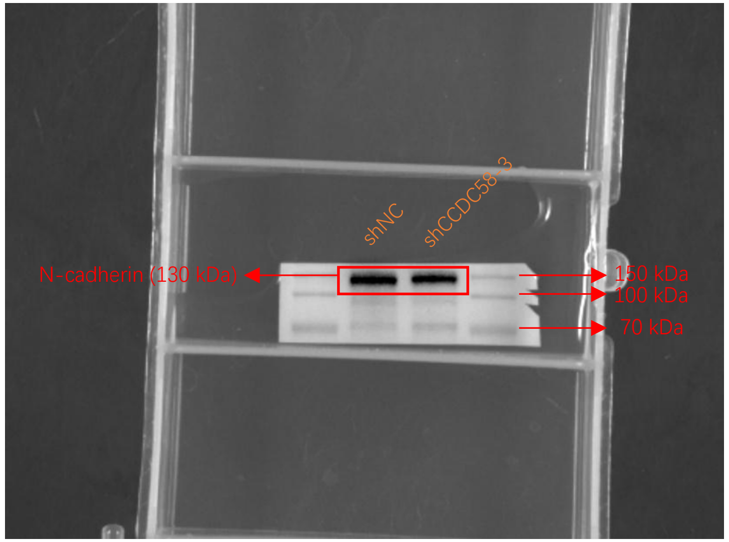

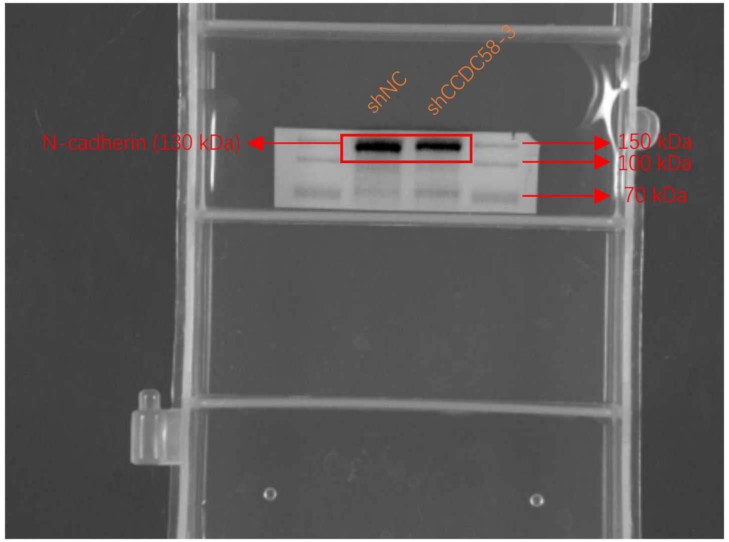

A549

Internal Control of N-cadherin

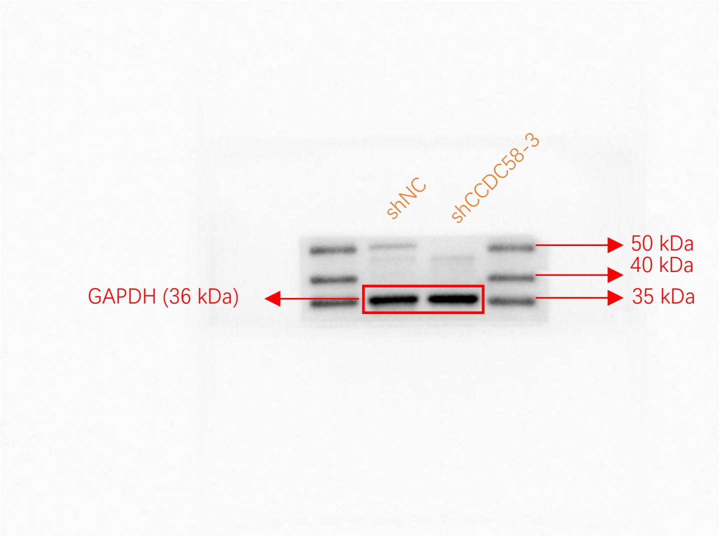

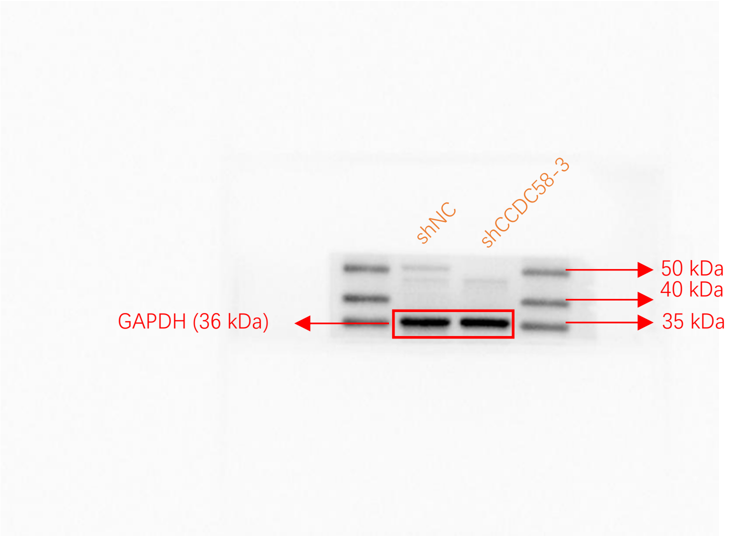

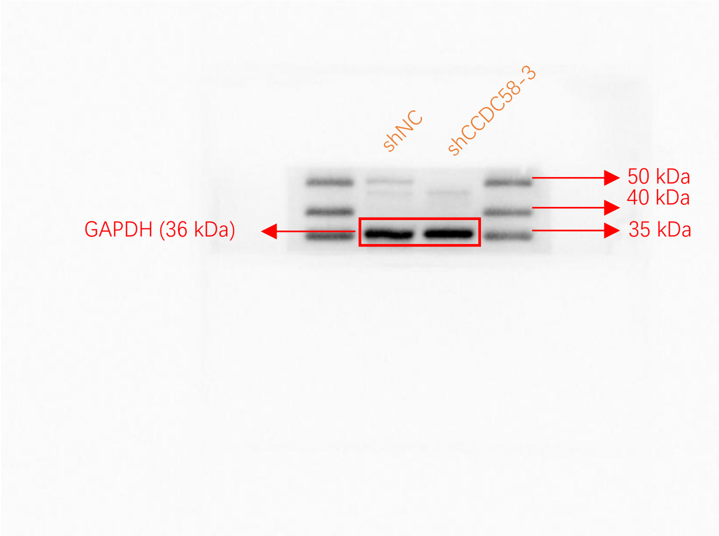

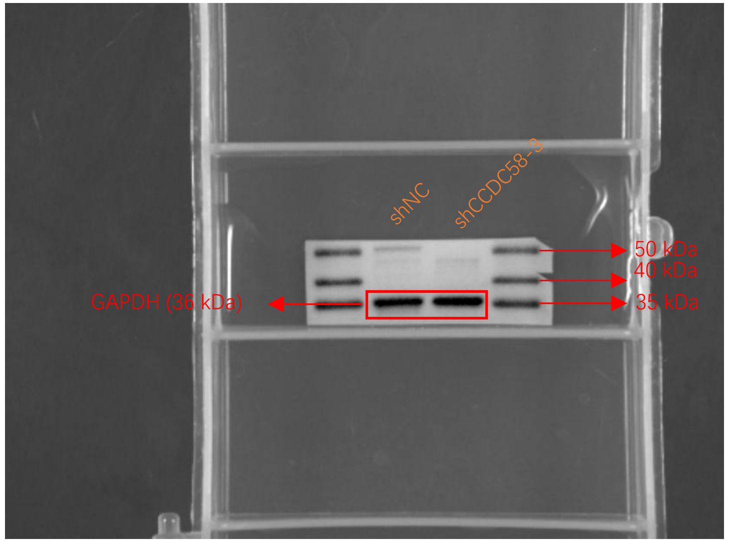

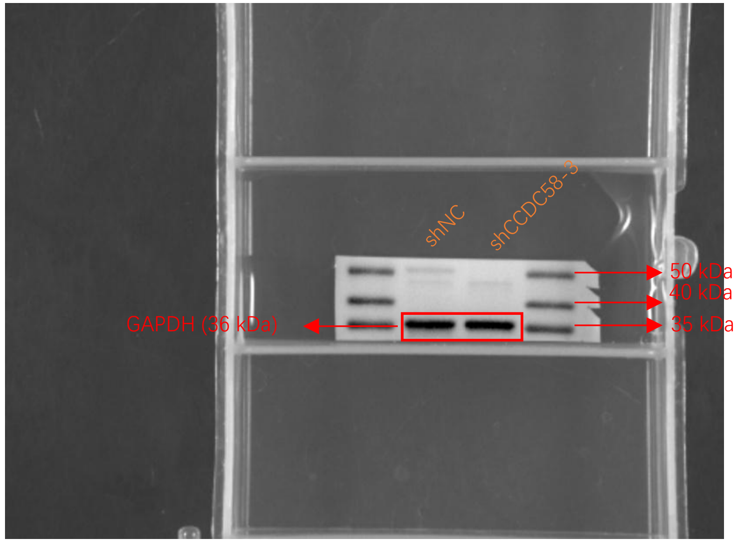

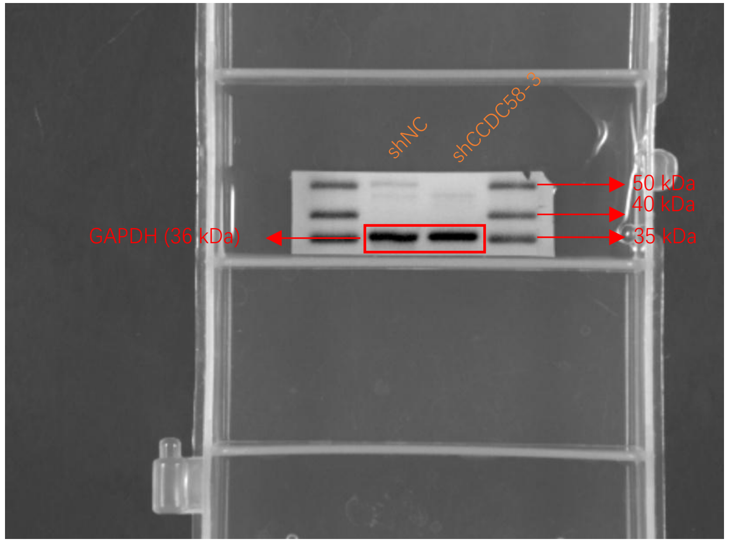

A549

Vimentin

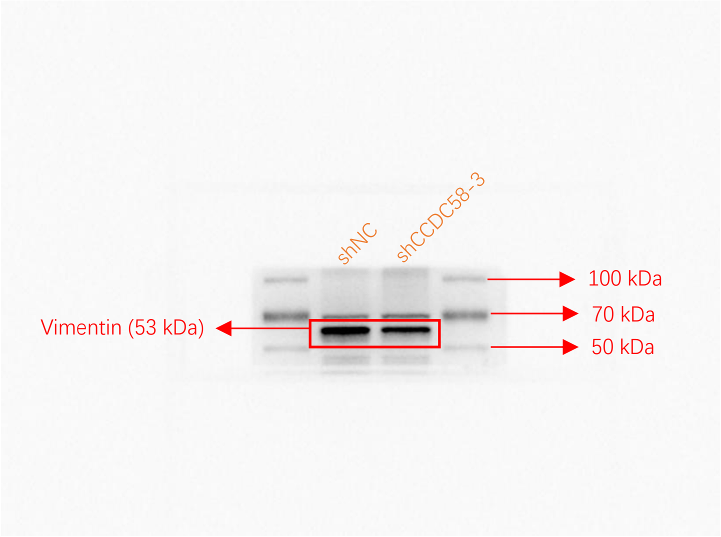

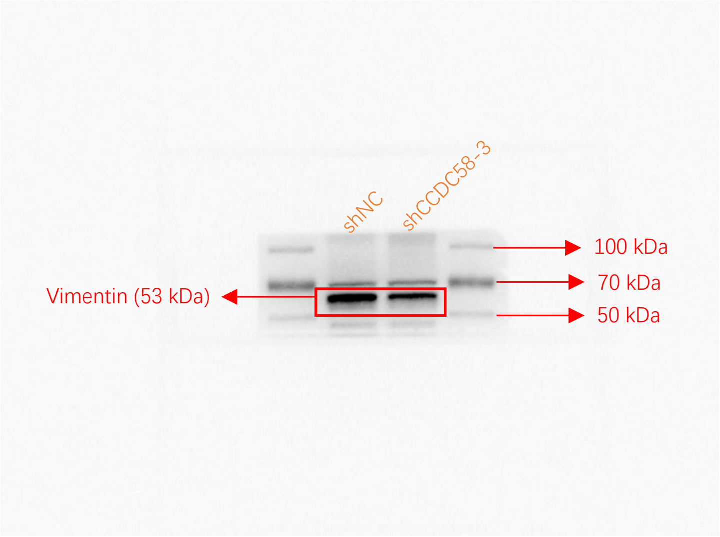

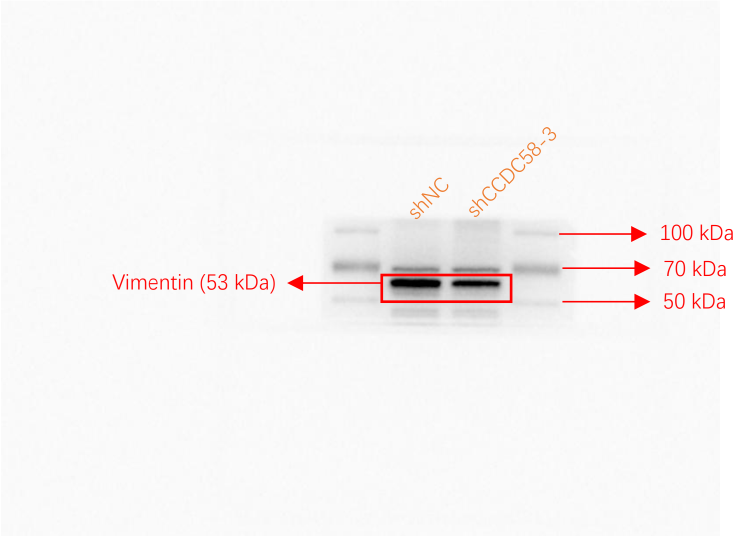

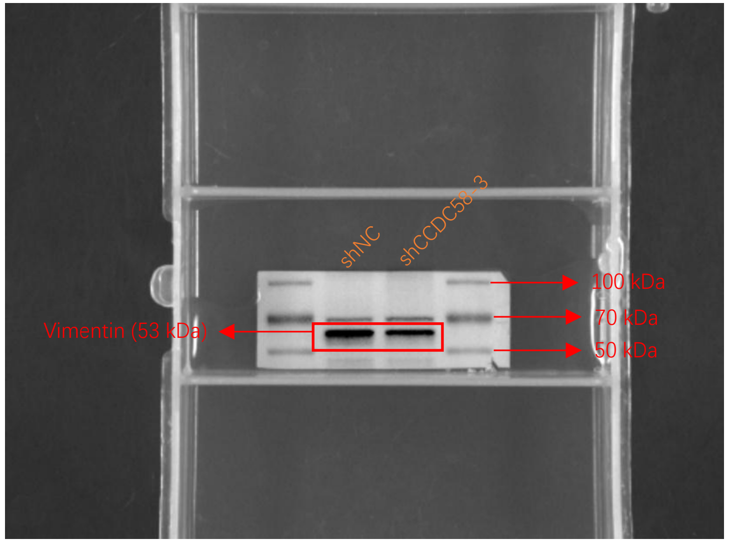

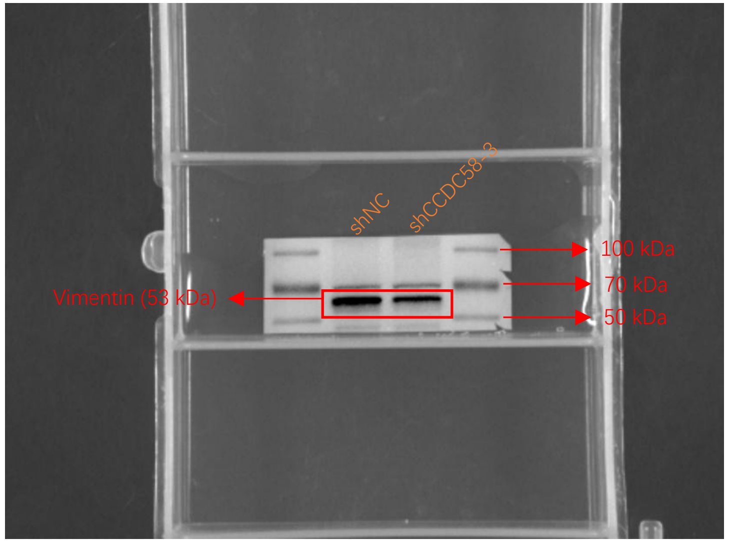

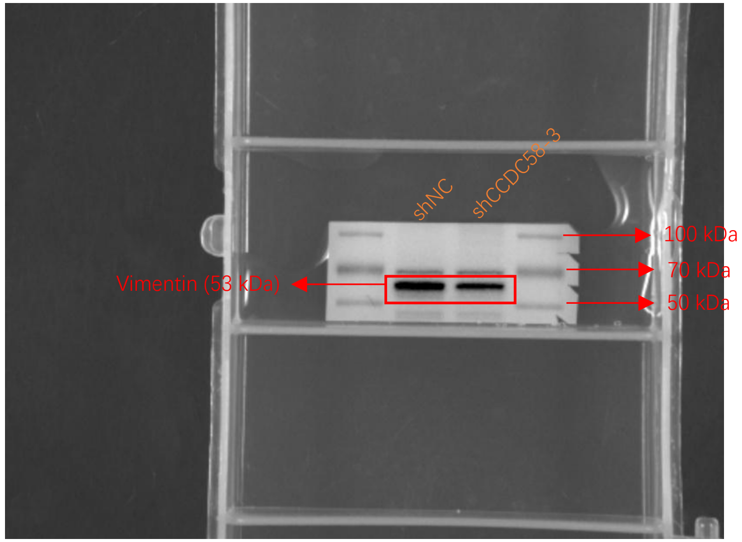

A549

Internal Control of Vimentin

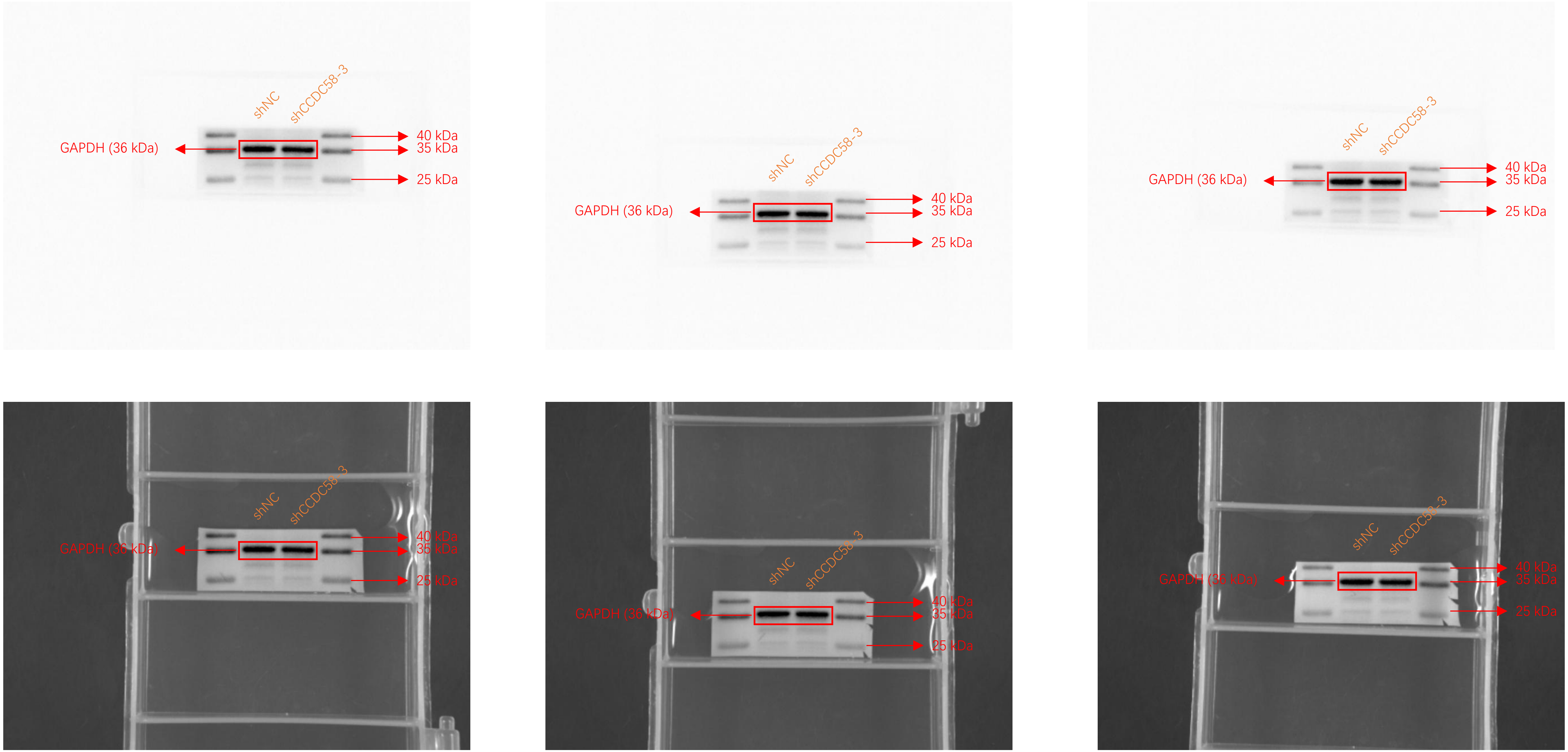

H1299

E-cadherin

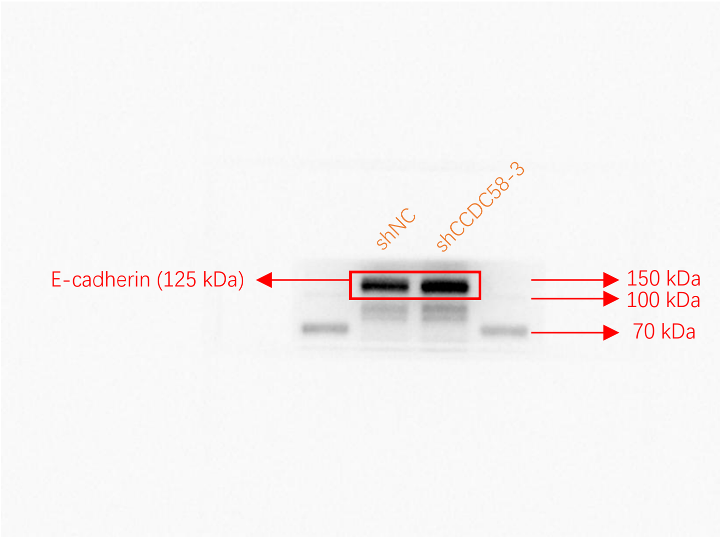

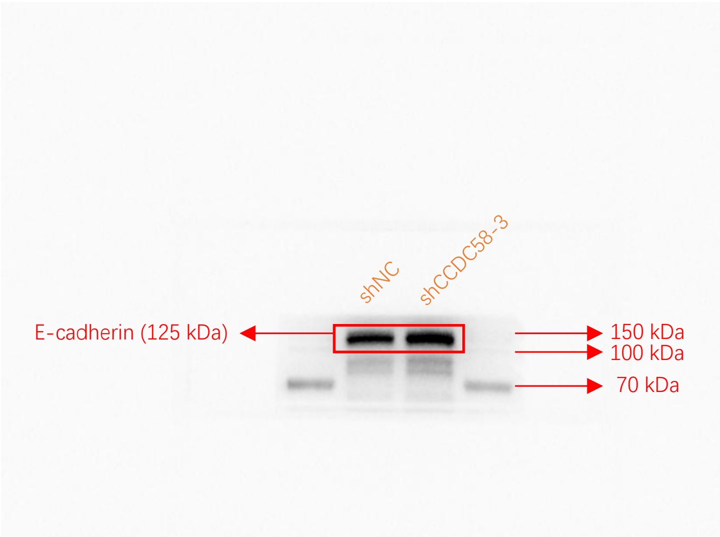

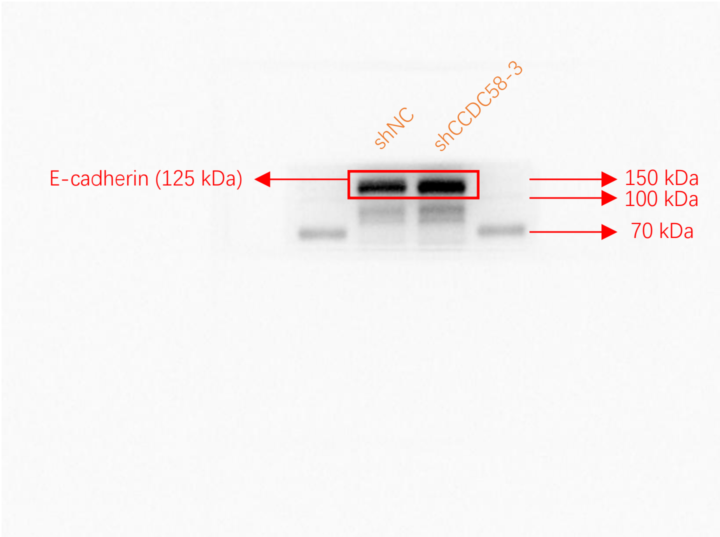

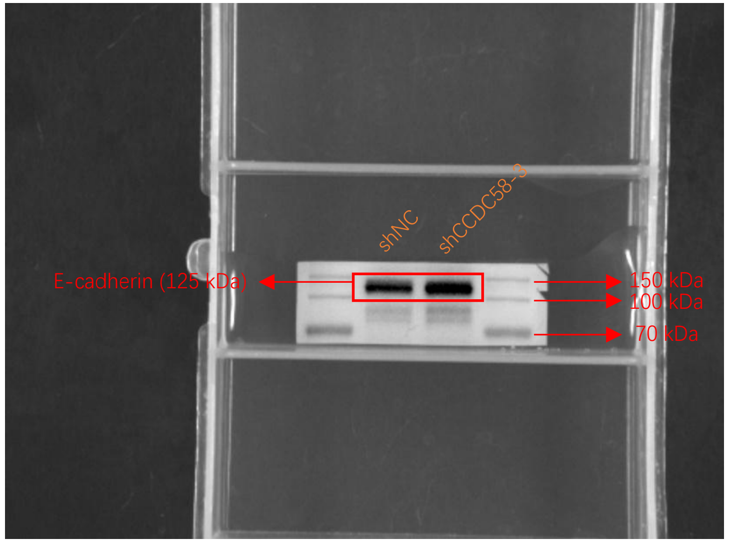

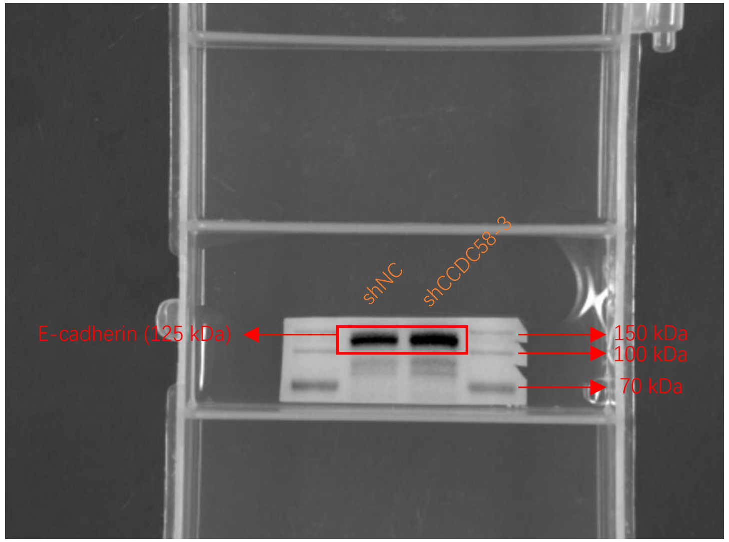

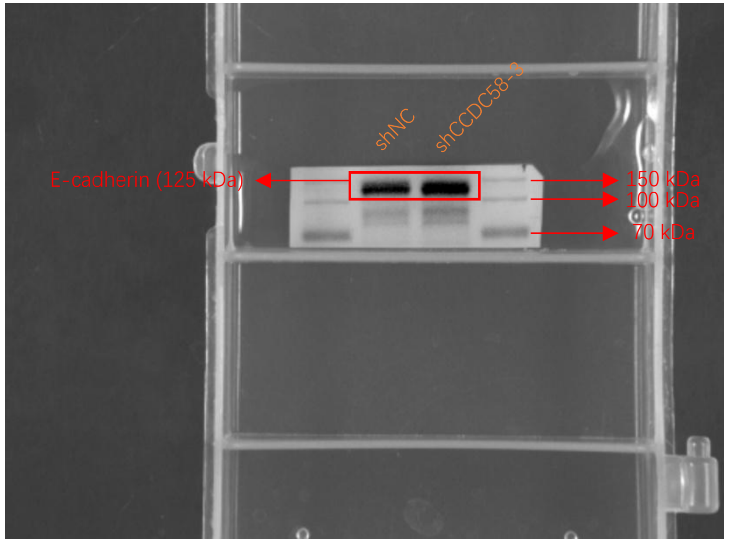

H1299

Internal Control of E-cadherin

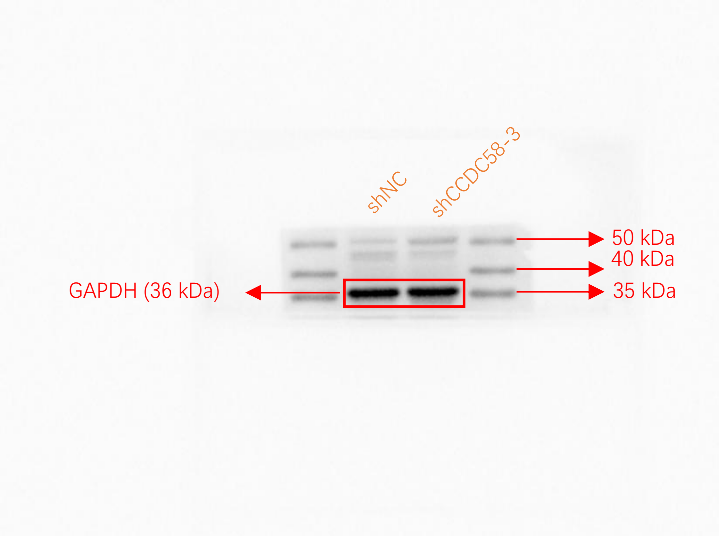

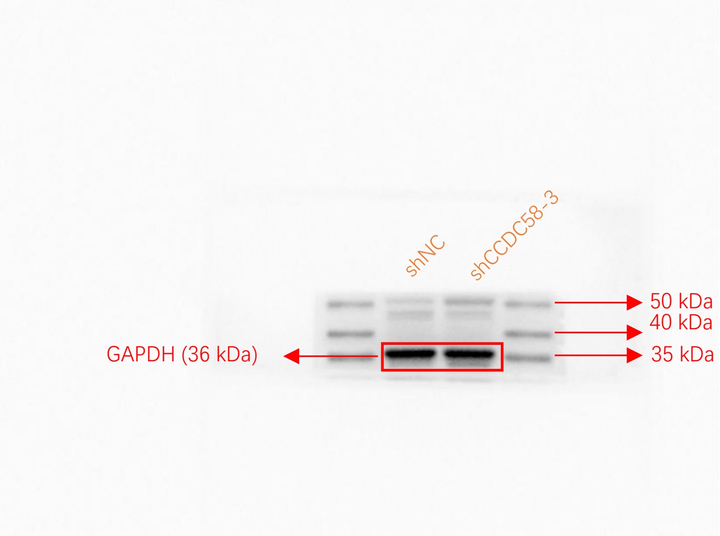

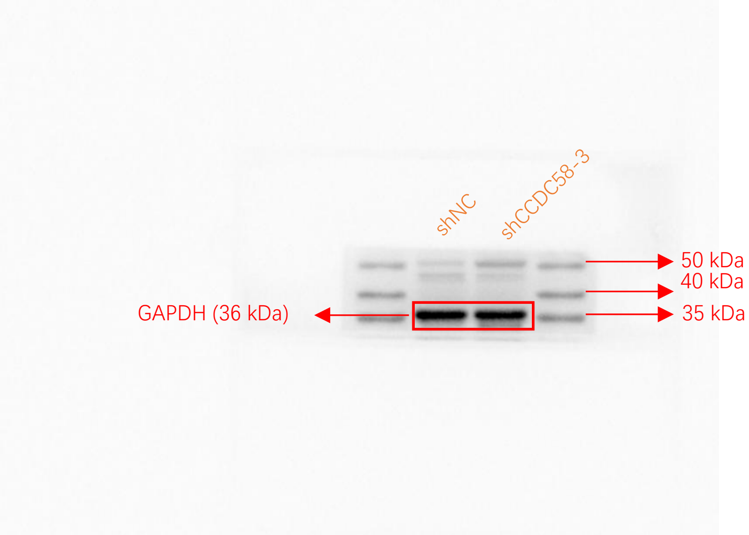

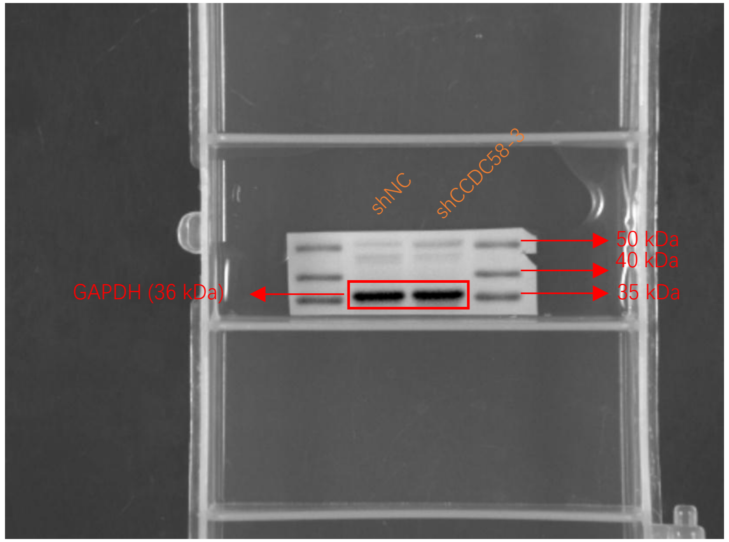

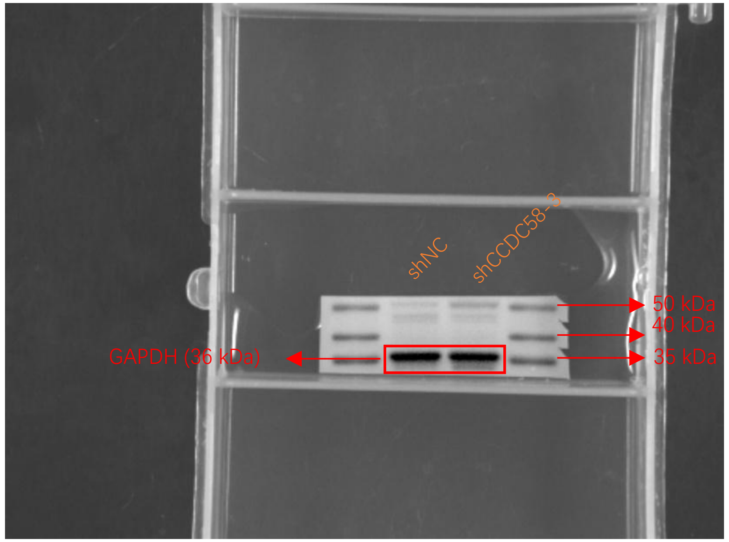

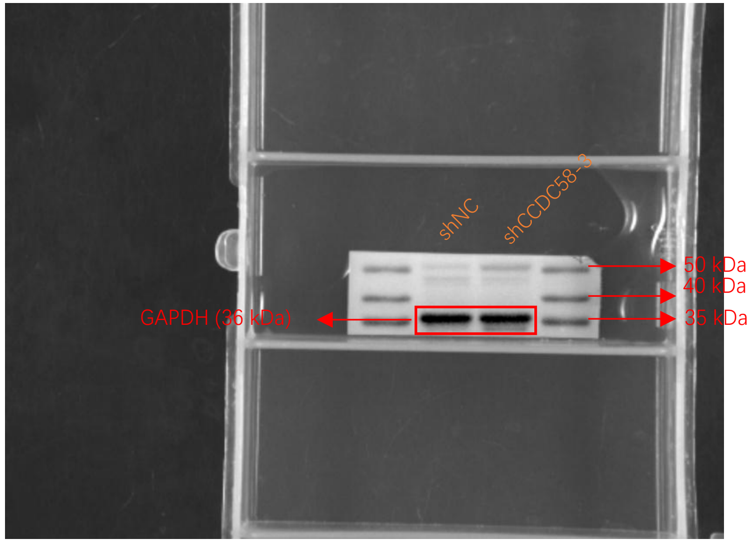

H1299

N-cadherin

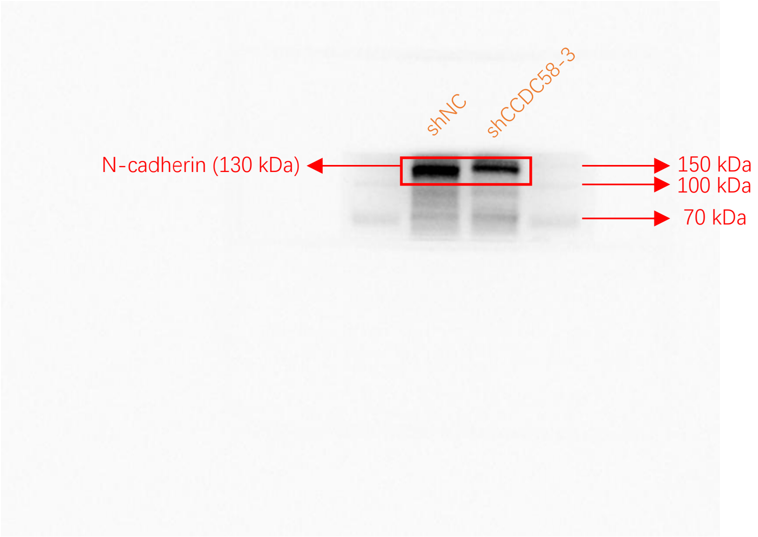

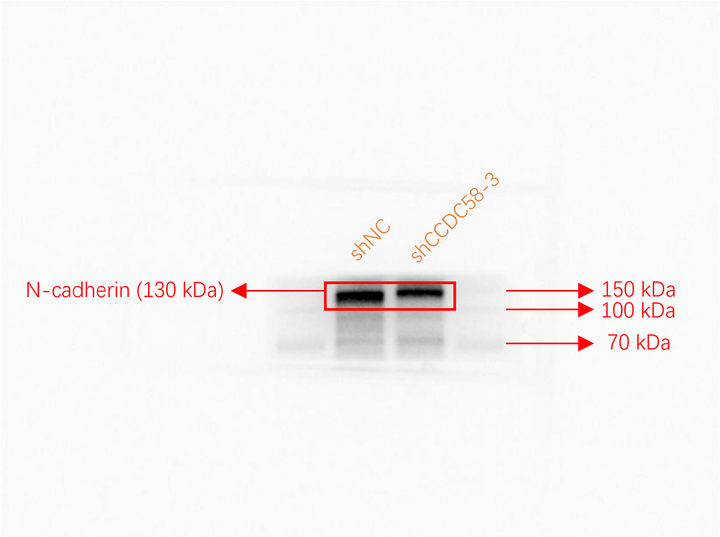

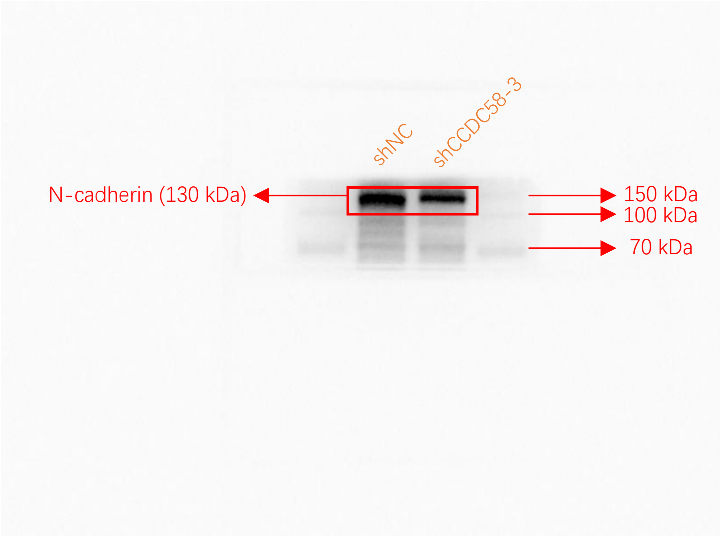

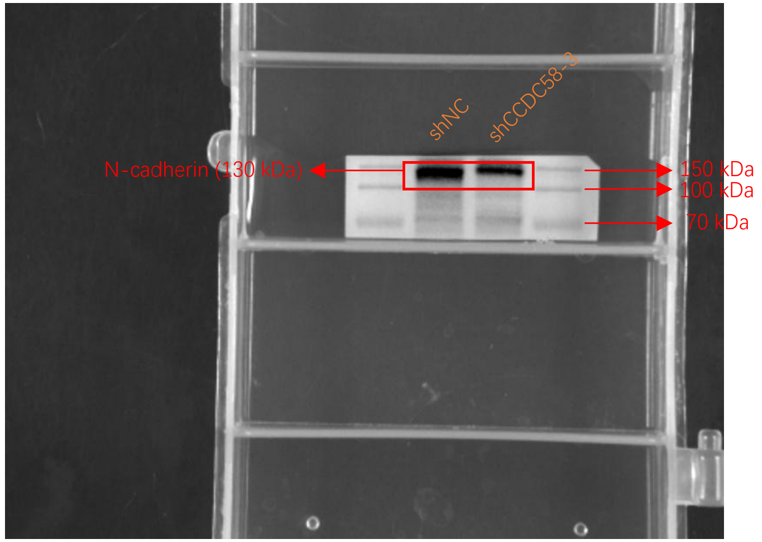

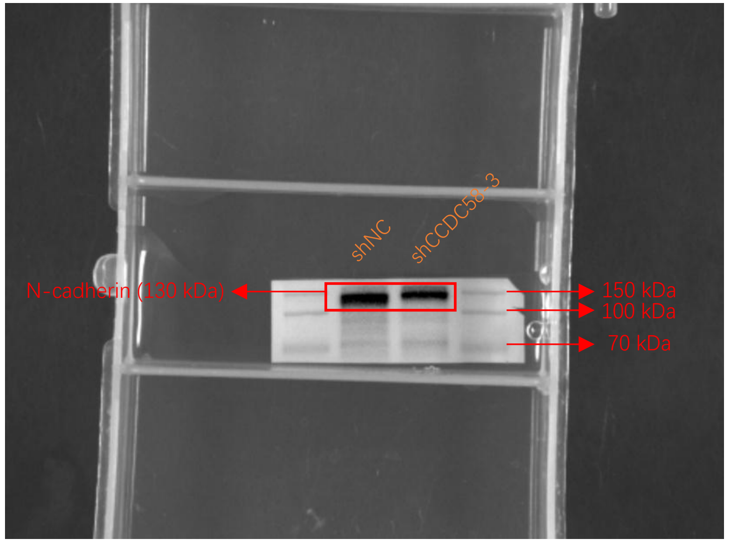

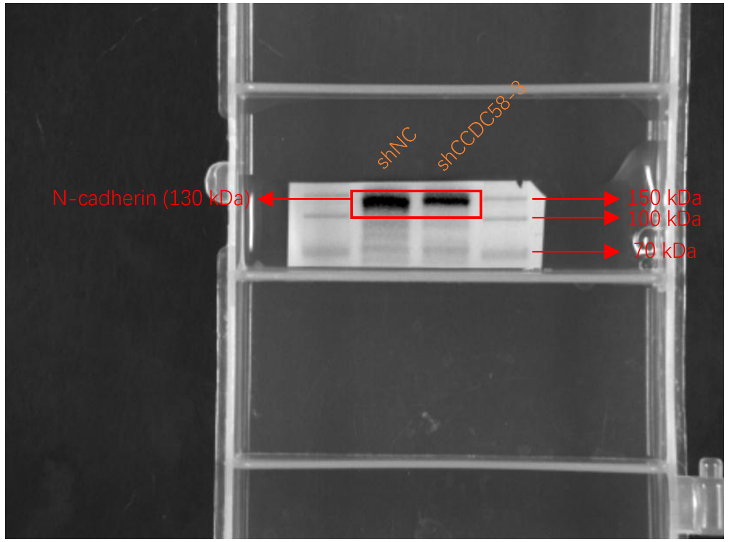

H1299

Internal Control of N-cadherin

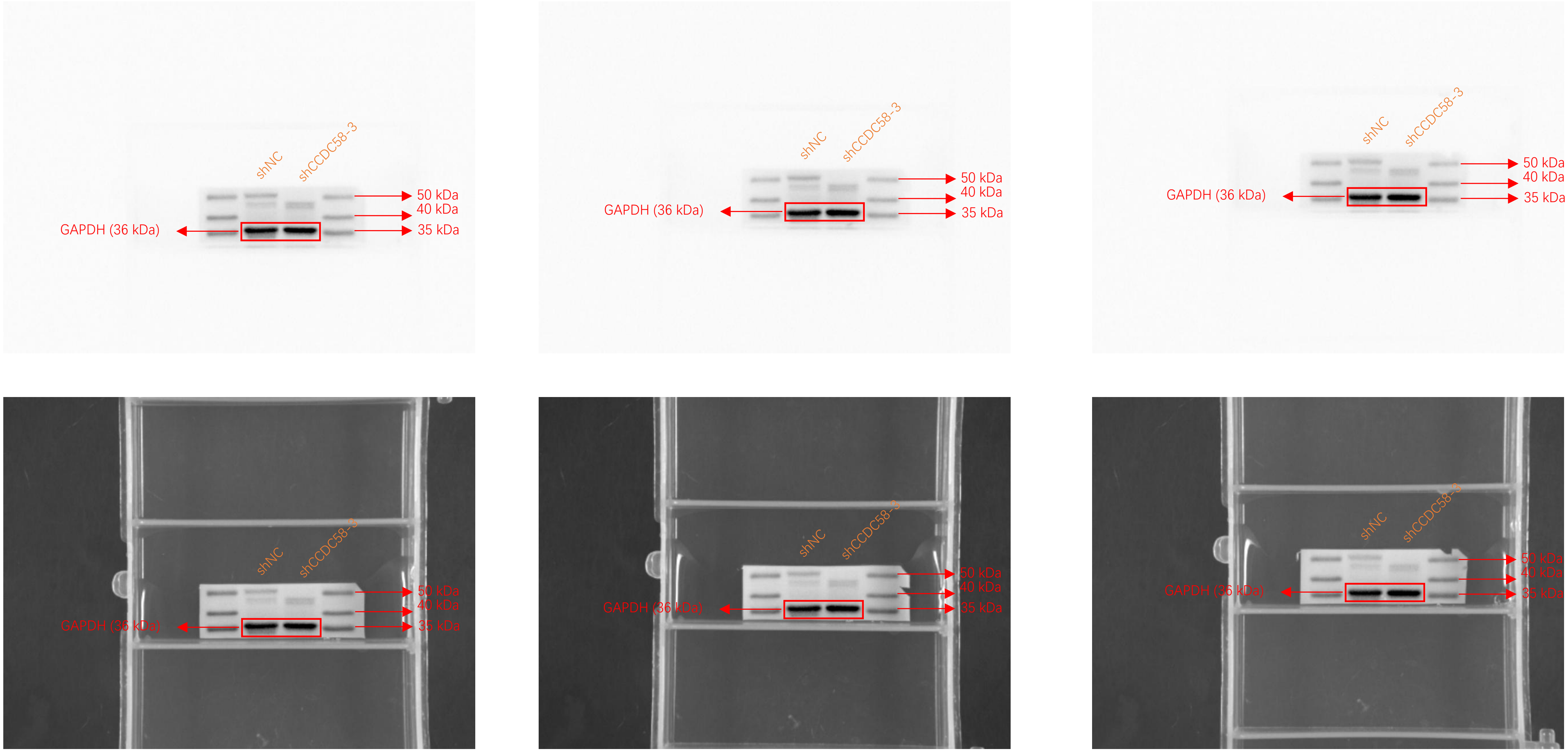

H1299

Vimentin

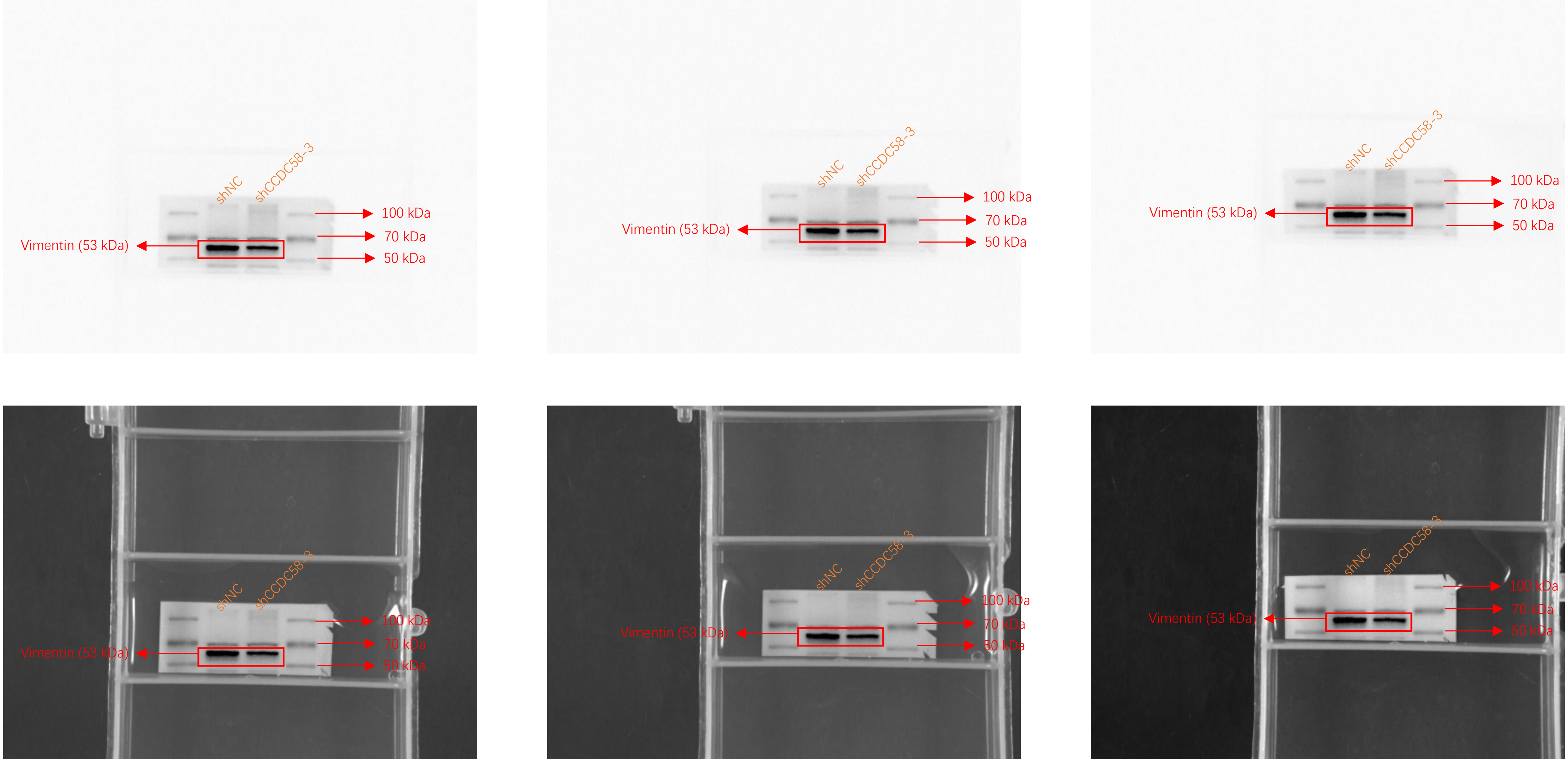

H1299

Internal Control of Vimentin

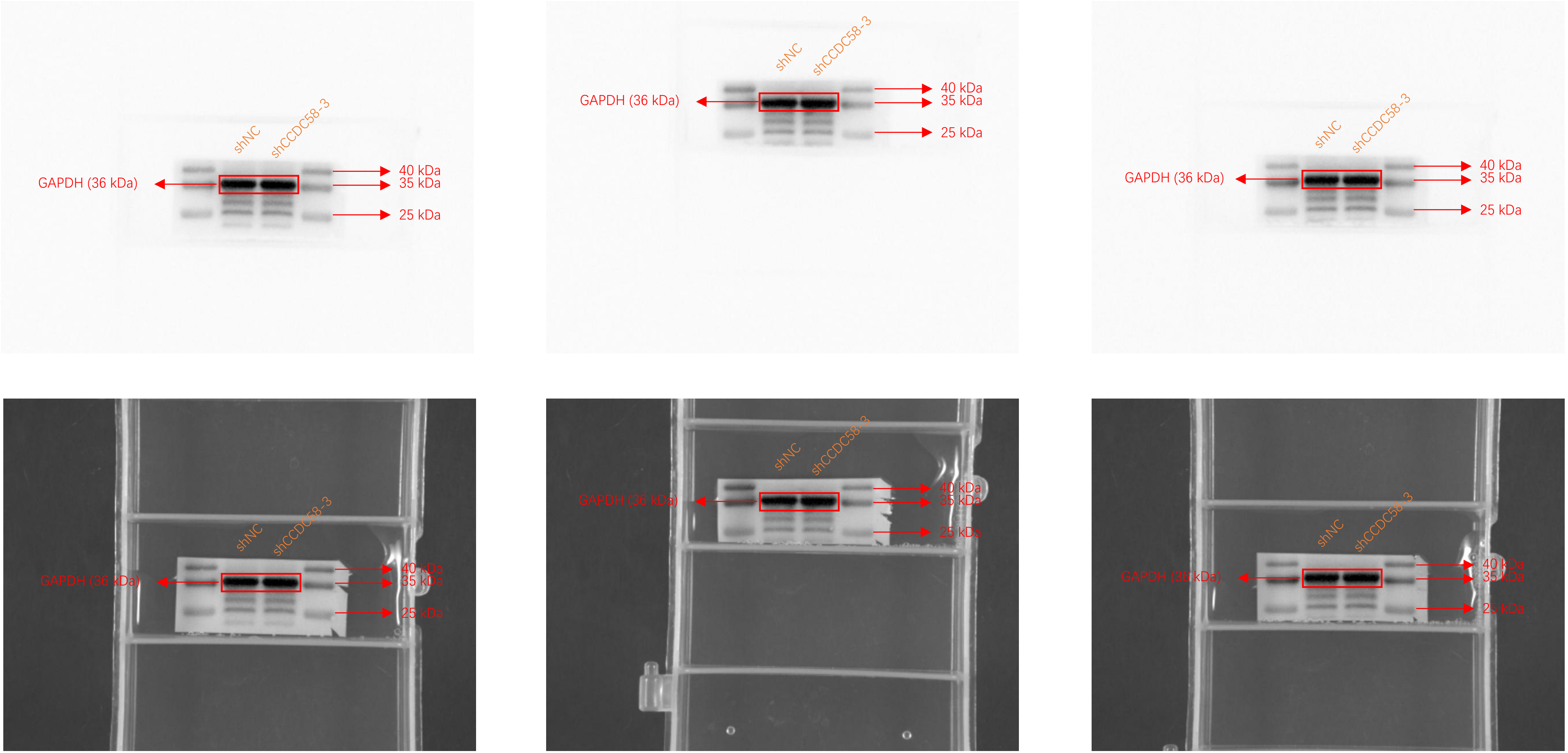

Figure 6E CCDC58 knockdown influences apoptosis marker protein expression

A549

BAX

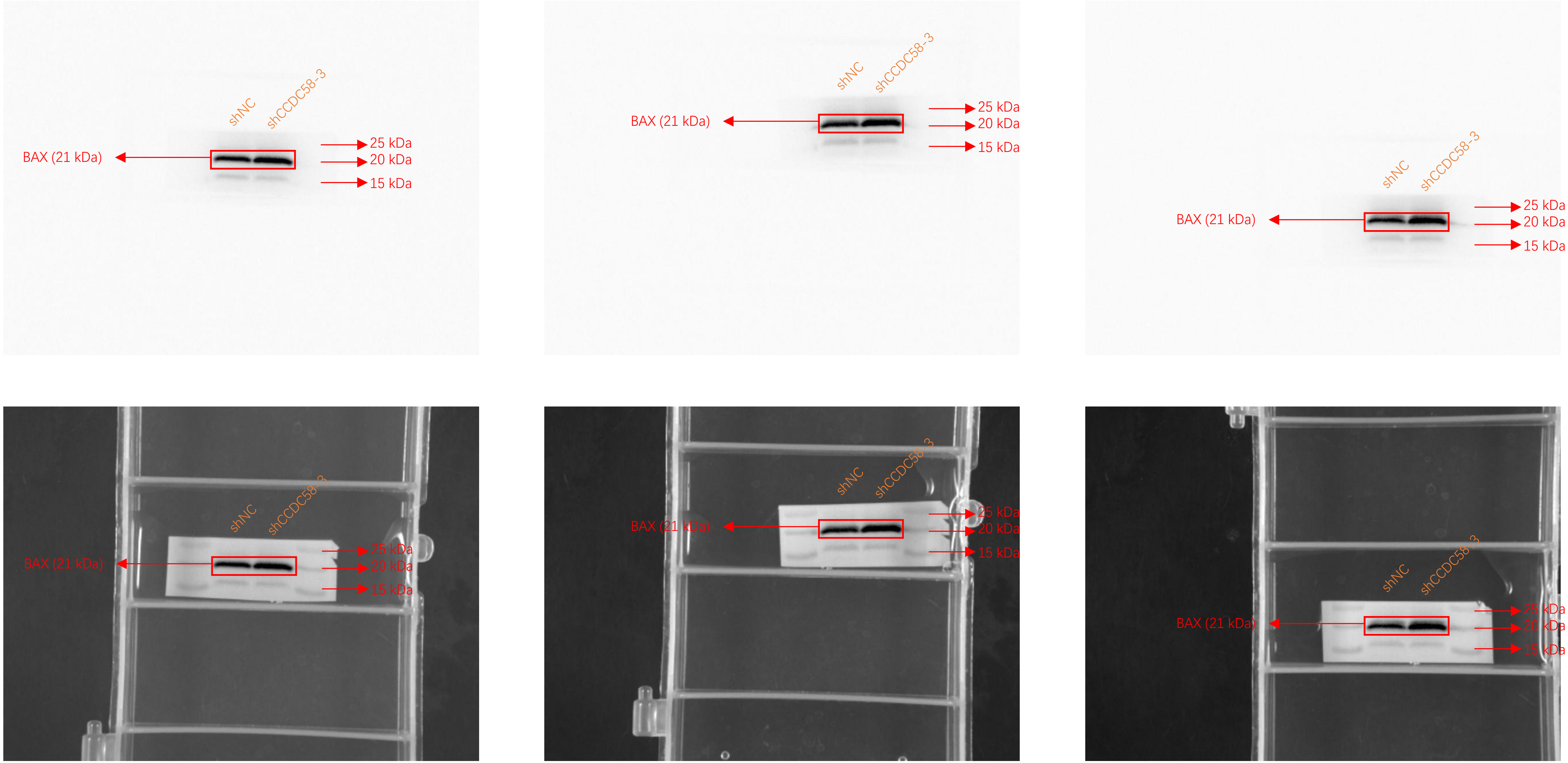

A549

Internal Control of BAX

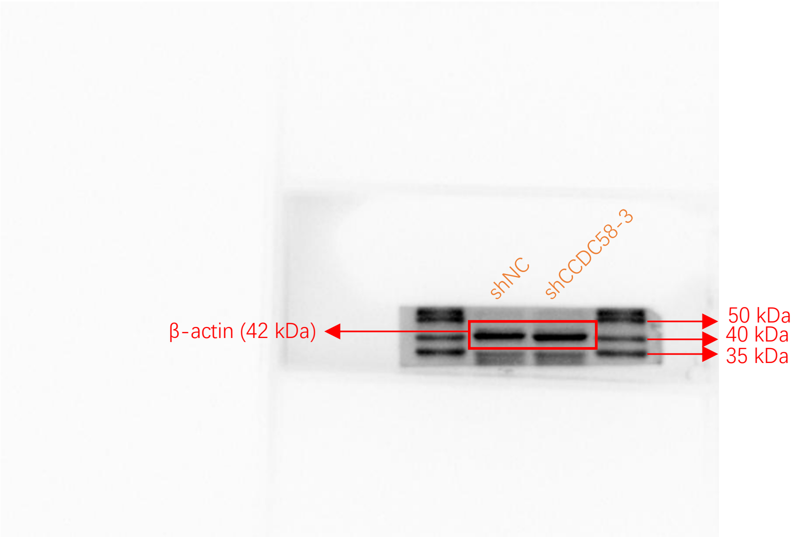

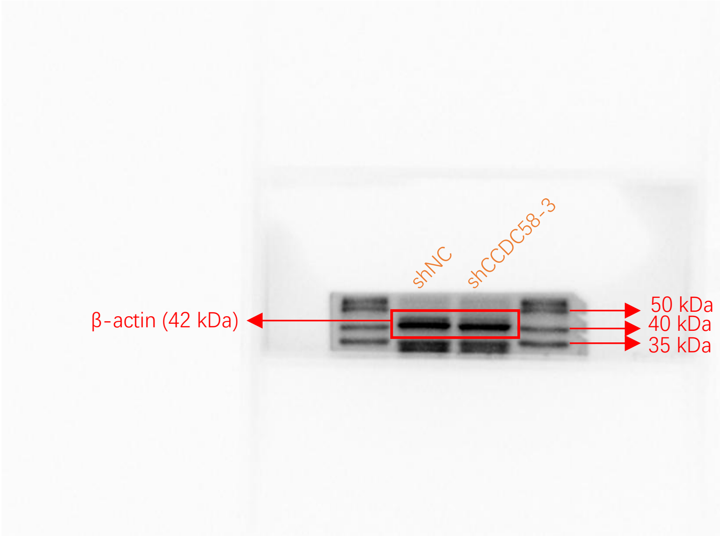

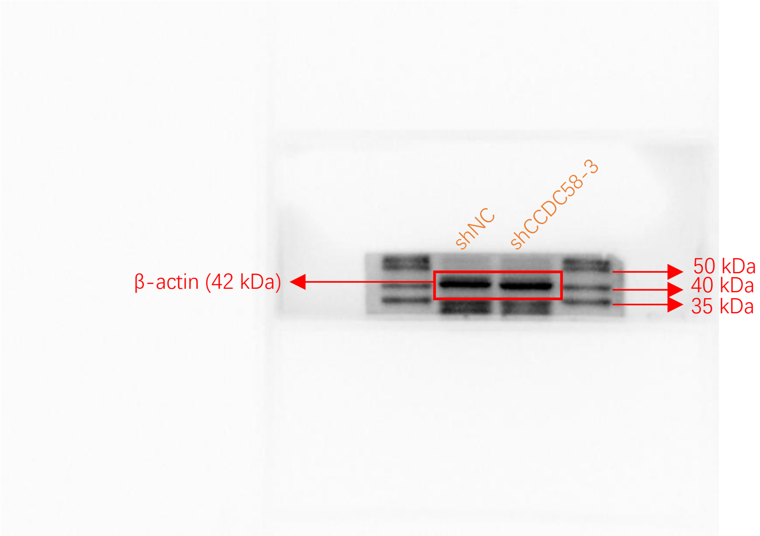

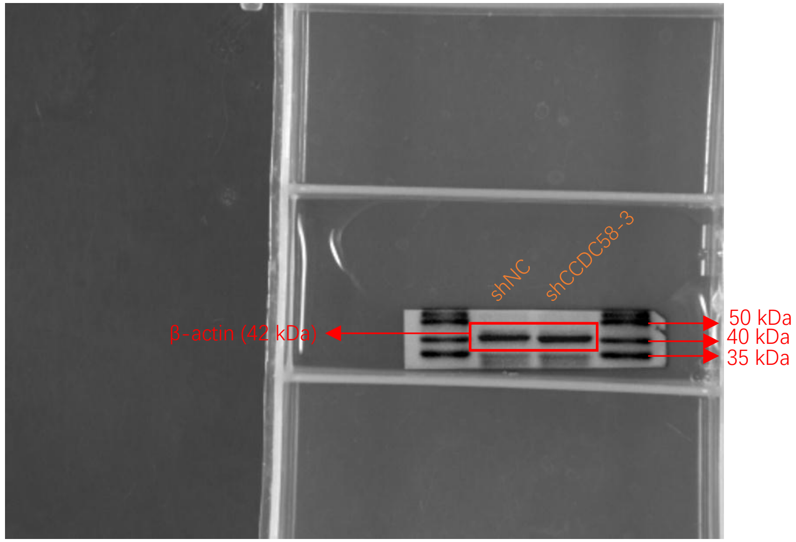

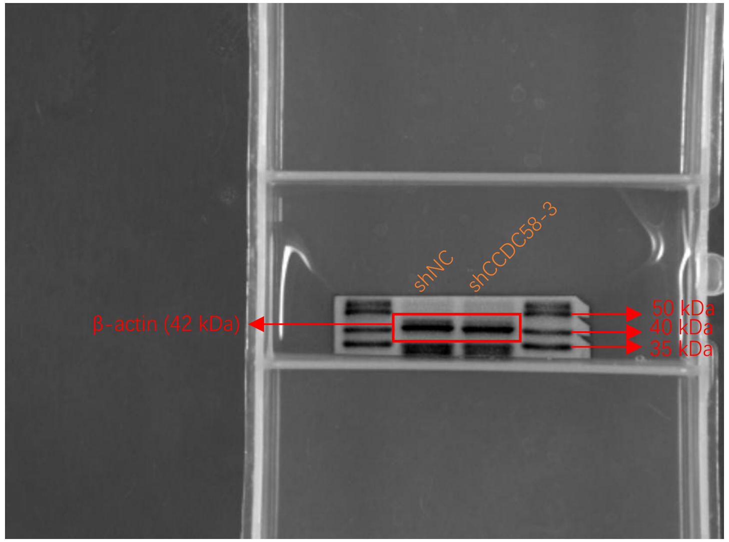

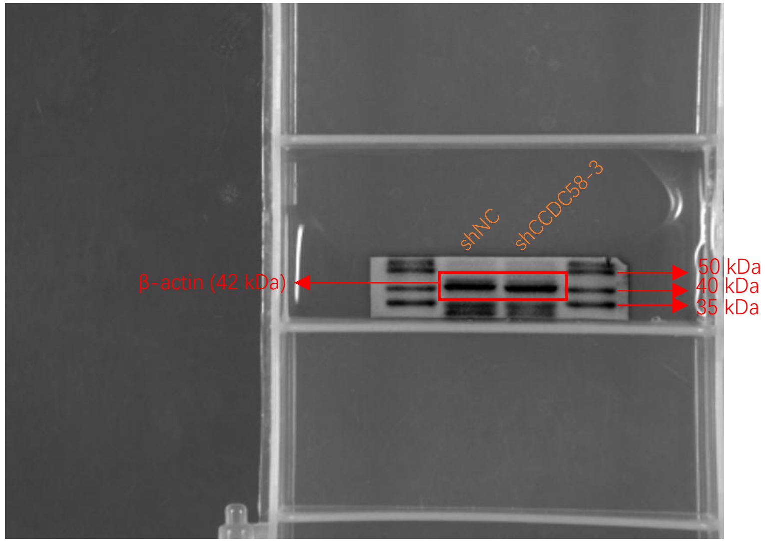

A549

BCL-2

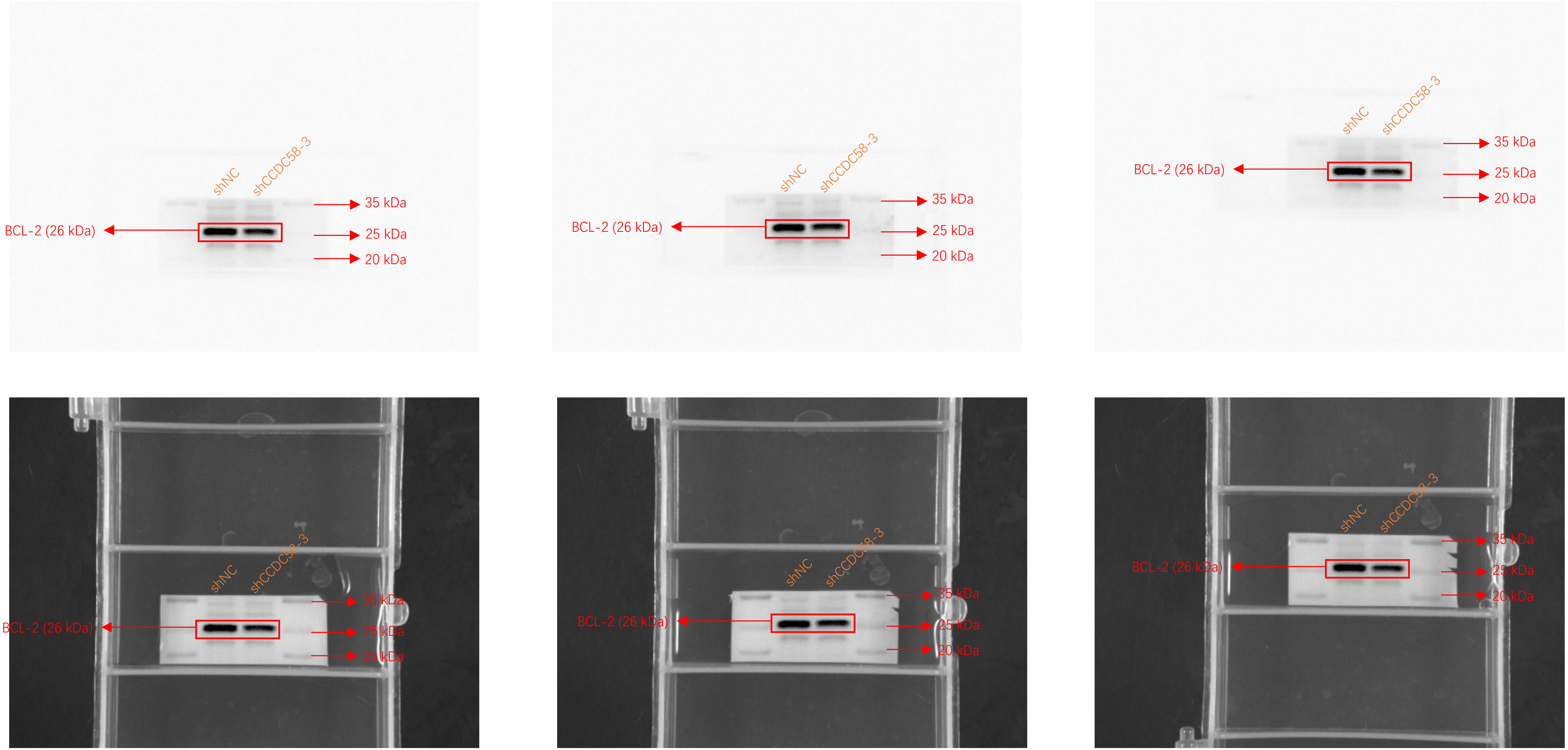

A549

Internal Control of BCL-2

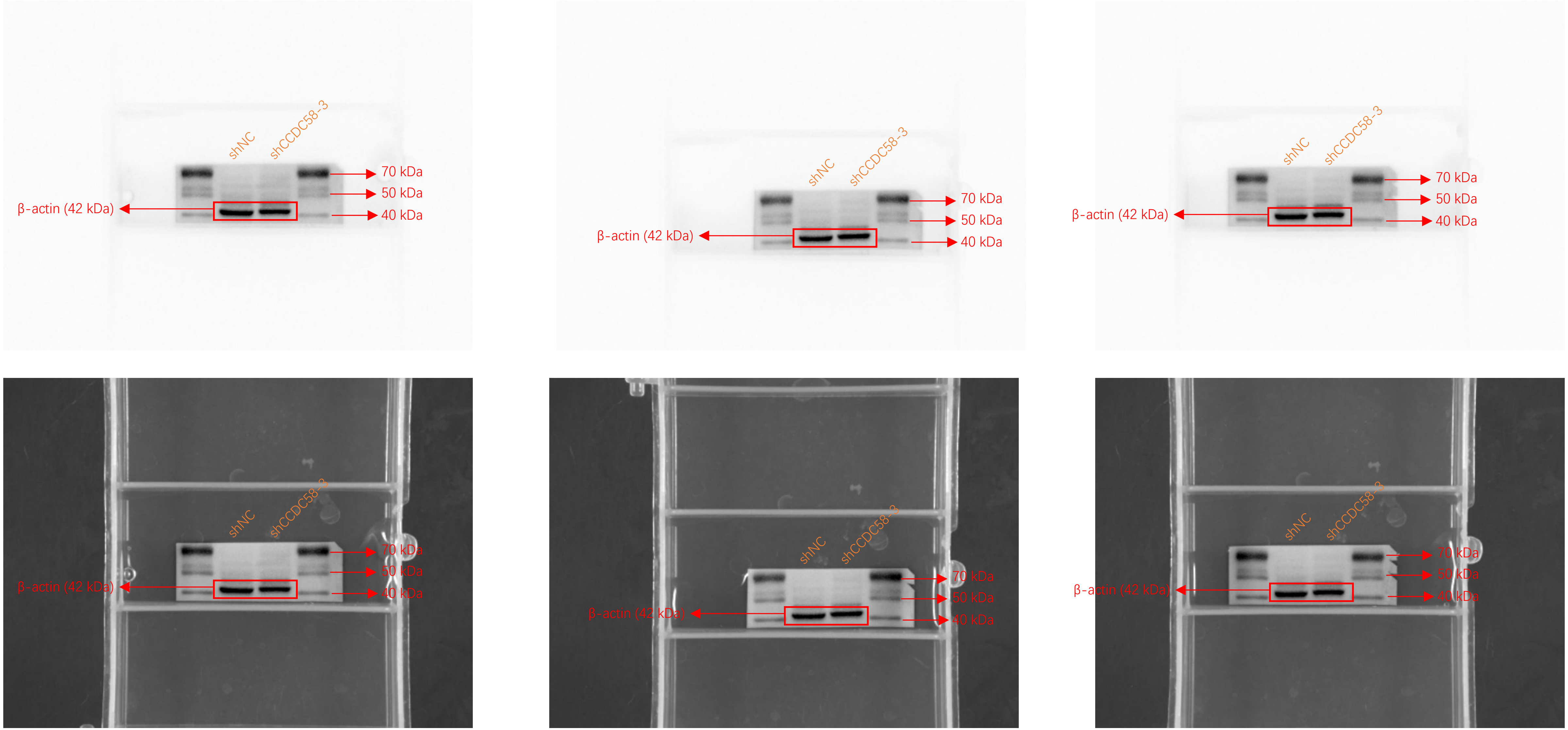

H1299

BAX

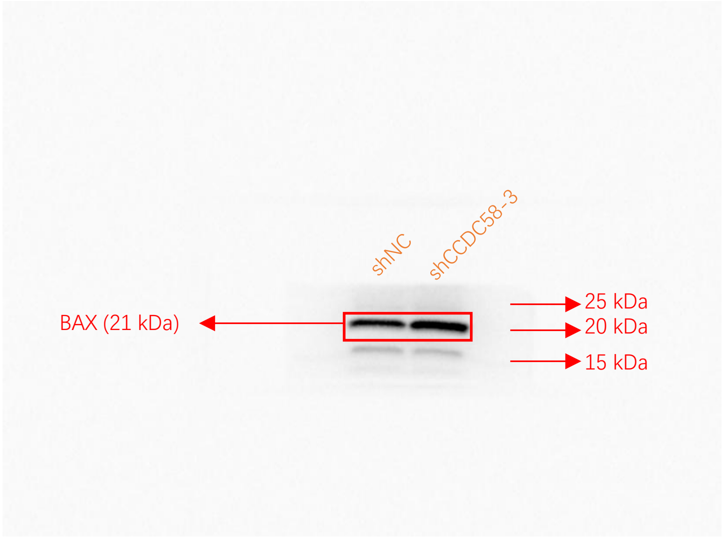

shNC
shCCDC58-3
BAX (21 kDa)
25 kDa
20 kDa
15 kDa

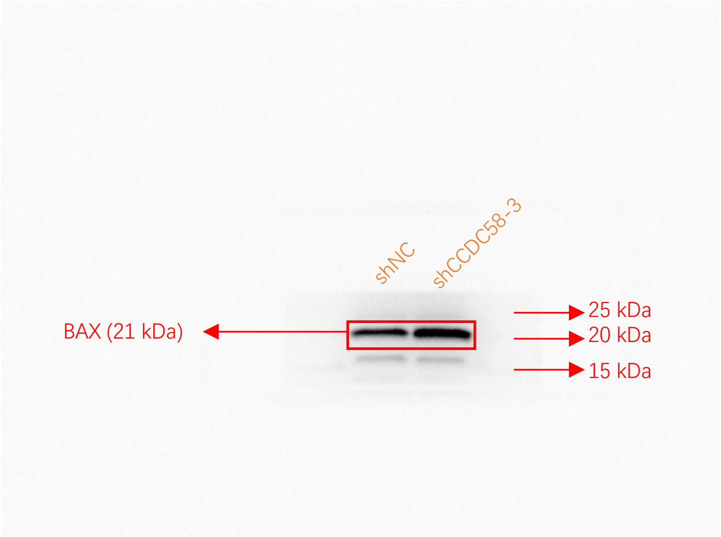

shNC
shCCDC58-3
BAX (21 kDa)
25 kDa
20 kDa
15 kDa

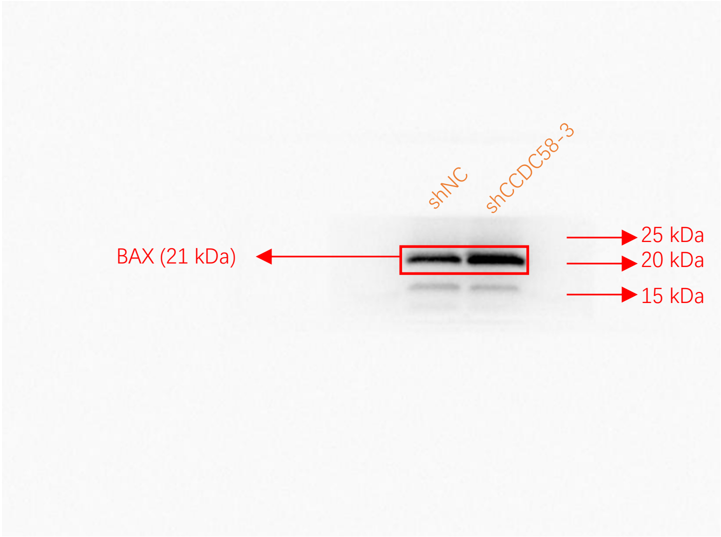

shNC
shCCDC58-3
BAX (21 kDa)
25 kDa
20 kDa
15 kDa

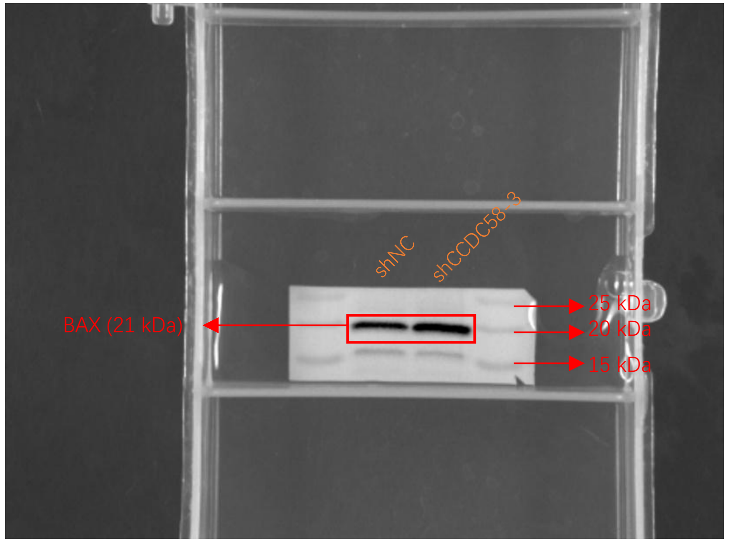

shNC
shCCDC58-3
BAX (21 kDa)
25 kDa
20 kDa
15 kDa

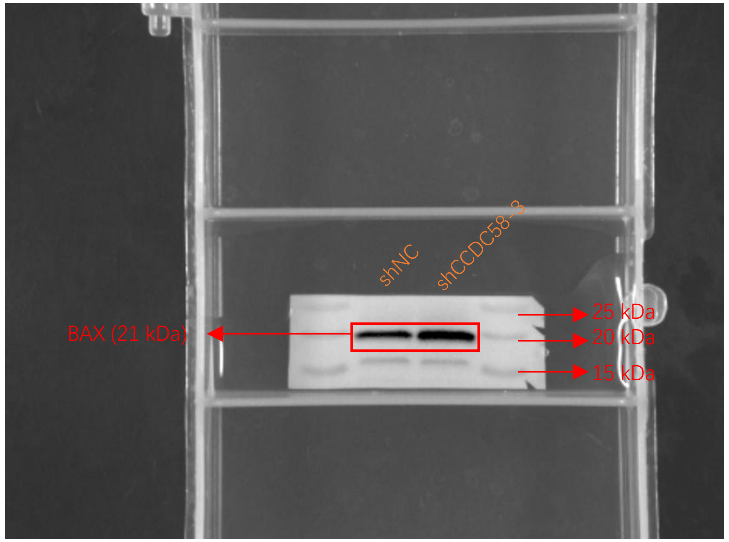

shNC
shCCDC58-3
BAX (21 kDa)
25 kDa
20 kDa
15 kDa

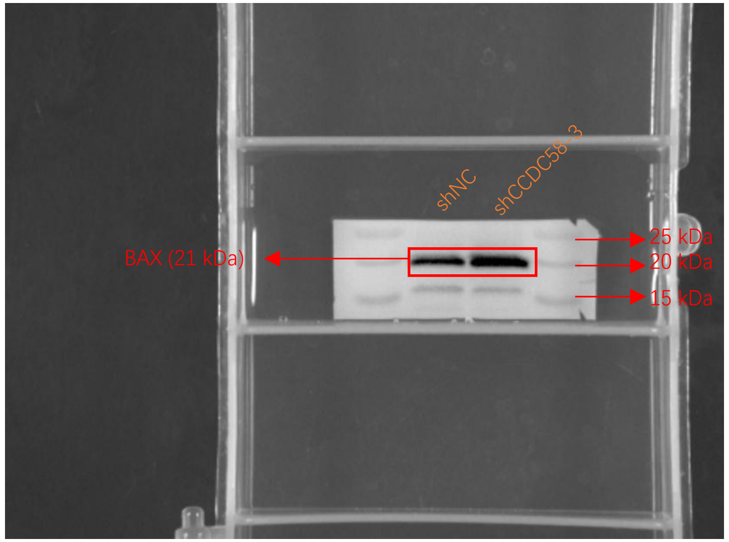

shNC
shCCDC58-3
BAX (21 kDa)
25 kDa
20 kDa
15 kDa

H1299

Internal Control of BAX

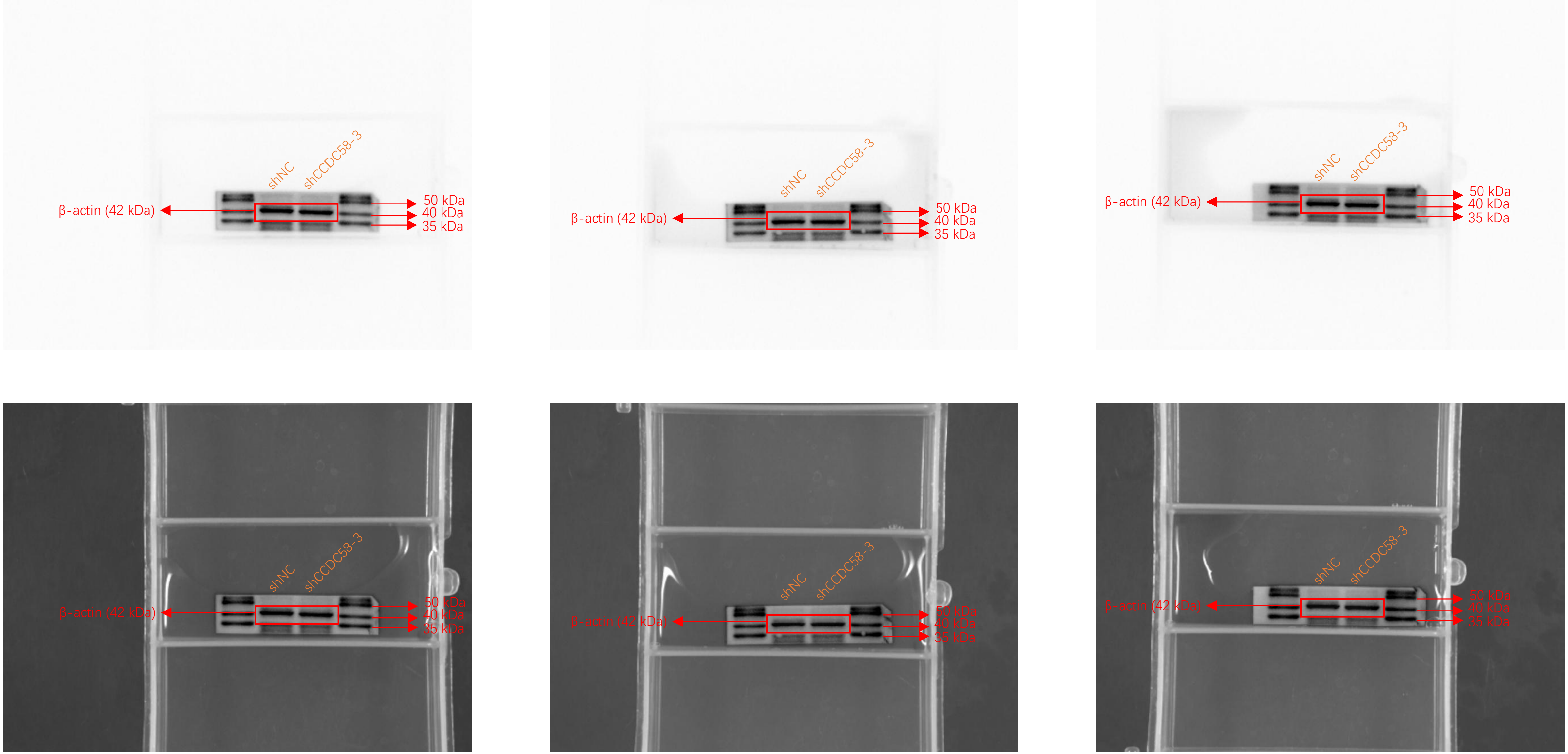

H1299

BCL-2

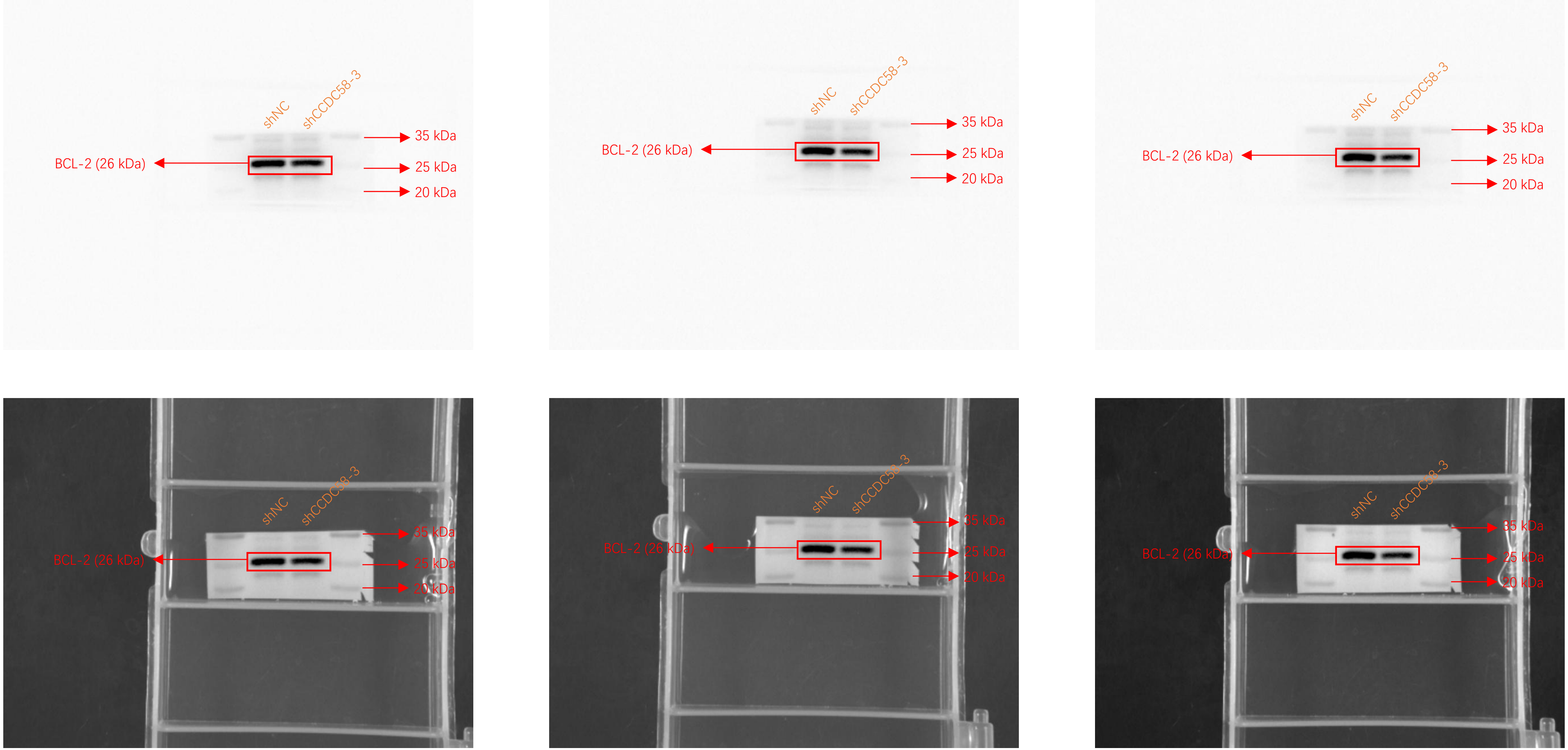

H1299

Internal Control of BCL-2

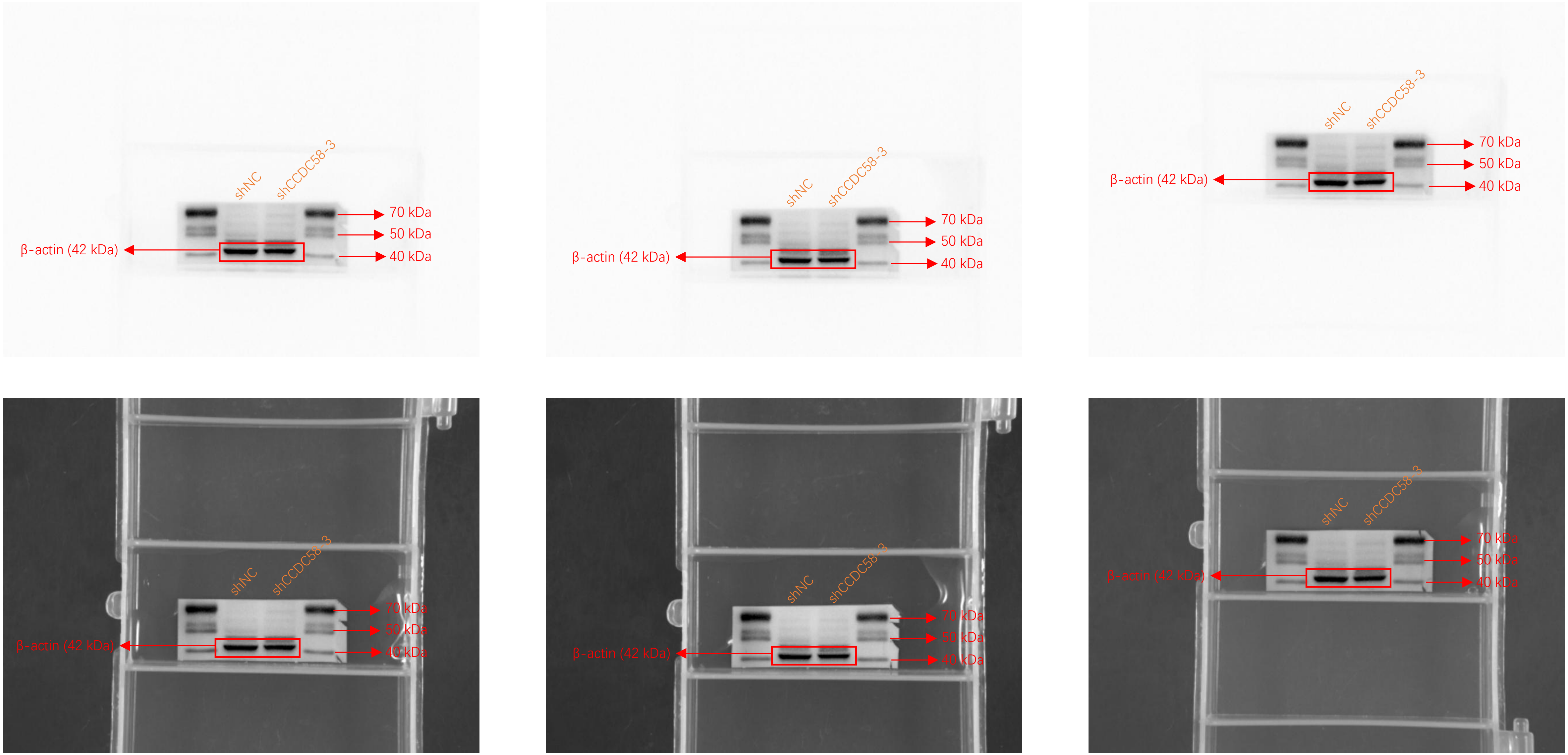

Figure 7A, B CCDC58 knockdown reduced p-PI3K and p-AKT protein expression

A549

PI3K

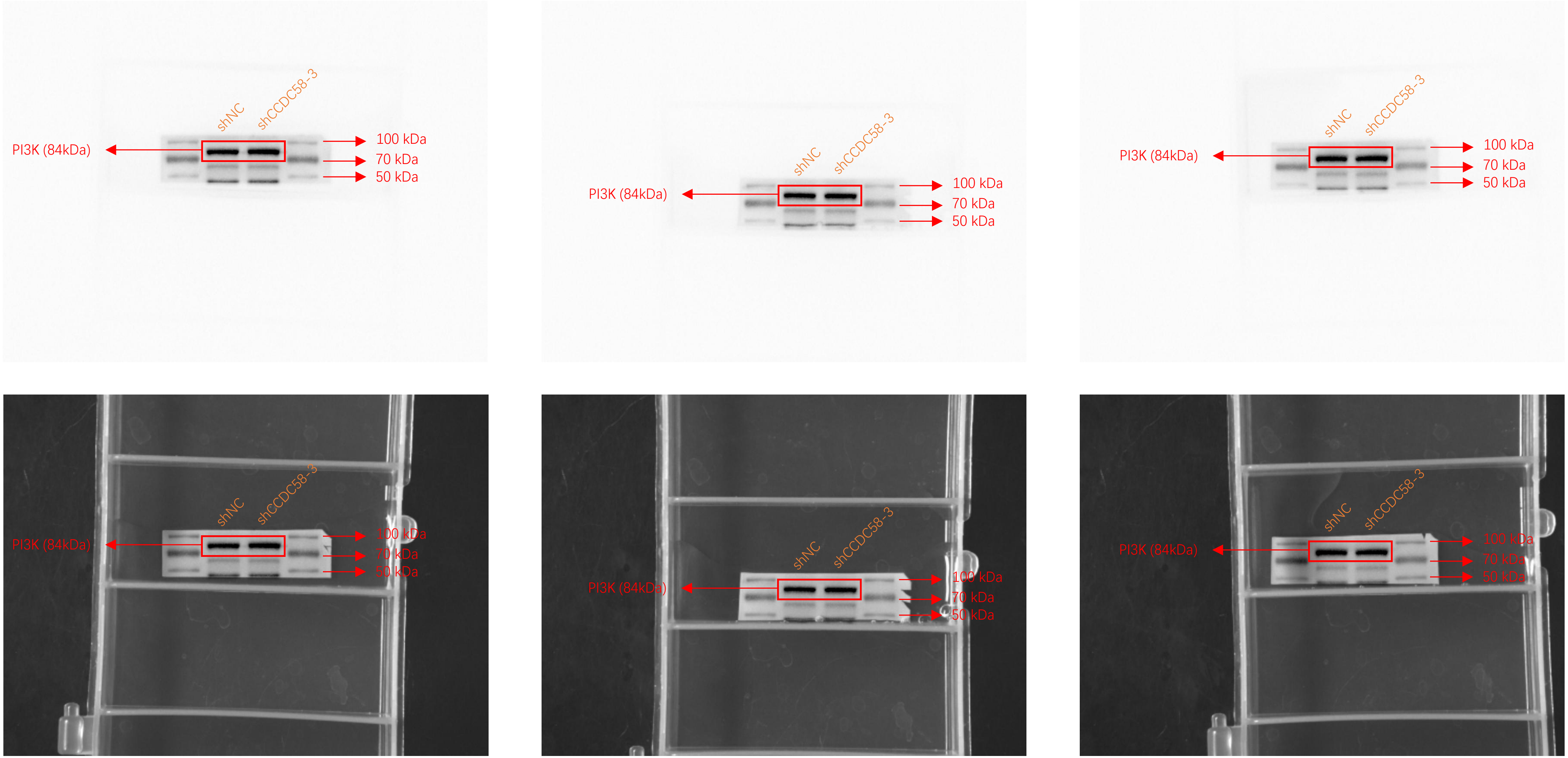

A549

Internal Control of PI3K

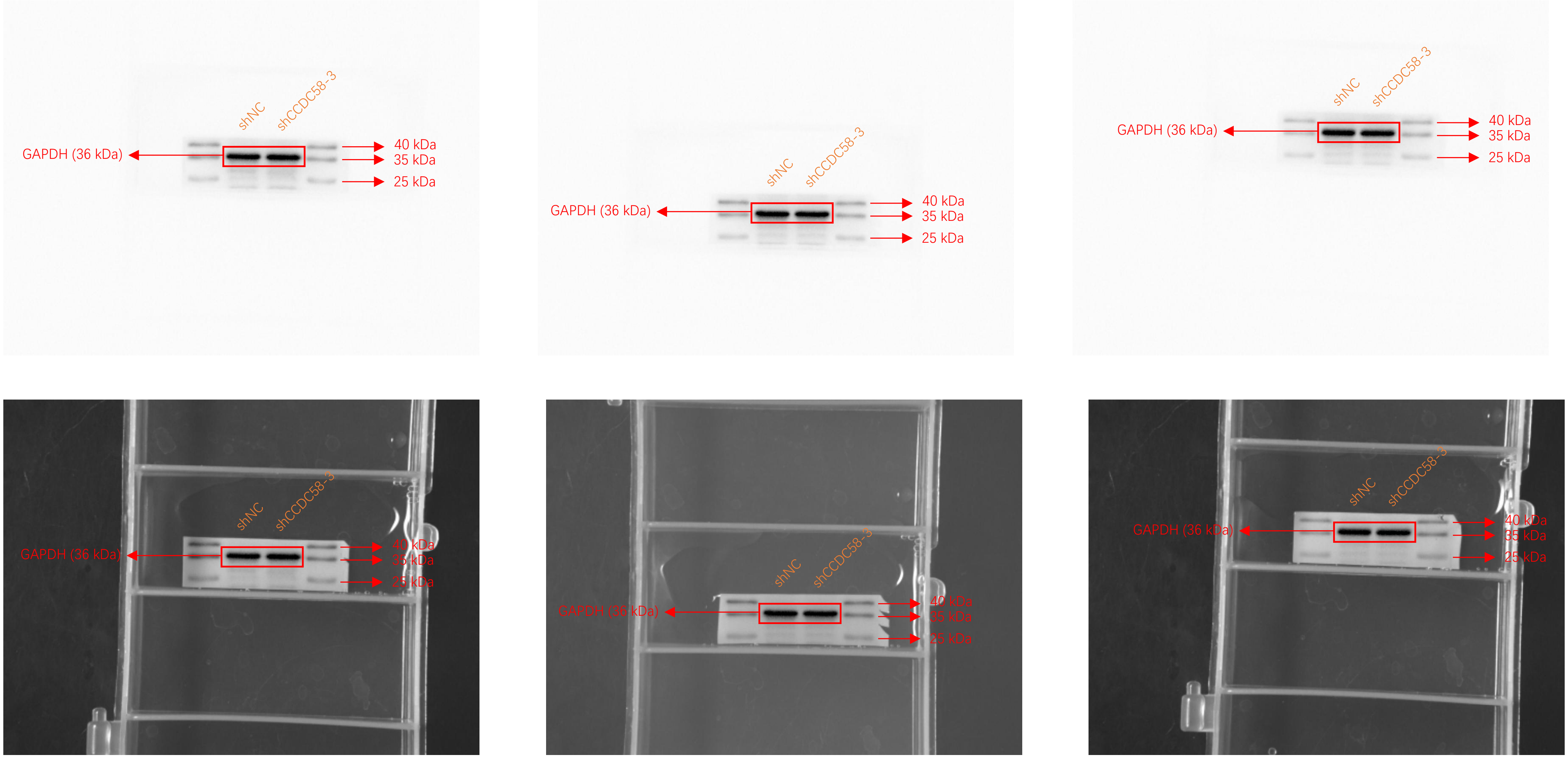

A549

p-PI3K

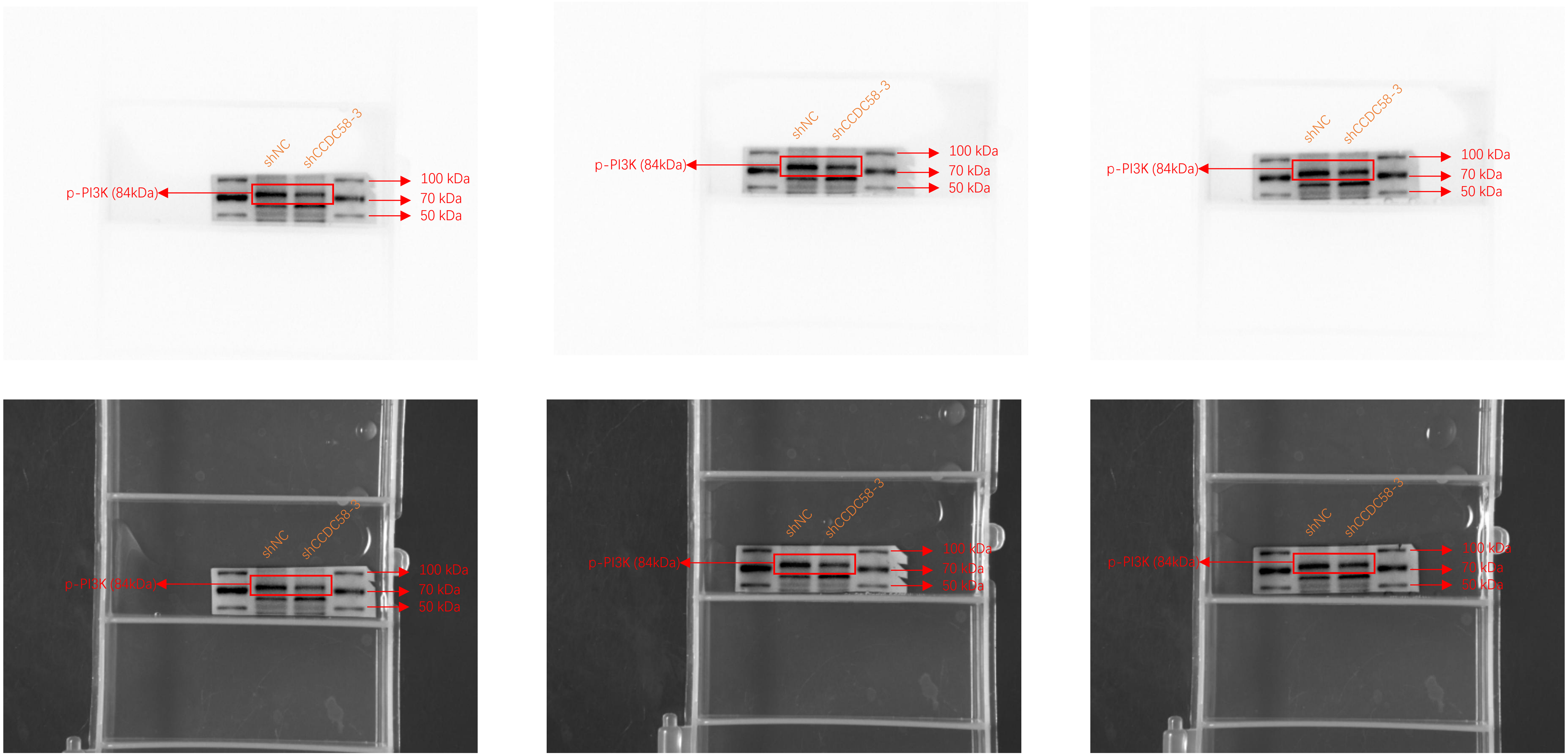

A549

Internal Control of p-PI3K

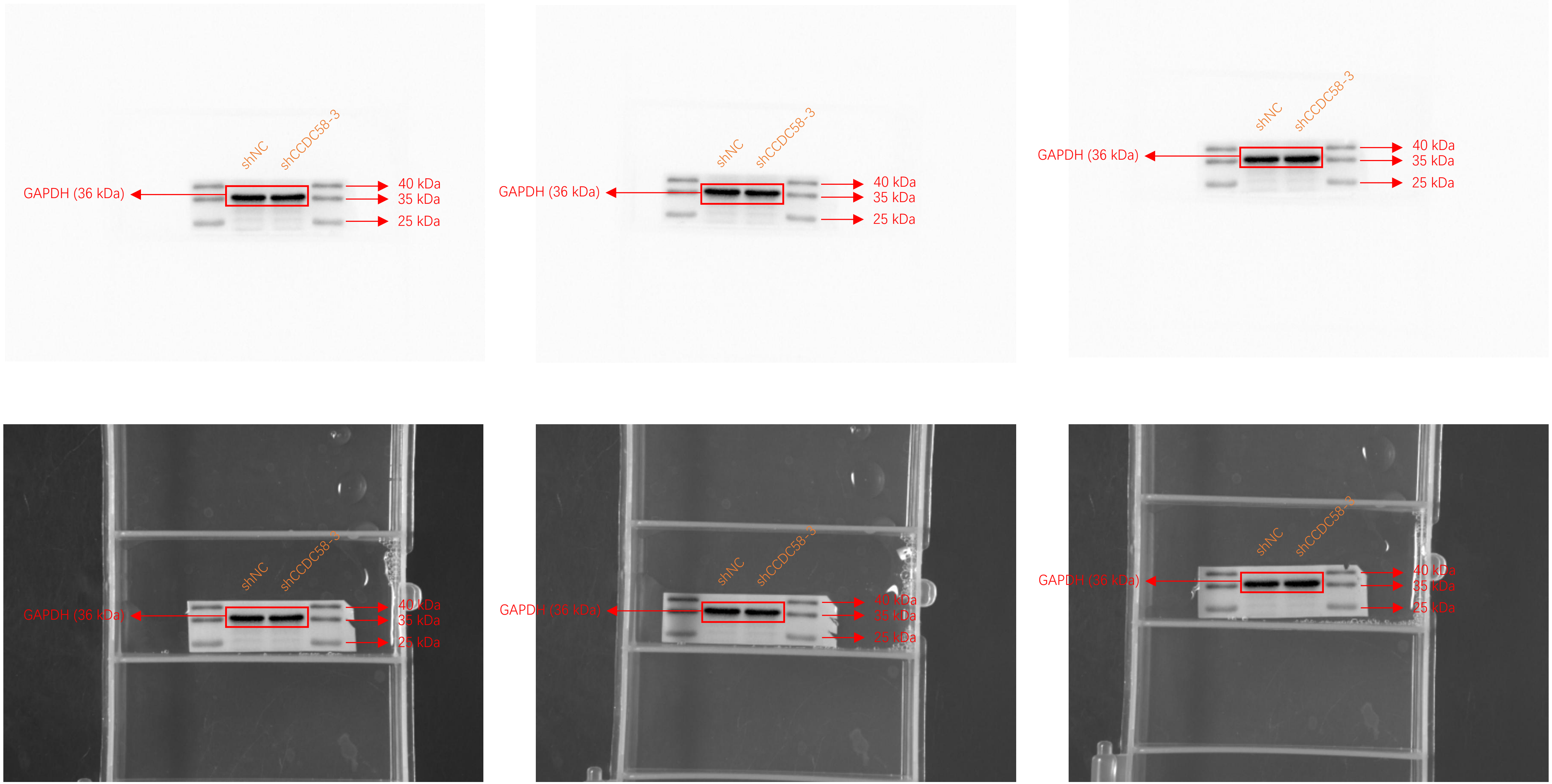

A549

AKT

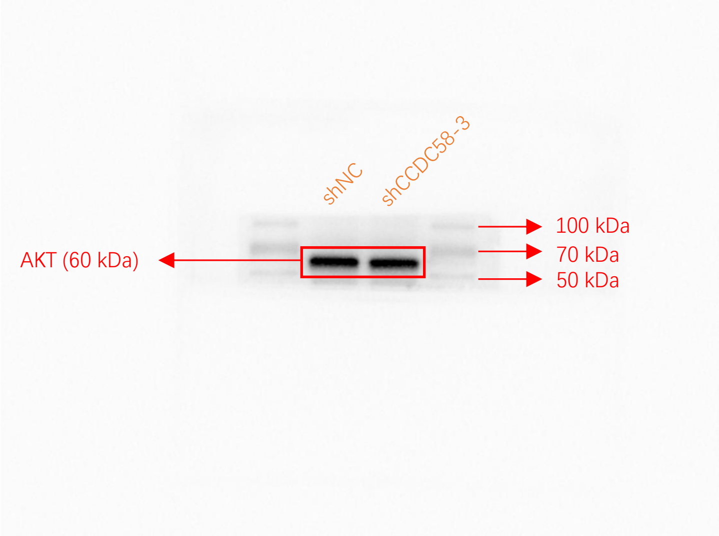
shNC
shCCDC58-3
100 kDa
70 kDa
50 kDa
AKT (60 kDa)

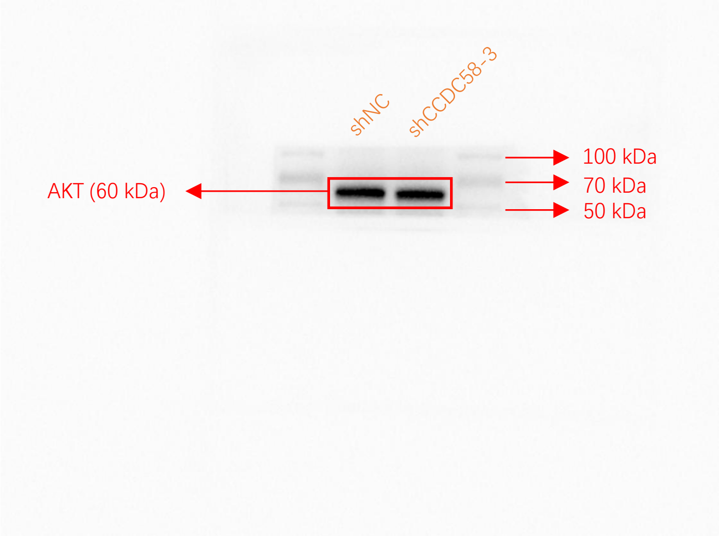
shNC
shCCDC58-3
100 kDa
70 kDa
50 kDa
AKT (60 kDa)

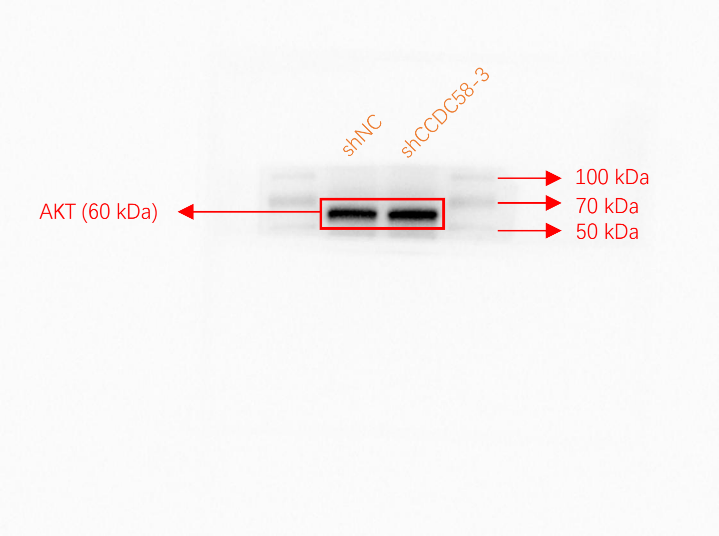
shNC
shCCDC58-3
100 kDa
70 kDa
50 kDa
AKT (60 kDa)

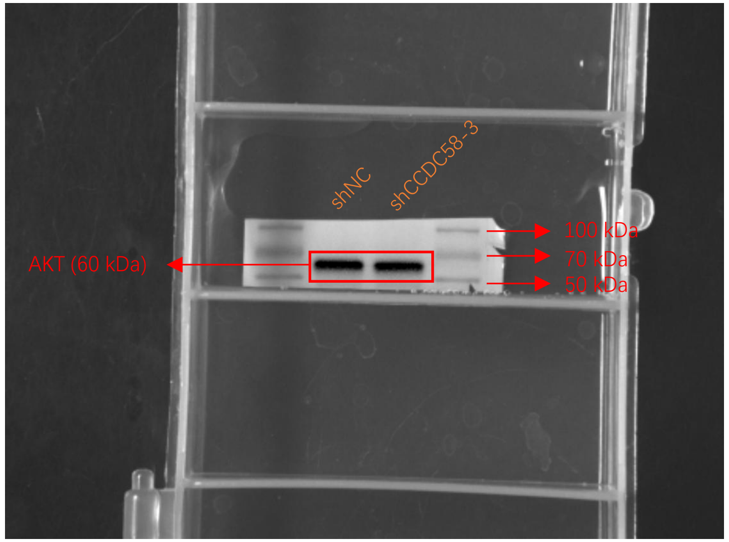
shNC
shCCDC58-3
100 kDa
70 kDa
50 kDa
AKT (60 kDa)

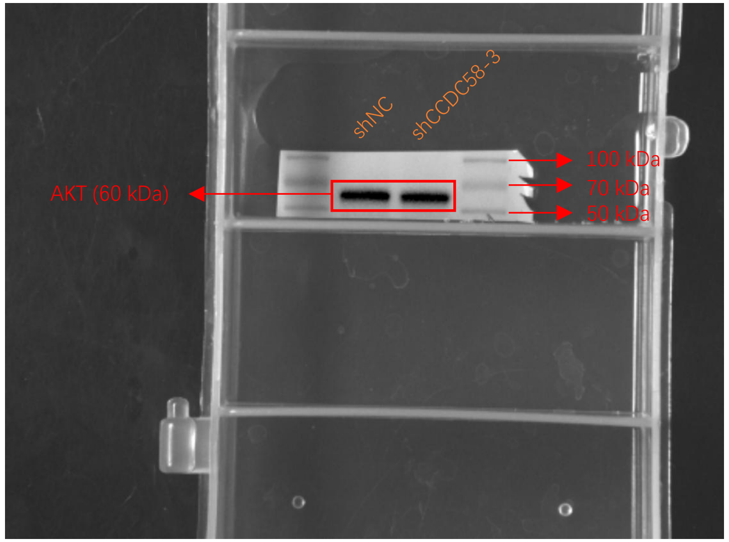
shNC
shCCDC58-3
100 kDa
70 kDa
50 kDa
AKT (60 kDa)

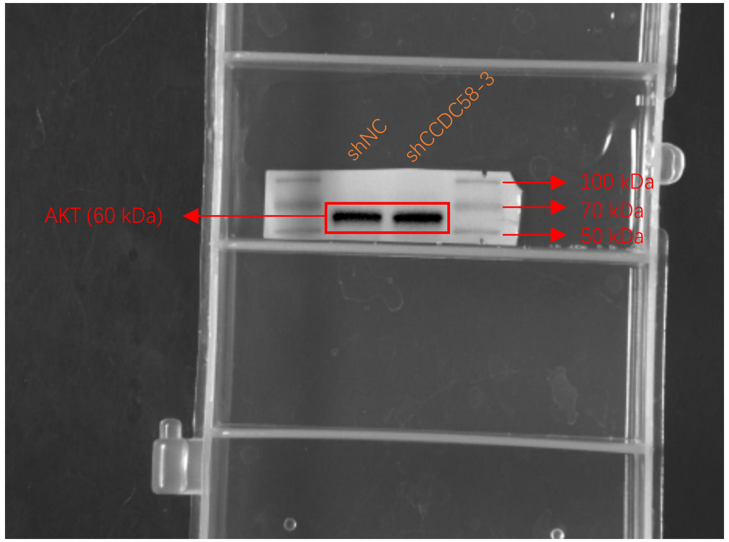
shNC
shCCDC58-3
100 kDa
70 kDa
50 kDa
AKT (60 kDa)

A549

Internal Control of AKT

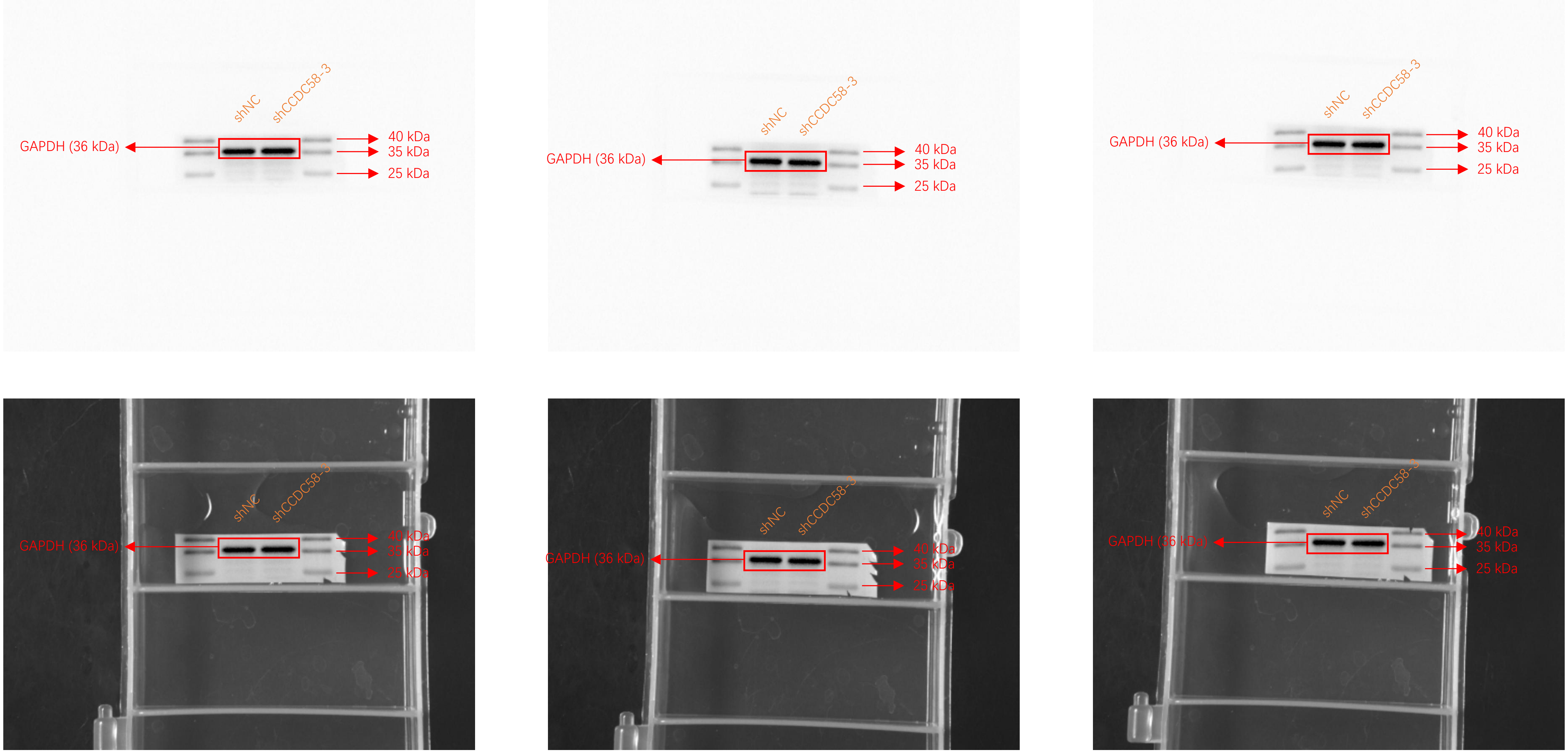

A549

p-AKT

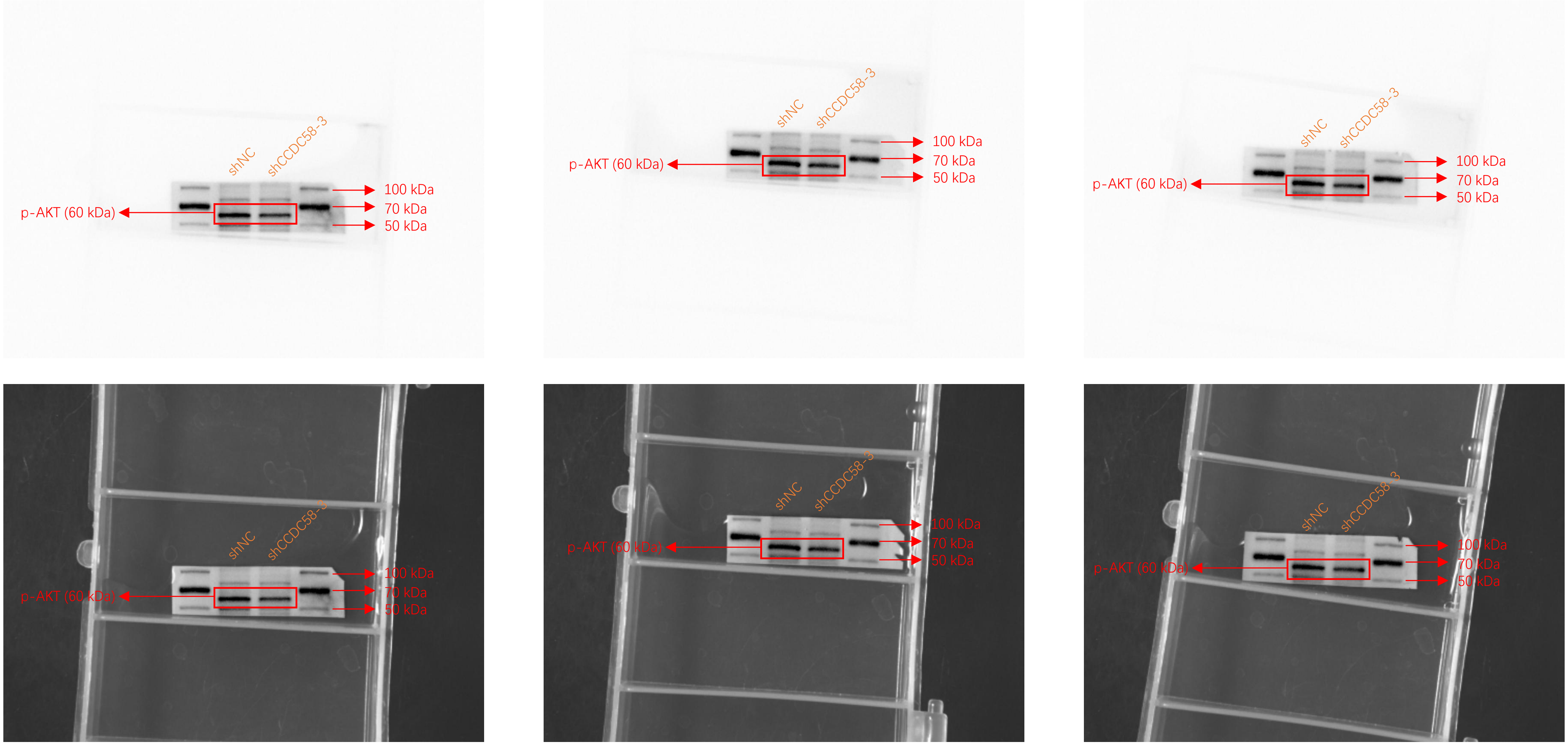

A549

Internal Control of p-AKT

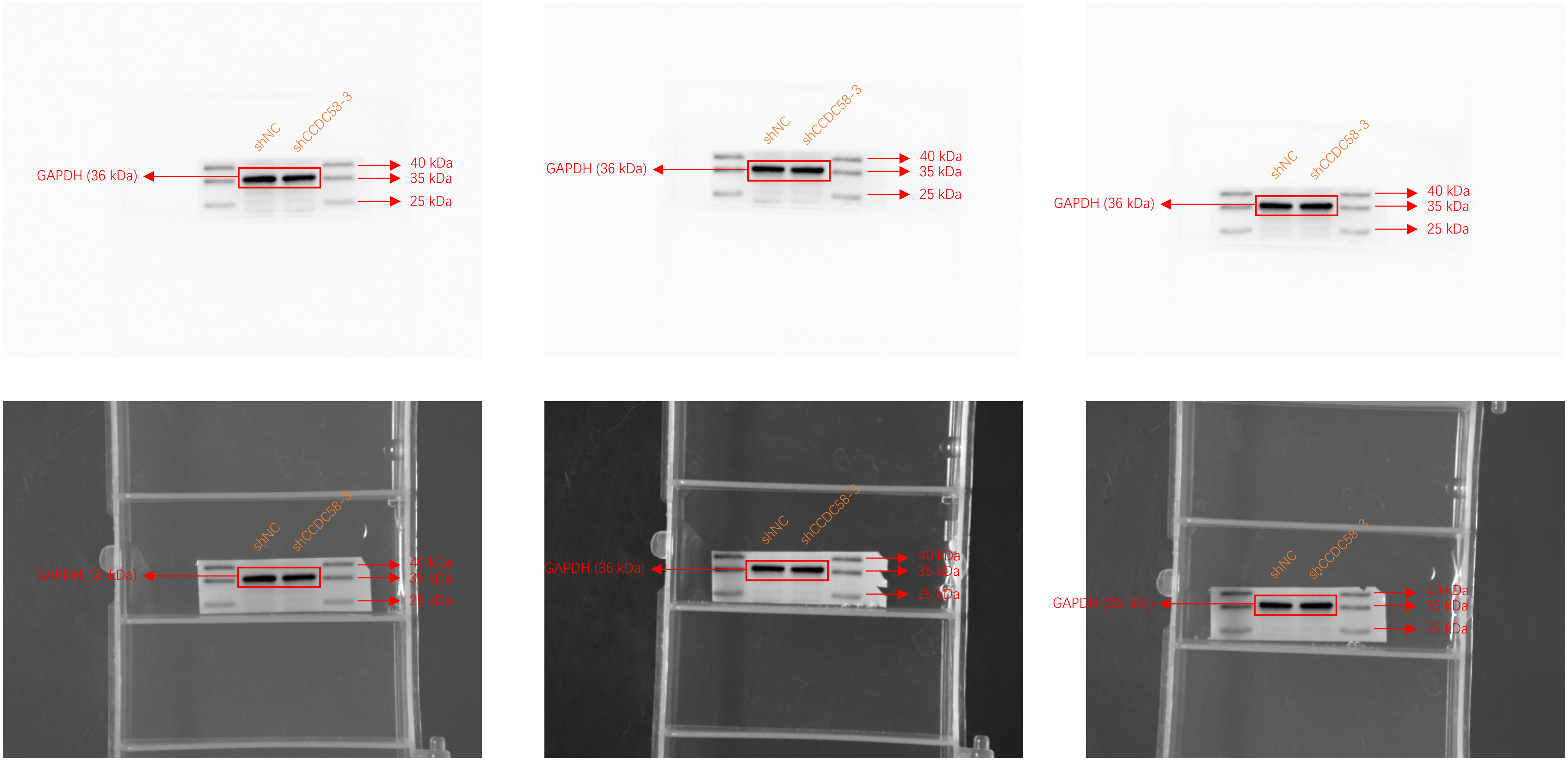

H1299

PI3K

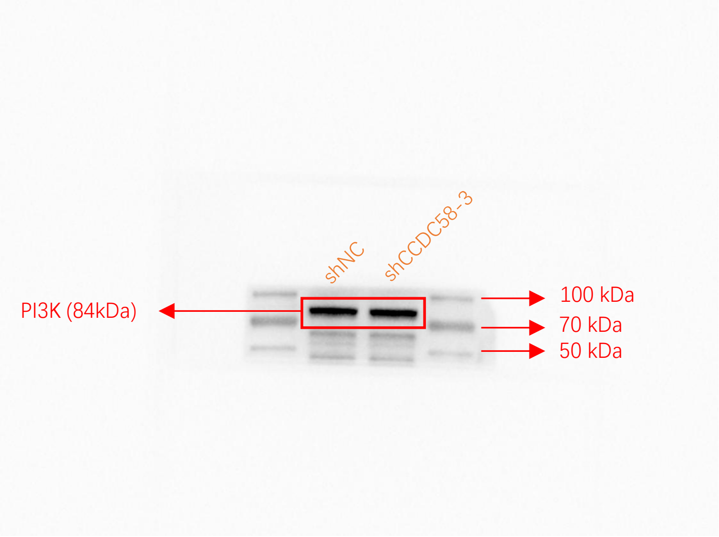
shNC
shCCDC58-3
PI3K (84kDa)
100 kDa
70 kDa
50 kDa

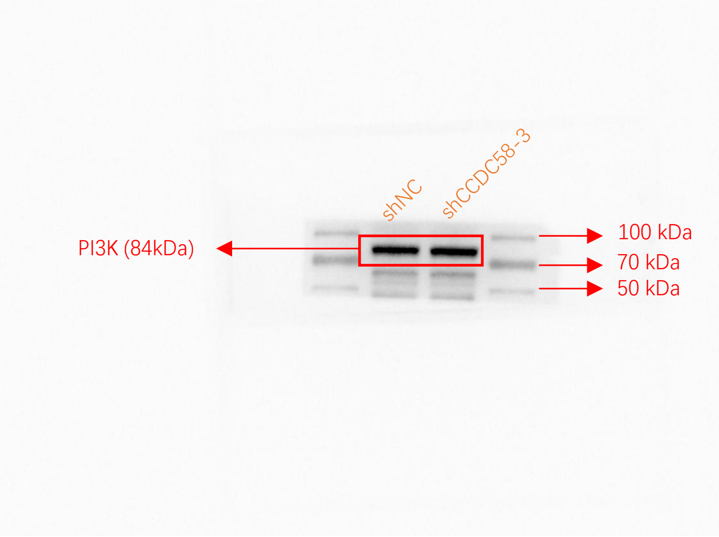
shNC
shCCDC58-3
PI3K (84kDa)
100 kDa
70 kDa
50 kDa

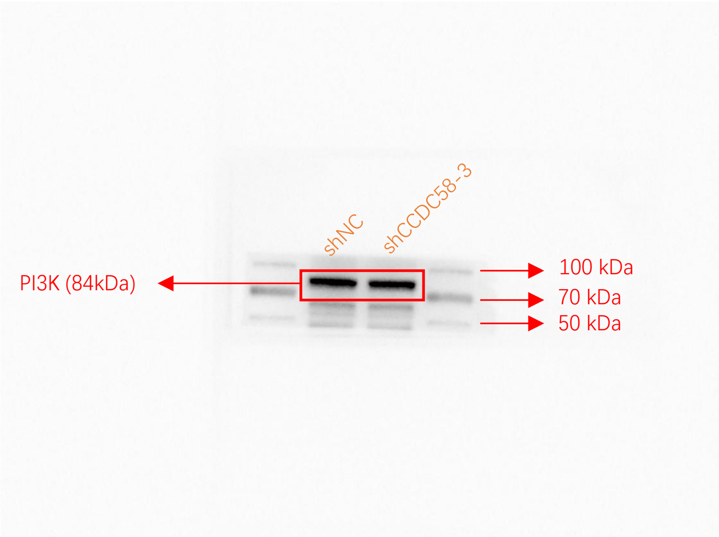
shNC
shCCDC58-3
PI3K (84kDa)
100 kDa
70 kDa
50 kDa

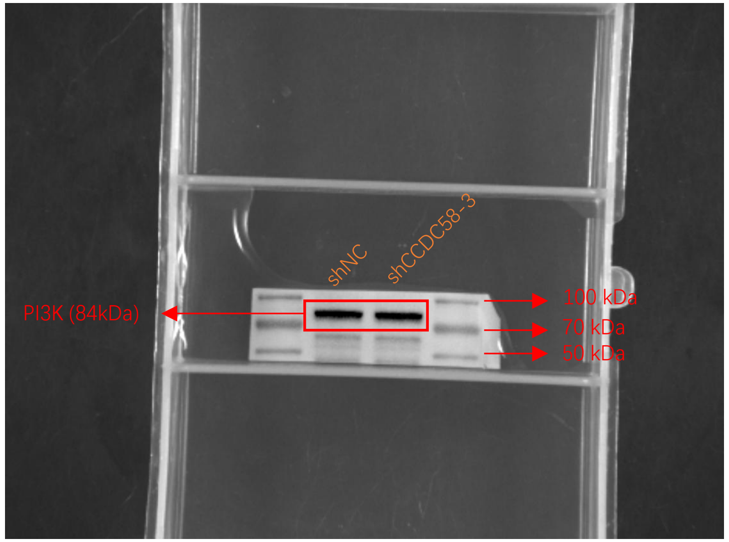
shNC
shCCDC58-3
PI3K (84kDa)
100 kDa
70 kDa
50 kDa

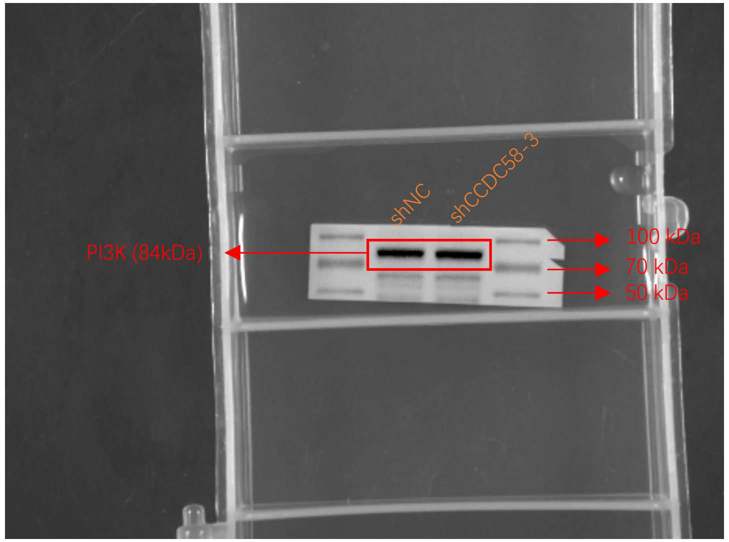
shNC
shCCDC58-3
PI3K (84kDa)
100 kDa
70 kDa
50 kDa

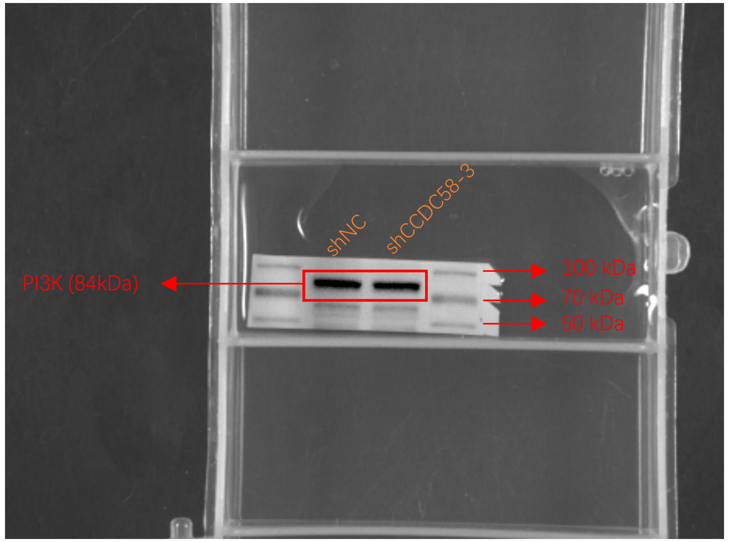
shNC
shCCDC58-3
PI3K (84kDa)
100 kDa
70 kDa
50 kDa

H1299

Internal Control of PI3K

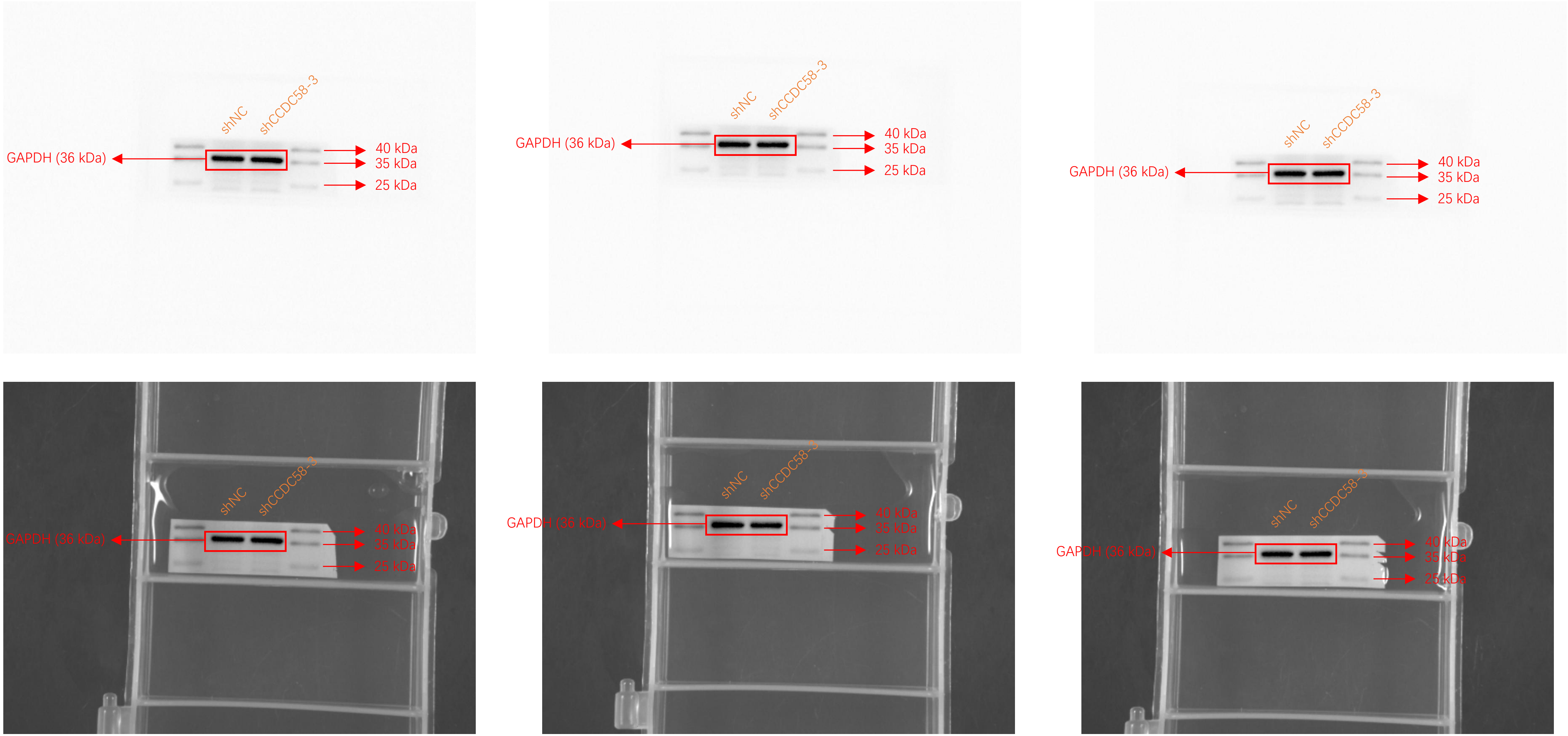

H1299

p-PI3K

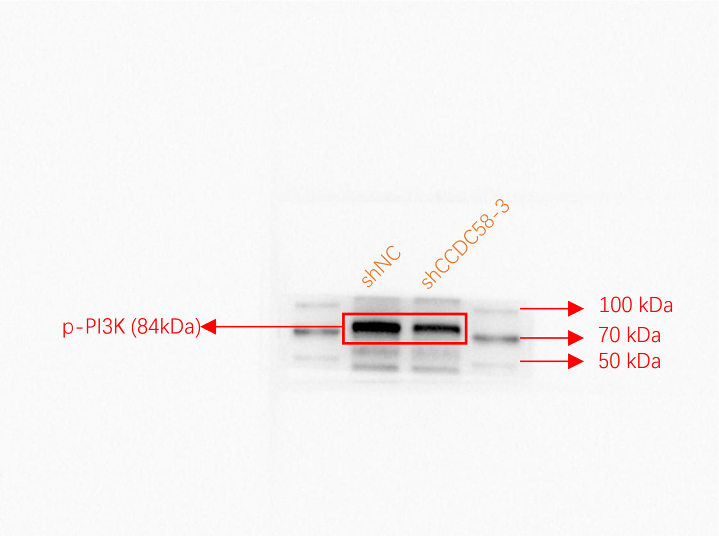
shNC
shCCDC58-3
p-PI3K (84kDa)
100 kDa
70 kDa
50 kDa

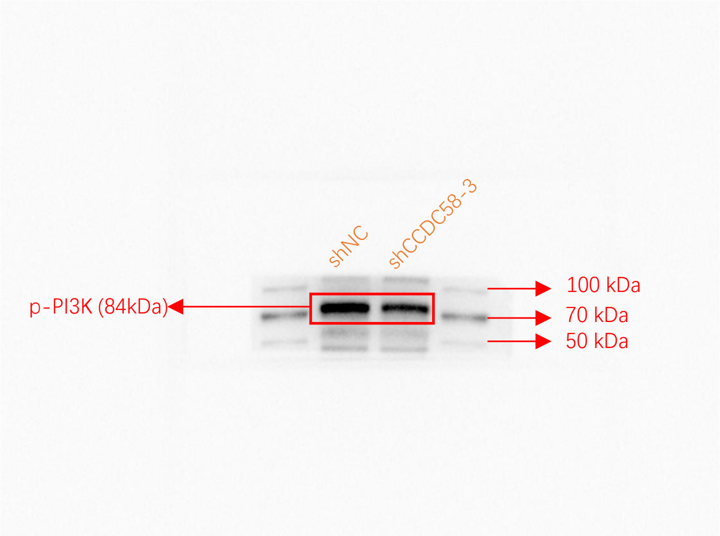
shNC
shCCDC58-3
p-PI3K (84kDa)
100 kDa
70 kDa
50 kDa

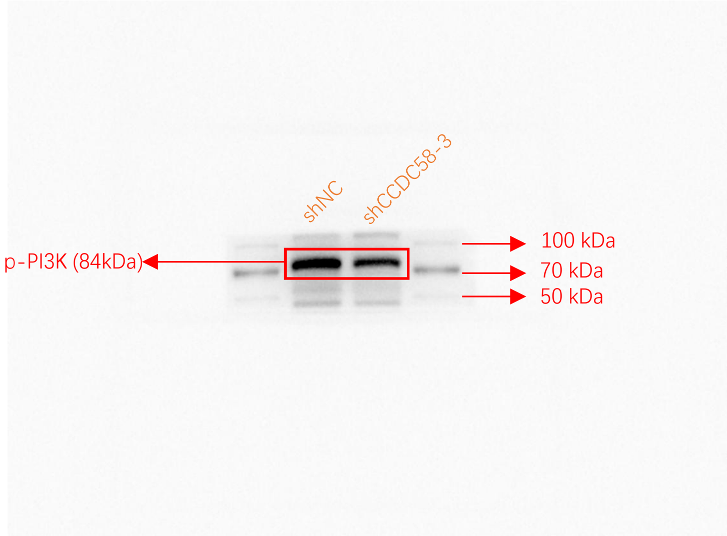
shNC
shCCDC58-3
p-PI3K (84kDa)
100 kDa
70 kDa
50 kDa

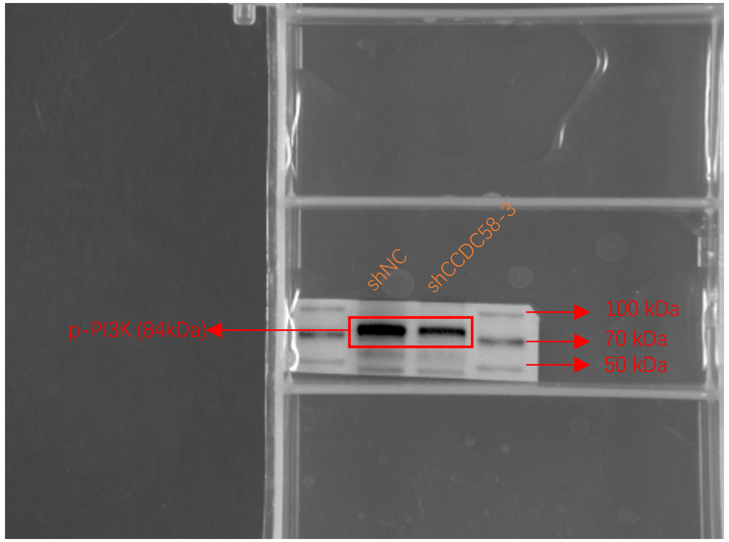
shNC
shCCDC58-3
p-PI3K (84kDa)
100 kDa
70 kDa
50 kDa

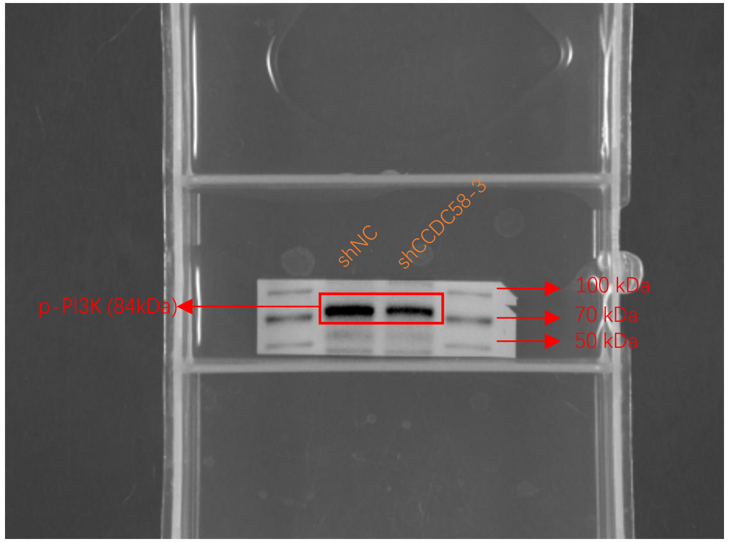
shNC
shCCDC58-3
p-PI3K (84kDa)
100 kDa
70 kDa
50 kDa

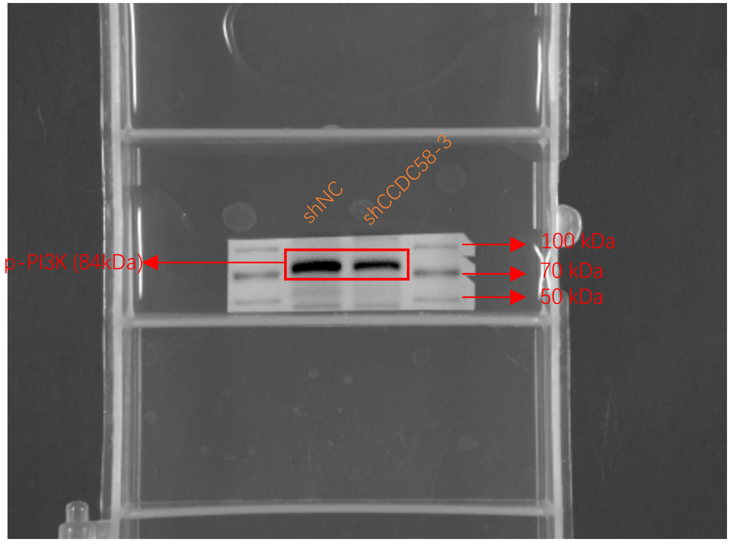
shNC
shCCDC58-3
p-PI3K (84kDa)
100 kDa
70 kDa
50 kDa

H1299

Internal Control of p-PI3K

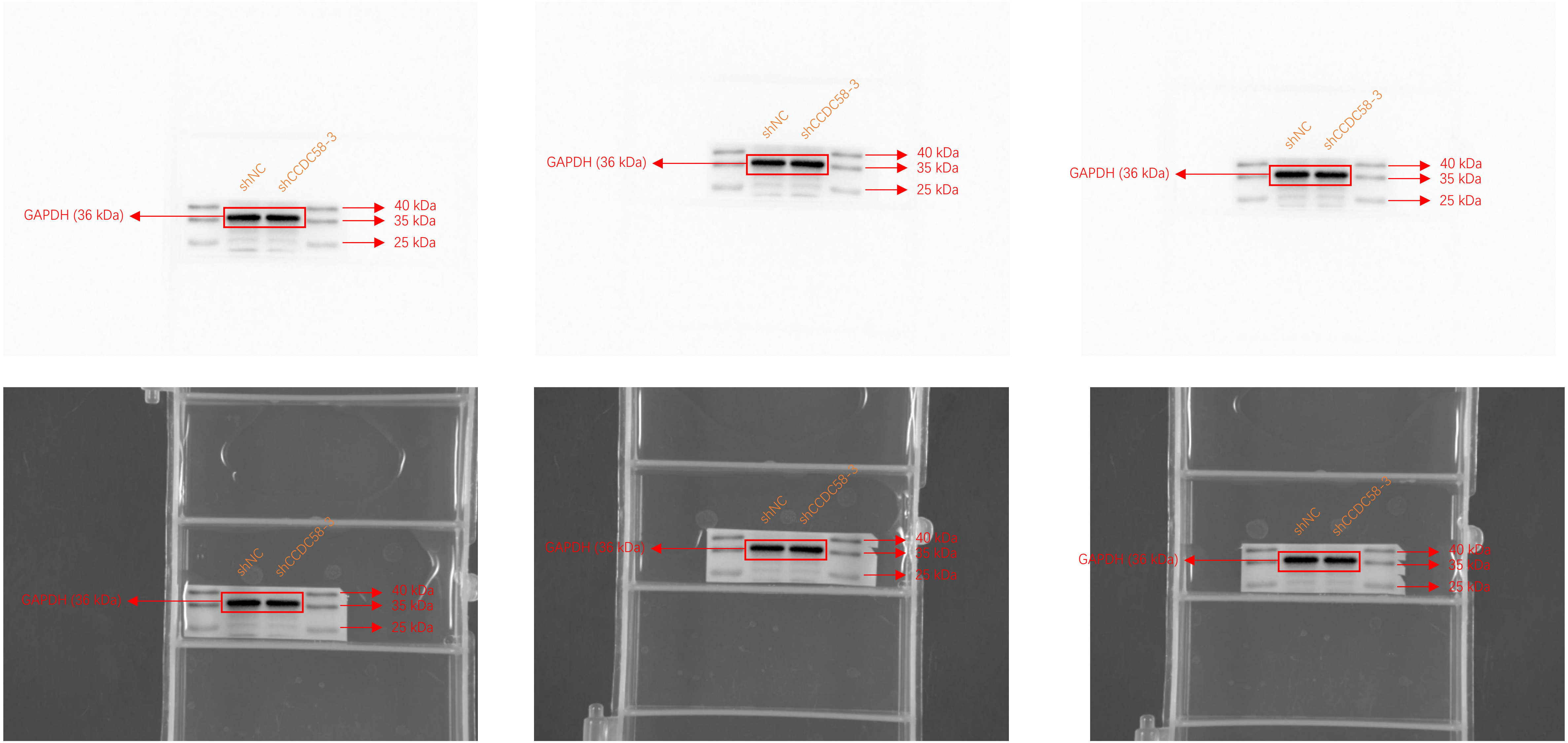

H1299

AKT

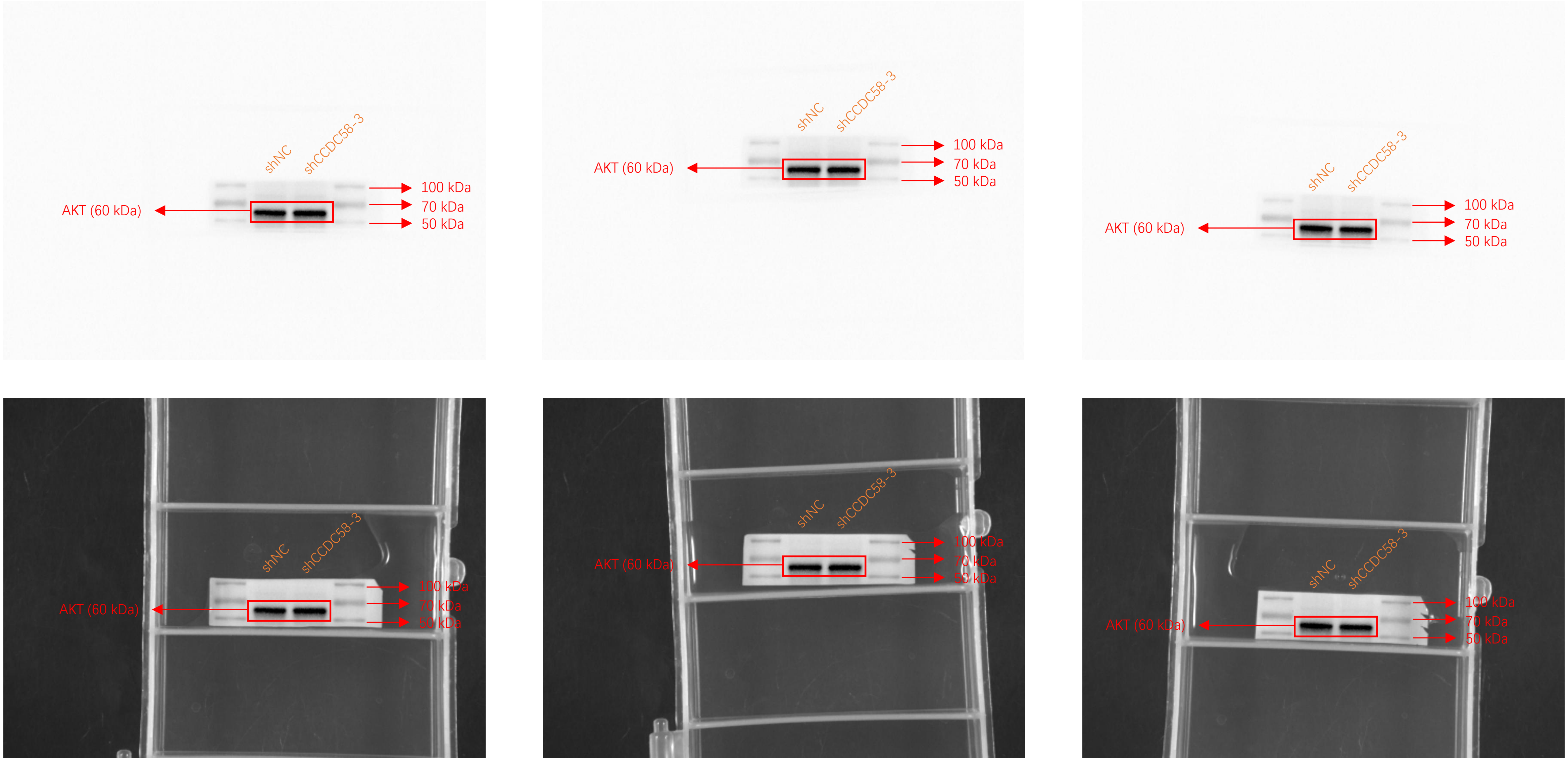

H1299

Internal Control of AKT

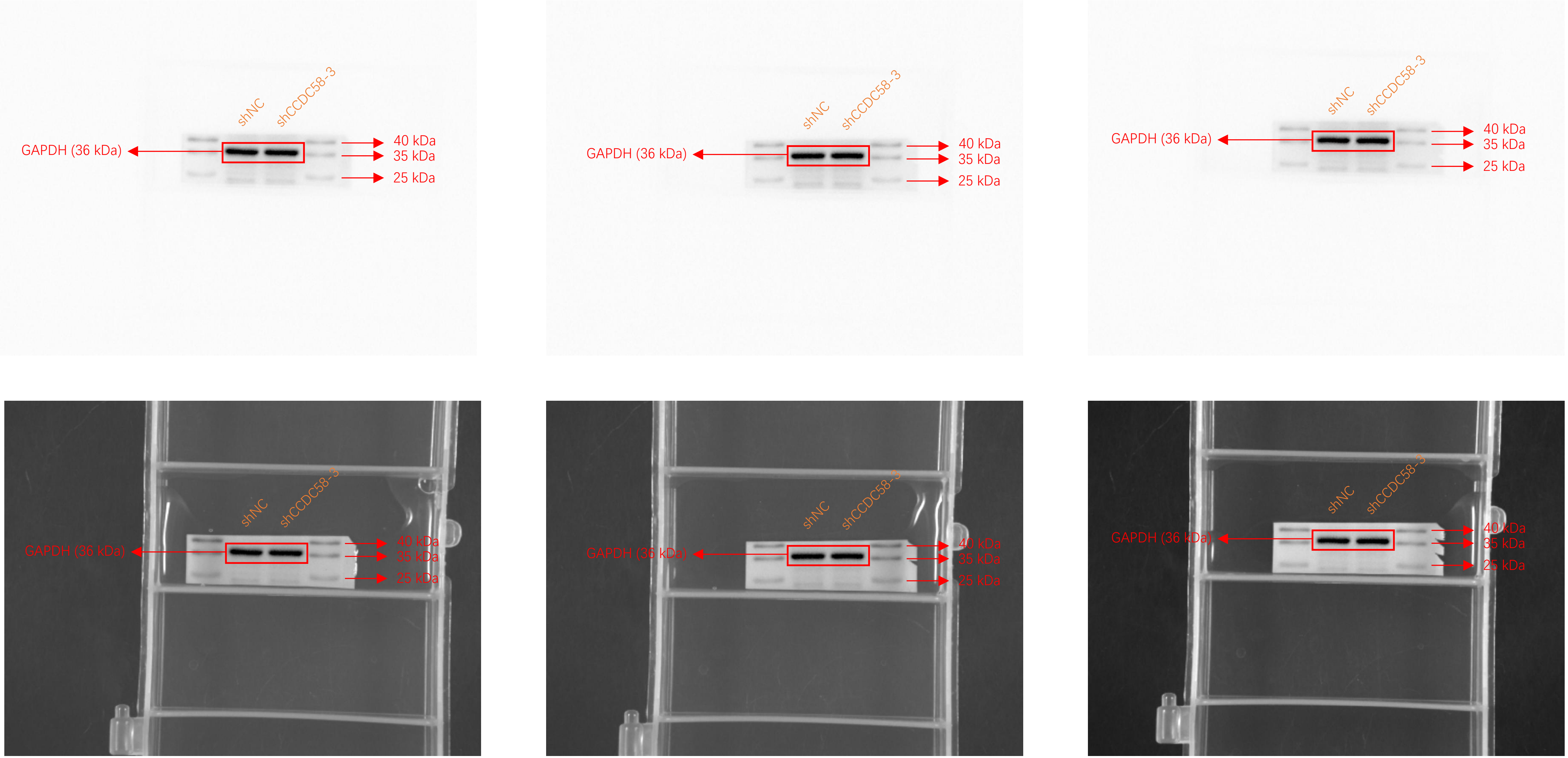

H1299

p-AKT

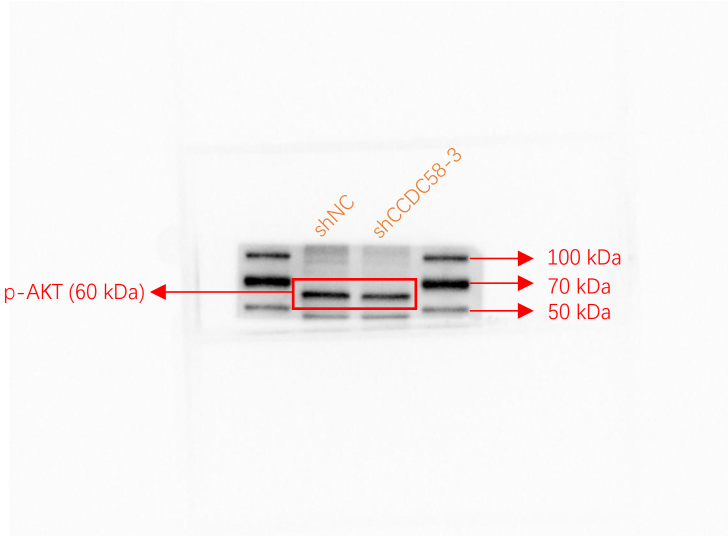
shNC
shCCDC58-3
100 kDa
70 kDa
50 kDa
p-AKT (60 kDa)

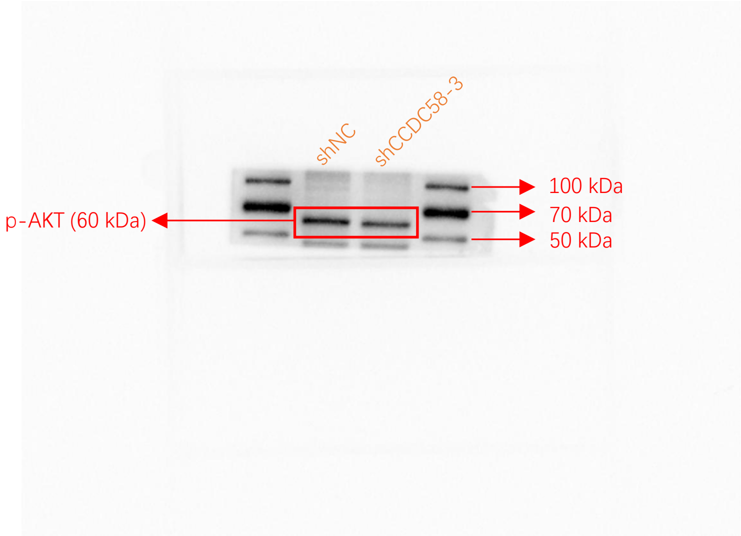
shNC
shCCDC58-3
100 kDa
70 kDa
50 kDa
p-AKT (60 kDa)

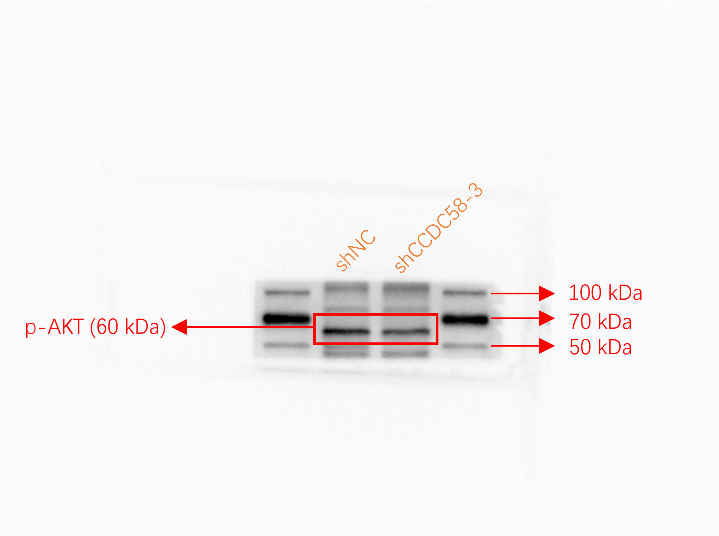
shNC
shCCDC58-3
100 kDa
70 kDa
50 kDa
p-AKT (60 kDa)

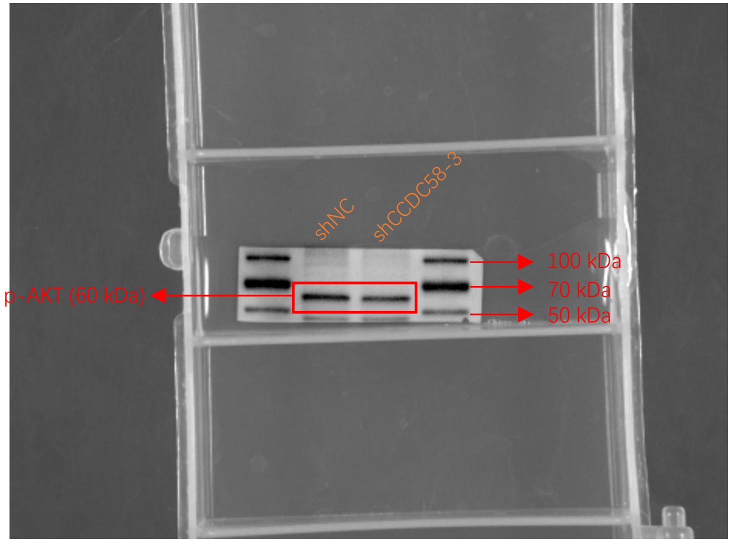
shNC
shCCDC58-3
100 kDa
70 kDa
50 kDa
p-AKT (60 kDa)

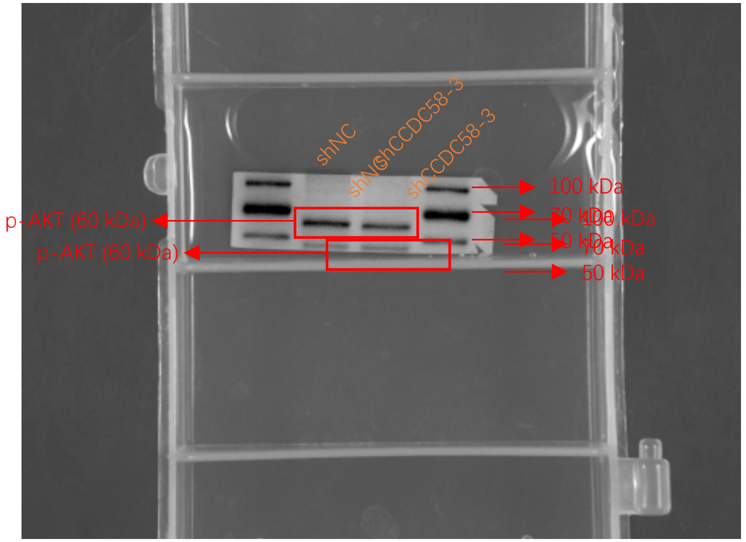
shNC
shCCDC58-3
100 kDa
p-AKT (60 kDa)

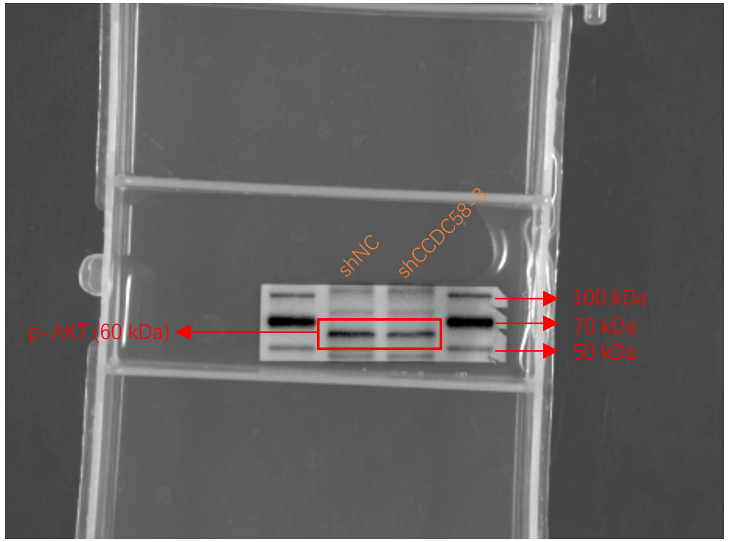
shNC
100 kDa
70 kDa
50 kDa
p-AKT (60 kDa)

H1299

Internal Control of p-AKT

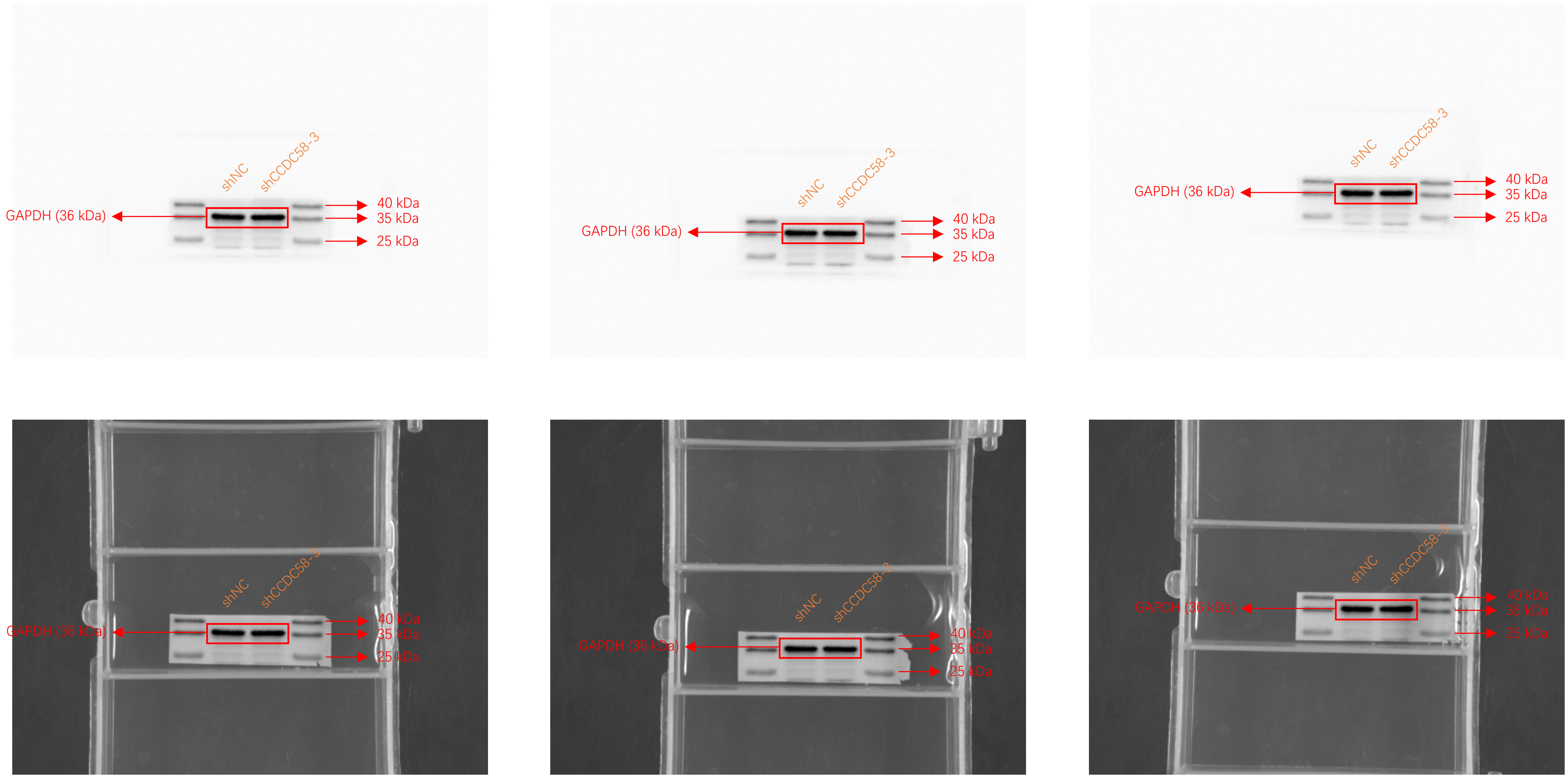

Supplement: Supplementary file 2 [file DataSheet2.pdf]
